# Supplementary material for: Artificial intelligence for surgical scene understanding: a systematic review and reporting quality meta-analysis
Source: NPJ Digit Med. 2025 Dec 17;9:59. doi: 10.1038/s41746-025-02227-4 (PMC12820105; doi:10.1038/s41746-025-02227-4)
Supplement: Supplementary file 1 — Supplementary Material [file 41746_2025_2227_MOESM1_ESM.pdf]

Artificial Intelligence for Surgical Scene Understanding: A Systematic Review and Reporting Quality Meta-Analysis

Matthias Carstens, Shubha Vasisht, Zheyuan Zhang, Iulia Barbur, Annika Reinke, Lena Maier-Hein, Daniel A. Hashimoto, Fiona R. Kolbinger

Supplementary Material

Table of Contents

Supplementary Table 1*: PRISMA Checklist* *2*

Supplementary Table 2*: Search Strategy* *5*

Supplementary Table 3*: Studies and study characteristics included in the systematic review and meta-analysis (1)* *6*

Supplementary Table 4*: Studies and study characteristics included in the systematic review and meta-analysis (2)* *18*

Supplementary Table 5*: Studies and study characteristics included in the systematic review and meta-analysis (3)* *27*

Supplementary Figure 1*: Overview of Findings by Category* *30*

Supplementary Table 6*: Clinical Translation* *31*

Supplementary Table 7*: Reporting Quality* *32*

Supplementary Table 8*: Reporting Quality Across JCR-Based Journal Categories* *35*

Supplementary Figure 2*: Reporting Quality Across JCR-Based Journal Categories* *36*

References of all included studies *37*

Supplementary Table 1: PRISMA Checklist

Section and Topic	Item #	Checklist item	Location where item is reported
**TITLE**			
Title	1	Identify the report as a systematic review.	Page 1
**ABSTRACT**			
Abstract	2	See the PRISMA 2020 for Abstracts checklist.	Page 2
**INTRODUCTION**			
Rationale	3	Describe the rationale for the review in the context of existing knowledge.	Pages 2-3
Objectives	4	Provide an explicit statement of the objective(s) or question(s) the review addresses.	Pages 2-3
**METHODS**			
Eligibility criteria	5	Specify the inclusion and exclusion criteria for the review and how studies were grouped for the syntheses.	Page 15-16
Information sources	6	Specify all databases, registers, websites, organisations, reference lists and other sources searched or consulted to identify studies. Specify the date when each source was last searched or consulted.	Page 15
Search strategy	7	Present the full search strategies for all databases, registers and websites, including any filters and limits used.	Suppl. Tab. 2
Selection process	8	Specify the methods used to decide whether a study met the inclusion criteria of the review, including how many reviewers screened each record and each report retrieved, whether they worked independently, and if applicable, details of automation tools used in the process.	Page 15
Data collection process	9	Specify the methods used to collect data from reports, including how many reviewers collected data from each report, whether they worked independently, any processes for obtaining or confirming data from study investigators, and if applicable, details of automation tools used in the process.	Page 15
Data items	10a	List and define all outcomes for which data were sought. Specify whether all results that were compatible with each outcome domain in each study were sought (e.g. for all measures, time points, analyses), and if not, the methods used to decide which results to collect.	Pages 16-17
	10b	List and define all other variables for which data were sought (e.g. participant and intervention characteristics, funding sources). Describe any assumptions made about any missing or unclear information.	Pages 16-17
Study risk of bias assessment	11	Specify the methods used to assess risk of bias in the included studies, including details of the tool(s) used, how many reviewers assessed each study and whether they worked independently, and if applicable, details of automation tools used in the process.	Pages 15, 17
Effect measures	12	Specify for each outcome the effect measure(s) (e.g. risk ratio, mean difference) used in the synthesis or presentation of results.	Page 17
Synthesis methods	13a	Describe the processes used to decide which studies were eligible for each synthesis (e.g. tabulating the study intervention characteristics and comparing against the planned groups for each synthesis (item #5)).	Pages 15-17
	13b	Describe any methods required to prepare the data for presentation or synthesis, such as handling of missing summary statistics, or data conversions.	Page 16
	13c	Describe any methods used to tabulate or visually display results of individual studies and syntheses.	Page 16
	13d	Describe any methods used to synthesize results and provide a rationale for the choice(s). If meta-analysis was performed, describe the model(s), method(s) to identify the presence and extent of statistical heterogeneity, and software package(s) used.	Pages 16-17
	13e	Describe any methods used to explore possible causes of heterogeneity among study results (e.g. subgroup analysis, meta-regression).	Pages 16-17

Section and Topic	Item #	Checklist item	Location where item is reported
	13f	Describe any sensitivity analyses conducted to assess robustness of the synthesized results.	Page 16-17
Reporting bias assessment	14	Describe any methods used to assess risk of bias due to missing results in a synthesis (arising from reporting biases).	Page 16-17
Certainty assessment	15	Describe any methods used to assess certainty (or confidence) in the body of evidence for an outcome.	Page 16-17
**RESULTS**			
Study selection	16a	Describe the results of the search and selection process, from the number of records identified in the search to the number of studies included in the review, ideally using a flow diagram.	Page 4, Fig. 1
	16b	Cite studies that might appear to meet the inclusion criteria, but which were excluded, and explain why they were excluded.	N/A
Study characteristics	17	Cite each included study and present its characteristics.	Suppl. Tab. 3-7
Risk of bias in studies	18	Present assessments of risk of bias for each included study.	Page 10, Suppl. Tab. 8
Results of individual studies	19	For all outcomes, present, for each study: (a) summary statistics for each group (where appropriate) and (b) an effect estimate and its precision (e.g. confidence/credible interval), ideally using structured tables or plots.	Fig 3, 4, 5, Suppl. Tab. 3-7
Results of syntheses	20a	For each synthesis, briefly summarise the characteristics and risk of bias among contributing studies.	Fig. 3-5, Supp. Fig. 1-2, Pages 4-10
	20b	Present results of all statistical syntheses conducted. If meta-analysis was done, present for each the summary estimate and its precision (e.g. confidence/credible interval) and measures of statistical heterogeneity. If comparing groups, describe the direction of the effect.	Fig. 3-5, Supp. Fig. 1-2, Pages 9-10
	20c	Present results of all investigations of possible causes of heterogeneity among study results.	N/A
	20d	Present results of all sensitivity analyses conducted to assess the robustness of the synthesized results.	N/A
Reporting biases	21	Present assessments of risk of bias due to missing results (arising from reporting biases) for each synthesis assessed.	N/A
Certainty of evidence	22	Present assessments of certainty (or confidence) in the body of evidence for each outcome assessed.	Suppl. Tab. 8 & Fig. 2
**DISCUSSION**			
Discussion	23a	Provide a general interpretation of the results in the context of other evidence.	Pages 12-14
	23b	Discuss any limitations of the evidence included in the review.	Page 13
	23c	Discuss any limitations of the review processes used.	Page 13
	23d	Discuss implications of the results for practice, policy, and future research.	Pages 12-14
**OTHER INFORMATION**			
Registration and protocol	24a	Provide registration information for the review, including register name and registration number, or state that the review was not registered.	Page 15
	24b	Indicate where the review protocol can be accessed, or state that a protocol was not prepared.	Page 15
	24c	Describe and explain any amendments to information provided at registration or in the protocol.	Page 15

Section and Topic	Item #	Checklist item	Location where item is reported
Support	25	Describe sources of financial or non-financial support for the review, and the role of the funders or sponsors in the review.	Page 20
Competing interests	26	Declare any competing interests of review authors.	Page 20
Availability of data, code and other materials	27	Report which of the following are publicly available and where they can be found: template data collection forms; data extracted from included studies; data used for all analyses; analytic code; any other materials used in the review.	Page 17

From: Page MJ, McKenzie JE, Bossuyt PM, Boutron I, Hoffmann TC, Mulrow CD, et al. The PRISMA 2020 statement: an updated guideline for reporting systematic reviews. BMJ 2021;372:n71. doi: 10.1136/bmj.n71

Supplementary Table 2: Search Strategy

Below is the search strategy for our review. Terms were combined with the Boolean operators AND (to link major concepts) and OR (to capture synonyms/variants). Searches were limited to articles published in English between 1 January 2010 and 1 October 2024.

Literature Database	Search Terms
PubMed	1. "surg* instrument*"[Title/Abstract] OR "surgical instruments"[MeSH] OR "surg* scene*"[Title/Abstract] OR "surg* tool*"[Title/Abstract] OR "anatom*"[Title/Abstract] OR "critical view of safety"[Title/Abstract]<br>2. "laparoscopy"[MeSH] OR "video-assisted surgery"[MeSH] OR "surg* video*"[Title/Abstract] OR "surg* imag*"[Title/Abstract] OR "laparoscop*"[Title/Abstract] OR "robot* surg*"[Title/Abstract] OR "minimally invasive"[Title/Abstract]<br>3. "Artificial Intelligence"[MeSH] OR "artificial intelligence"[Title/Abstract] OR "deep learning"[Title/Abstract] OR "machine learning"[Title/Abstract] OR "neural network*"[Title/Abstract] OR "segmentation"[Title/Abstract] OR "classification"[Title/Abstract] OR "computer vision"[Title/Abstract]<br>4. Final PubMed set: 1 AND 2 AND 3.
EMBASE	1. "surg* instrument*":ti,ab OR "surgical equipment":de OR "surg* scene*":ti,ab OR "surg* tool*":ti,ab OR "anatom*":ti,ab<br>2. "laparoscopy":de OR "laparoscopy":ti,ab OR "video-assisted surgery":de OR "surg* video*":ti,ab OR "surg* imag*":ti,ab OR "laparoscop*":ti,ab OR "robot* surg*":ti,ab OR "minimally invasive":ti,ab<br>3. "artificial intelligence":de OR "artificial intelligence":ti,ab OR "deep learning":ti,ab OR "machine learning":ti,ab OR "neural network*":ti,ab OR "computer vision":ti,ab OR "segmentation":ti,ab<br>4. Final EMBASE set: 1 AND 2 AND 3.
IEEE Xplore	1. "surg* instrument*" OR "surg* scene*" OR "surg* tool*" OR "anatom*" OR "critical view of safety"<br>2. "laparoscopy" OR "video-assisted surgery" OR "surg* video*" OR "surg* imag*" OR "laparoscop*" OR "robot* surg*" OR "minimally invasive"<br>3. "artificial intelligence" OR "deep learning" OR "machine learning" OR "neural network*" OR "segmentation" OR "classification" OR "computer vision"<br>4. Final IEEE Xplore: 1 AND 2 AND 3.
Google Scholar	"scene understanding" AND "surgical video" AND "artificial intelligence"
arXiv	1. "surgical instruments" OR "surgical scene" OR "surgical tool" OR anatomy OR "critical view of safety"<br>2. "laparoscopy" OR "video-assisted surgery" OR "surgical video" OR "surgical image" OR "robot surgery" OR "minimally invasive"<br>3. "artificial intelligence" OR "deep learning" OR "machine learning" OR "neural network" OR "segmentation" OR "classification" OR "computer vision"<br>4. Final Arxiv: 1 AND 2 AND 3.
medRxiv / bioRxiv	"surgical video" AND ("scene" OR "anatomy") AND "artificial intelligence"

Supplementary Table 3: Studies and study characteristics included in the systematic review and meta-analysis (1)

The following table presents an overview of all included studies, detailing key characteristics across four main categories: **Publication Characteristics, Medical Field, Data, and Model Characteristics**.

Publication Characteristics			Medical Field		Data								Model Characteristics		
**First author, year [reference]**	**Clarivate Journal Citation Reports Categories[a]**	**Authors background**	**Application field**	**Surgeries used**	**Proposed detection**	**Human (real) only or additional data**	**Data Origin**	**Dataset name(s)[b]**	**Annotation type**	**Annotation tool**	**Exclution of images containing artifacts**	**What was excluded**	**Model type**	**Temporal aspects**	**Training approach**
Acharya, 2022 [1]	Engineering Conference Paper	Computer Science	Gynecological Surgery	Laparoscopic Endometriosis	Organs/Tissue	Real	Public dataset(s)	GLENDA	Classification	unclear	Unclear	n/a	CNN	not reported	Supervised
Al Hajj, 2018 [2]	Radiology, Nuclear Medicine & Medical Imaging; Engineering, Biomedical; Computer Science, Interdisciplinary Applications; Computer Science, Artificial Intelligence	Interdisciplinary	Visceral / General Surgery	Laparoscopic cholecystectomy	Surgical Instruments	Real	Public dataset(s)	Cholec80	Classification	unclear	No	n/a	CNN	"(CNNs) whose outputs are fed to recurrent neural networks (RNNs) in order to take temporal relationships between events into account"	Supervised
Alapatt, 2021 [3]	Preprint	Interdisciplinary	Visceral / General Surgery	Laparoscopic cholecystectomy	Organs/Tissue, Surgical Instruments, Technical guidance	Real	Private dataset(s), Public dataset(s)	Endoscapes	Semantic	unclear	Unclear	n/a	CNN	Temporally Constrained Neural Network (TCNN)	Semi-supervised
Ali, 2022 [4]	Preprint	Computer Science	Visceral / General Surgery	Laparoscopic cholecystectomy	Surgical Instruments	Real	Public dataset(s)	m2cai-tl	Bounding box	unclear	Unclear	n/a	CNN	not reported	Semi-supervised
Alkhamaiseh, 2023 [5]	Surgery	Interdisciplinary	Visceral / General Surgery	Laparoscopic cholecystectomy	Organs/Tissue, Technical guidance	Real	Private dataset(s), Public dataset(s)	open source videos	Semantic	Fiji plugin (i.e., annotator)	Yes (clear criteria)	"14 videos were excluded from initial modelling because of poor image quality due to either bleeding or less visible or obscured landmarks, anatomical differences or misses, or procedures that do not follow the general standard of cholecystectomy."	CNN	not reported	Supervised
Alshirbaji, 2021 [6]	Engineering, Biomedical	Computer Science	Visceral / General Surgery	Laparoscopic cholecystectomy	Surgical Instruments	Real	Public dataset(s)	Cholec80	Classification	unclear	Unclear	n/a	CNN	STM-clip (sequence-to-one configuration)	Supervised
Alshirbaji, 2021 [7]	Engineering, Biomedical	Computer Science	Visceral / General Surgery	Laparoscopic cholecystectomy	Surgical Instruments	Real	Public dataset(s)	Cholec80, Cholec20	Classification	unclear	Unclear	n/a	CNN	not reported	Supervised
Alshirbaji, 2018 [8]	Engineering, Biomedical	Computer Science	Visceral / General Surgery	Laparoscopic cholecystectomy	Surgical Instruments	Real	Public dataset(s)	Cholec80	Classification	unclear	No	n/a	CNN	not reported	Supervised
Ángeles-Cerón, 2021 [9]	Engineering Conference Paper	Computer Science	Visceral / General Surgery	Laparoscopic Colectomy, Laparoscopic Rectal Resection, Laparoscopic Sigmoid Resection	Surgical Instruments	Real	Public dataset(s)	HeiCo	Semantic	unclear	Yes (clear criteria)	"996 frames with no visible instruments"	CNN	not reported	Supervised
Ángeles-Cerón, 2022 [10]	Radiology, Nuclear Medicine & Medical Imaging; Engineering, Biomedical; Computer Science, Interdisciplinary Applications; Computer Science, Artificial Intelligence	Computer Science	Visceral / General Surgery	Laparoscopic Colectomy, Laparoscopic Rectal Resection, Laparoscopic Sigmoid Resection	Surgical Instruments	Real	Public dataset(s)	ROBUST-MIS	Semantic	Understand.ai	Yes (clear criteria)	"17% empty frames(ef) on its trainingset. These frames do not have any visible instruments in them, and although we could have left them as negative examples for training,we remove them from the trainingset."	CNN	not reported	Supervised
Aoyama, 2024 [11]	Surgery	Interdisciplinary	Visceral / General Surgery	Laparoscopic Gastrectomy	Organs/Tissue, Technical guidance	Real	Private dataset(s)	n/a	Semantic	unclear	Unclear	n/a	CNN	not reported	Supervised
Arabian, 2022 [12]	Engineering, Biomedical	Computer Science	Visceral / General Surgery	Laparoscopic cholecystectomy	Surgical Instruments	Real	Public dataset(s)	Cholec80	Classification	unclear	Unclear	n/a	CNN	not reported	Supervised
Arabian, 2023 [13]	Chemistry, Analytical; Instruments & Instrumentation; Engineering, Electrical & Electronic	Computer Science	Visceral / General Surgery	Laparoscopic cholecystectomy	Surgical Instruments	Real, Also non-human data included	Public dataset(s)	Cholec80	Classification, Bounding box	unclear	Unclear	n/a	CNN	not reported	Supervised
Aspart, 2022 [14]	Surgery; Engineering, Biomedical; Radiology, Nuclear Medicine & Medical Imaging	Interdisciplinary	Visceral / General Surgery	Laparoscopic cholecystectomy	Surgical Instruments	Real	Private dataset(s)	n/a	Classification	unclear	Unclear	n/a	CNN	not reported	Supervised
Attia, 2017 [15]	Engineering Conference Paper	Computer Science	Visceral / General Surgery	Robotic Colorectal Surgery	Surgical Instruments	Real, Also non-human data included	Public dataset(s)	EndoVis15	Semantic	unclear	Unclear	n/a	CNN	RNN	Supervised
Ayobi, 2024 [16]	Preprint	Interdisciplinary	Urological Surgery	Robotic Prostatectomy	Surgical Instruments	Real	Private dataset(s)	GraSP	Semantic, Classification	Label-Studio, Toronto Annotation Suite	Unclear	n/a	CNN, TM	"we employ MViT as our video feature extractor to capture intricate details across various space-time scales by leveraging transformers."	Supervised
Bai, 2023 [17]	Preprint	Computer Science	Visceral / General Surgery	Laparoscopic cholecystectomy	Surgical Instruments	Real, Also non-human data included	Public dataset(s)	m2cai-t	Bounding box	unclear	Unclear	n/a	CNN	not reported	Supervised
Bakker, 2024 [18]	Urology & Nephrology	Interdisciplinary	Urological Surgery, Visceral / General Surgery	Robotic Prostatectomy, Robotic Rectal Resection	Organs/Tissue, Others	Real	Private dataset(s), Public dataset(s)	DSAD	Semantic	LabelMe, 3DSlicer	Yes (unclear criteria)	"if their intraoperative videos did not include the urethral dissection phase or if the image quality was too poor. And clearly visible and unobstructed parts of structures were annotated"	CNN	not reported	Supervised

Bamba, 2021 [19]	Surgery; Engineering, Biomedical; Radiology, Nuclear Medicine & Medical Imaging	Medicine	Visceral / General Surgery	Laparoscopic Colectomy, Laparoscopic Rectal Resection, Laparoscopic Hernia Repair, Laparoscopic Sigmoid Resection	Organs/Tissue, Surgical Instruments, Bleeding	Real	Private dataset(s)	n/a	Semantic	unclear	Unclear	n/a	CNN	not reported	Supervised
Bamba, 2021 [20]	Multidisciplinary Sciences	Interdisciplinary	Visceral / General Surgery	Laparoscopic Colectomy, Laparoscopic Rectal Resection, Laparoscopic Hernia Repair, Laparoscopic Sigmoid Resection	Surgical Instruments	Real	Private dataset(s)	n/a	Semantic	unclear	Unclear	n/a	CNN	not reported	Supervised
Ban, 2023 [21]	Preprint	Interdisciplinary	Visceral / General Surgery	Laparoscopic cholecystectomy	Organs/Tissue, Technical guidance	Real	Public dataset(s)	CholecT45	Classification	unclear	Unclear	n/a	CNN, TM	not reported	Supervised
Batić, 2024 [22]	Surgery; Engineering, Biomedical; Radiology, Nuclear Medicine & Medical Imaging	Computer Science	Visceral / General Surgery, Gynecological Surgery, Urological Surgery	Robotic Prostatectomy, Rectal Resection, Robotic Gastrectomy, Robotic Rectal Resection, Laparoscopic Gynecological Surgery, Laparoscopic cholecystectomy	Organs/Tissue, Surgical Instruments	Real	Public dataset(s)	ESAD, LapGyn4, Surgical Actions160, GLENDA, hsDB-instrument, HeiCo, PSI-AVA, DSAD, Cholec80, CholecT45, CholecSeg8k	Semantic	different	Yes (clear criteria)	"all synthetic images are excluded"	CNN, TM	not reported	Unsupervised, Supervised
Batić, 2023 [23]	Preprint	Computer Science	Visceral / General Surgery, Urological Surgery, Gynecological Surgery	Robotic Prostatectomy, Rectal Resection, Robotic Gastrectomy, Robotic Rectal Resection, Laparoscopic Gynecological Surgery, Laparoscopic cholecystectomy	Organs/Tissue, Surgical Instruments	Real	Public dataset(s)	ESAD, LapGyn4, Surgical Actions160, GLENDA, hsDB-instrument, HeiCo, PSI-AVA, DSAD, Cholec80, CholecT45, CholecSeg8k	Classification, No annotation	unclear	Unclear	n/a	CNN, TM	Multi-Stage Temporal Convolutional Network (MS-TCN) (for phase recognition)	Unsupervised, Supervised
Boonkong, 2022 [24]	Engineering Conference Paper	Interdisciplinary	Gynecological Surgery	Laparoscopic Gynecological Surgery	Surgical Instruments	Real	Private dataset(s)	n/a	Unclear	unclear	Unclear	n/a	CNN	not reported	Others/unclear
Brandenburg, 2023 [25]	Surgery	Interdisciplinary	Visceral / General Surgery	Robotic Esophagectomy	Organs/Tissue, Surgical Instruments, Bleeding, Others	Real	Private dataset(s)	n/a	Classification	CVAT	No	n/a	CNN	not reported	Active
Casella, 2021 [26]	Engineering Conference Paper	Computer Science	Urological Surgery	Robotic Partial Nephrectomy	Organs/Tissue	Real	Public dataset(s)	Nephrec9	Semantic	unclear	Yes (clear criteria)	"Frames with heavy motion blur (due to quick changes in camera position) and with largely occluded vessels were removed"	CNN	3D Fully-Convolutional Neural Network	Supervised
Chen, 2024 [27]	Engineering, Biomedical; Radiology, Nuclear Medicine & Medical Imaging; Engineering, Electrical & Electronic; Imaging Science & Photographic Technology; Computer Science, Interdisciplinary Applications	Interdisciplinary	Visceral / General Surgery	Laparoscopic cholecystectomy, Robotic Hepato-Pancreatic-Biliary Surgery	Organs/Tissue, Surgical Instruments	Real, Also non-human data included	Private dataset(s), Public dataset(s)	CholecSeg8k	No annotation, Semantic	unclear	Unclear	n/a	TM	not reported	Self-supervised
Chen, 2013 [28]	Engineering Conference Paper	Interdisciplinary	Others	unclear	Surgical Instruments	Real	Unclear	unclear	No annotation	n/a	Unclear	n/a	Others/Unclear (Spiking Neural Network (SNN))	not reported	Others/unclear
Chen, 2017 [29]	Engineering Conference Paper	Interdisciplinary	Visceral / General Surgery	unclear	Surgical Instruments	Real	Unclear	unclear	Bounding box	unclear	Unclear	n/a	CNN	"spatio-temporal context (STC) learning algorithm for tracking between video frames."	Supervised
Choi, 2017 [30]	Engineering Conference Paper	Computer Science	Visceral / General Surgery	Laparoscopic cholecystectomy	Surgical Instruments	Real	Public dataset(s)	m2cai-t	Bounding box, Classification	unclear	Unclear	n/a	CNN	not reported	Supervised
Ciaparrone, 2020 [31]	Engineering Conference Paper	Computer Science	Gynecological Surgery	unclear	Surgical Instruments	Real	Unclear	n/a	Semantic	VGG Image Annotator	No	"We chose to keep those noisy frames, as they can be useful to analyze if the models are able to generalize on unseen conditions and unfiltered noisy frames."	CNN	not reported	Supervised
Colleoni, 2024 [32]	Radiology, Nuclear Medicine & Medical Imaging; Engineering, Biomedical; Computer Science, Interdisciplinary Applications; Computer Science, Artificial Intelligence	Computer Science	Visceral / General Surgery, Urological Surgery	Laparoscopic cholecystectomy, Robotic Partial Nephrectomy, Robotic Prostatectomy	Organs/Tissue	Real, Synthetic	Public dataset(s), Private dataset(s)	CholecSeg8k	Semantic	unclear	Unclear	n/a	CNN, TM	not reported	Others/unclear
Colleoni, 2022 [33]	Engineering, Biomedical; Radiology, Nuclear Medicine & Medical Imaging; Engineering,	Computer Science	Urological Surgery	Robotic Prostatectomy	Surgical Instruments	Real, Synthetic, Also non-human data included	Public dataset(s),	RARP45	No annotation, Semantic	unclear	Unclear	n/a	CNN	not reported	Others/unclear

	Electrical & Electronic; Imaging Science & Photographic Technology; Computer Science, Interdisciplinary Applications						Private dataset(s)								
Daneshgar Rahbar, 2023 [34]	Imaging Science & Photographic Technology	Computer Science	Visceral / General Surgery, Others	Robotic Lobectomy, Robotic Prostatectomy	Surgical Instruments	Real, Also non-human data included	Public dataset(s)	US NLM	Semantic	unclear	Unclear	n/a	CNN	not reported	Supervised
Davila, 2023 [35]	Engineering Conference Paper	Computer Science	Visceral / General Surgery	Laparoscopic cholecystectomy	Surgical Instruments	Real, Also non-human data included	Public dataset(s)	Cholec80	Classification	unclear	Unclear	n/a	CNN	not reported	Supervised
De Backer, 2023 [36]	Urology & Nephrology	Interdisciplinary	Urological Surgery	Robotic Partial Nephrectomy	Surgical Instruments	Real	Private dataset(s)	n/a	Semantic	SuperAnnotate	Unclear	n/a	CNN	not reported	Supervised
den Boer, 2023 [37]	Surgery	Interdisciplinary	Visceral / General Surgery	Robotic Esophagectomy	Organs/Tissue	Real	Private dataset(s)	n/a	Semantic	LabelMe	Yes (clear criteria)	"Lymphatic or fatty tissue was excluded in the annotation of the anatomy."	CNN	not reported	Supervised
Derathé, 2020 [38]	Surgery; Engineering, Biomedical; Radiology, Nuclear Medicine & Medical Imaging	Interdisciplinary	Visceral / General Surgery	Laparoscopic Sleeve Gastrectomy	Surgical Instruments, Organs/Tissue, Technical guidance	Real	Private dataset(s)	n/a	Classification, Semantic	Surgery Workflow Toolbox, CamiTK	Unclear	n/a	Others/Unclear (Linear Discriminant Analysis (LDA); Support Vector Machine (SVM))	not reported	Supervised
Du, 2019 [39]	Radiology, Nuclear Medicine & Medical Imaging; Engineering, Biomedical; Computer Science, Interdisciplinary Applications; Computer Science, Artificial Intelligence	Computer Science	Visceral / General Surgery	Robotic Colorectal Surgery	Surgical Instruments	Real, Also non-human data included	Public dataset(s)	EndoVis Conventional Laparoscopic Instrument Dataset	Bounding box	unclear	No	n/a	Others/Unclear	not reported	Others/unclear
Endo, 2023 [40]	Surgery	Interdisciplinary	Visceral / General Surgery	Laparoscopic cholecystectomy	Organs/Tissue, Technical guidance	Real	Private dataset(s)	n/a	Semantic	unclear	Yes (clear criteria)	"We excluded LC cases with severe inflammation and abnormal biliary anatomy because it was difficult to annotate anatomical landmarks"	CNN	not reported	Supervised
Fernandez-Rodríguez, 2024 [41]	Preprint	Computer Science	Visceral / General Surgery	Laparoscopic cholecystectomy	Surgical Instruments	Real	Public dataset(s)	Cholec80, CholecSeg8k	Classification, Semantic	PixelAnnotaion Tool	Unclear	n/a	CNN	OpticalFlow	Supervised
Fuentes-Hurtado, 2019 [42]	Surgery; Engineering, Biomedical; Radiology, Nuclear Medicine & Medical Imaging	Computer Science	Visceral / General Surgery	Robotic Colorectal Surgery, Laparoscopic Sleeve Gastrectomy, Laparoscopic Gastric Bypass	Surgical Instruments	Real	Public dataset(s), Private dataset(s)	EndoVis15	Line, Semantic	unclear	Unclear	n/a	CNN	not reported	Supervised, Weakly-supervised
Fujinaga, 2023 [43]	Surgery	Interdisciplinary	Visceral / General Surgery	Laparoscopic cholecystectomy	Organs/Tissue, Technical guidance	Real	Private dataset(s)	n/a	Bounding box, Semantic	unclear	Yes (unclear criteria)	"with bad anatomical visibility"	CNN	not reported	Others/unclear
Ghamsarian, 2024 [44]	Surgery; Engineering, Biomedical; Radiology, Nuclear Medicine & Medical Imaging	Interdisciplinary	Gynecological Surgery	Laparoscopic Colorectal Surgery, Laparoscopic Endometriosis Surgery	Organs/Tissue	Real	Public dataset(s)	ENID	Semantic	Endoscopic Concept Annotation Tool	Yes (unclear criteria)		CNN	not reported	Supervised
Gitau, 2024 [45]	Engineering Conference Paper	Computer Science	Visceral / General Surgery	Laparoscopic cholecystectomy	Surgical Instruments, Others	Also non-human data included, Real	Public dataset(s)	Cholec80	Classification	unclear	Unclear	n/a	CNN	not reported	Supervised
Grammatikopoulou, 2023 [46]	Surgery; Engineering, Biomedical; Radiology, Nuclear Medicine & Medical Imaging	Computer Science	Urological Surgery, Visceral / General Surgery	Laparoscopic cholecystectomy, Robotic Partial Nephrectomy	Organs/Tissue, Surgical Instruments	Real	Private dataset(s), Public dataset(s)	CholecSeg8k	Semantic	PixelAnnotaion Tool	Unclear	n/a	CNN, TM	"spatio-temporal decoder based on the TCN model to augment any semantic segmentation backbone for Spatial temporal convolutional network (SP-TCN)"	Supervised
Guédon, 2021 [47]	Surgery	Interdisciplinary	Visceral / General Surgery, Gynecological Surgery	Laparoscopic cholecystectomy, Laparoscopic Hysterectomy	Surgical Instruments	Real	Private dataset(s)	n/a	Classification	NOUS application	Unclear	n/a	CNN	"temporal smoothing on the automatic recognition of the phases in order to avoid shifting between phases for only a few frames. For instance, as the network only used visual information, a few frames without instruments present in the frames may cause a wrong phase recognition. Therefore, a window of 15 to 30 frames was used for the smoothing on the automatic recognition of the phases."	Supervised
Hasan, 2021 [48]	Engineering Conference Paper	Computer Science	Visceral / General Surgery	Robotic Colorectal Surgery	Surgical Instruments	Real, Also non-human data included	Public dataset(s)	EndoVis15	Semantic	unclear	Unclear	n/a	CNN	not reported	Supervised
Huang, 2022 [49]	Robotics; Engineering, Biomedical	Interdisciplinary	Urological Surgery	Robotic Partial Nephrectomy, unclear	Surgical Instruments	Real	Public dataset(s)	n/a	No annotation, Semantic	unclear	Unclear	n/a	CNN	not reported	Unsupervised

Jalal, 2022 [50]	Engineering, Biomedical	Computer Science	Visceral / General Surgery	Laparoscopic cholecystectomy	Surgical Instruments	Real	Public dataset(s)	Cholec80	Bounding box, Classification	unclear	Yes (clear criteria)	"Images that have no tool or multiple instances of the same tool were excluded"	CNN	not reported	Weakly-supervised
Jalal, 2023 [51]	Chemistry, Analytical; Instruments & Instrumentation; Engineering, Electrical & Electronic	Computer Science	Visceral / General Surgery	Laparoscopic cholecystectomy	Surgical Instruments	Real	Public dataset(s)	Cholec80, Cholec80-Boxes	Classification, Bounding box	MATLAB Video Labeler toolbox	No	n/a	CNN	"The CNN-SE-MSF was combined with an LSTM network to model temporal dependencies along the video sequence"	Supervised
Jamal, 2024 [52]	Preprint	Computer Science	Urological Surgery, Gynecological Surgery, Visceral / General Surgery	Robotic Prostatectomy, Laparoscopic Hysterectomy, Laparoscopic cholecystectomy	Surgical Instruments	Real, Also non-human data included	Public dataset(s)	SAR-RARP50, AutoLaparo, LapI2I, CholecSeg8k	Semantic, Bounding box	unclear	Unclear	n/a	TM, CNN	not reported	Supervised
Jang, 2023 [53]	Engineering Conference Paper	Computer Science	Visceral / General Surgery	unclear	Organs/Tissue	Real	Unclear	unclear	No annotation, Bounding box	unclear	Unclear	n/a	CNN, TM	"forward and backward prediction in conjunction with a Siamese network"	Self-supervised
Jaspers, 2024 [54]	Engineering Conference Paper	Interdisciplinary	Visceral / General Surgery, Urological Surgery	Laparoscopic cholecystectomy, Robotic Esophagectomy, Robotic Prostatectomy, Robotic Rectal Resection, Laparoscopic Gynecological Surgery, Laparoscopic Gastric Bypass, Robotic Gastrectomy	Organs/Tissue	Real	Private dataset(s), Public dataset(s)	CholecSeg8k	Semantic	unclear	Unclear	n/a	CNN, TM	not reported	Supervised, Unsupervised
Jearanai, 2023 [55]	Surgery	Interdisciplinary	Visceral / General Surgery	Unclear	Organs/Tissue	Real	Private dataset(s)	n/a	Bounding box	Roboflow	Unclear	n/a	CNN	not reported	Supervised
Jha, 2021 [56]	Preprint	Computer Science	Visceral / General Surgery	Laparoscopic Colectomy, Laparoscopic Rectal Resection, Laparoscopic Hernia Repair, Laparoscopic Sigmoid Resection	Surgical Instruments	Real	Public dataset(s)	ROBUST-MIS	Semantic	understand.ai	Unclear	n/a	CNN	not reported	Supervised
Jin, 2020 [57]	Radiology, Nuclear Medicine & Medical Imaging; Engineering, Biomedical; Computer Science, Interdisciplinary Applications; Computer Science, Artificial Intelligence	Computer Science	Visceral / General Surgery	Laparoscopic cholecystectomy	Surgical Instruments	Real	Public dataset(s)	Cholec80	Classification	unclear	No	n/a	CNN	RNN (for phase recognition)	Supervised
Jin, 2018 [58]	Preprint	Computer Science	Visceral / General Surgery	Laparoscopic cholecystectomy	Surgical Instruments	Real	Public dataset(s)	m2cai-tl	Bounding box, Classification	unclear	Unclear	n/a	CNN	not reported	Supervised
Kamrul Hasan, 2021 [59]	Radiology, Nuclear Medicine & Medical Imaging; Engineering, Biomedical; Computer Science, Interdisciplinary Applications; Computer Science, Artificial Intelligence	Computer Science	Gynecological Surgery	Laparoscopic Hysterectomy	Surgical Instruments	Real, Also non-human data included	Private dataset(s)	n/a	Semantic, Line, Dot, Classification	ImageJ	Unclear	n/a	CNN	not reported	Supervised
Kanakatte, 2020 [60]	Engineering Conference Paper	Computer Science	Visceral / General Surgery	Laparoscopic cholecystectomy	Surgical Instruments	Real	Public dataset(s)	Cholec80	Semantic	LabelMe	Unclear	n/a	CNN	Spatio-temporal deep network (ST-LSTM)	Supervised
Kawamura, 2023 [61]	Surgery	Interdisciplinary	Visceral / General Surgery	Laparoscopic cholecystectomy	Organs/Tissue, Technical guidance	Real	Private dataset(s)	n/a	Classification	own	Yes (clear criteria)	"excluded patients with severe cholecystitis that did not meet the CVS requirements due to the inability to identify arteries and those forced to proceed to bailout surgery or laparotomy"	CNN	not reported	Supervised
Khalid, 2023 [62]	Surgery	Interdisciplinary	Visceral / General Surgery	Laparoscopic cholecystectomy	Organs/Tissue, Technical guidance	Real	Private dataset(s), Unclear	n/a	Semantic	GoNoGoNet	Yes (clear criteria)	"only included if all tool-tissue interactions were deemed to have occurred within the surgeon-assessed Go zone."	CNN	not reported	Others/unclear
Khalid, 2023 [63]	Preprint	Computer Science	Visceral / General Surgery	Laparoscopic cholecystectomy, Laparoscopic Appendectomy, Laparoscopic Hernia Repair	Surgical Instruments	Real	Private dataset(s)	unclear	No annotation, Semantic	unclear	Unclear	n/a	Others/Unclear (GNN)	"radiance fields assisted 3D feature extraction"	Self-supervised, Supervised
Kim, 2024 [64]	Engineering Conference Paper	Computer Science	Visceral / General Surgery	Laparoscopic cholecystectomy	Surgical Instruments	Real	Public dataset(s)	m2cai-tl	Bounding box	unclear	Unclear	n/a	CNN	not reported	Supervised
Kinoshita, 2024 [65]	Surgery	Interdisciplinary	Visceral / General Surgery	Robotic Rectal Resection, Laparoscopic Rectal Resection	Organs/Tissue	Real	Private dataset(s)	n/a	Semantic	unclear	Unclear	n/a	CNN	not reported	Supervised
Kitaguchi, 2023 [66]	Surgery	Interdisciplinary	Visceral / General Surgery	Laparoscopic Colorectal Resection	Organs/Tissue	Real	Private dataset(s)	n/a	Semantic	unclear	Yes (clear criteria)	"he study cohort did not include patients with a high BMI or severe intra-abdominal adhesions due to previous surgery"	CNN	not reported	Supervised
Kitaguchi, 2022 [67]	Medicine, General & Internal	Interdisciplinary	Visceral / General Surgery	Laparoscopic Colorectal Resection	Surgical Instruments	Real	Private dataset(s)	n/a	Semantic	unclear	Unclear	n/a	CNN	not reported	Supervised
Kitaguchi, 2022 [68]	Multidisciplinary Sciences	Interdisciplinary	Visceral / General Surgery	Laparoscopic Colorectal	Surgical Instruments	Real	Private dataset(s)	n/a	Semantic	unclear	Yes (clear criteria)	"out-of-focus images and/or images with mist were excluded"	CNN	not reported	Supervised

				Resection, Laparoscopic Distal Gastrectomy, Laparoscopic cholecystectomy, Laparoscopic Partial Hepatectomy											
Kletz, 2019 [69]	Engineering Conference Paper	Interdisciplinary	Gynecological Surgery	Laparoscopic Gynecological Surgery	Surgical Instruments	Real	Private dataset(s)	n/a	Semantic	unclear	Unclear	n/a	CNN	not reported	Supervised
Kolbinger, 2023 [70]	Surgery	Interdisciplinary	Visceral / General Surgery	Robotic Rectal Resection	Organs/Tissue, Technical guidance	Real	Public dataset(s)	DSAD	Semantic, Bounding box	3DSlicer, CVAT	No	n/a	CNN	not reported	Supervised
Kolbinger, 2024 [71]	Oncology; Surgery	Interdisciplinary	Visceral / General Surgery	Robotic Rectal Resection	Organs/Tissue	Real	Private dataset(s)	n/a	Semantic	3DSlicer	No	n/a	CNN	not reported	Supervised
Kolbinger, 2024 [72]	Engineering Conference Paper	Interdisciplinary	Visceral / General Surgery	Robotic Rectal Resection	Organs/Tissue	Real	Public dataset(s)	DSAD	Semantic	3DSlicer	No	n/a	TM	not reported	Supervised
Kondo, 2021 [73]	Engineering, Biomedical	Computer Science	Visceral / General Surgery	Laparoscopic cholecystectomy	Surgical Instruments	Real	Public dataset(s)	Cholec80	Classification	unclear	No	n/a	TM, CNN	"Transformer architecture to analyse inter-frame correlation"	Supervised
Konduri, 2024 [74]	Engineering, Biomedical	Computer Science	Visceral / General Surgery, Gynecological Surgery	Laparoscopic cholecystectomy, Laparoscopic Gynecological Surgery, Laparoscopic Colectomy, Laparoscopic Rectal Resection, Laparoscopic Sigmoid Resection	Surgical Instruments	Real	Public dataset(s)	CholecSeg8k, LapGyn4, HeiCo	Classification	PixelAnnotaion Tool	Unclear	n/a	CNN	not reported	Supervised
Kong, 2021 [75]	Surgery; Engineering, Biomedical; Radiology, Nuclear Medicine & Medical Imaging	Computer Science	Gynecological Surgery	Robotic Hysterectomy	Surgical Instruments	Real, Also non-human data included	Private dataset(s)	n/a	Semantic	Labelme	Unclear	n/a	CNN	not reported	Supervised
Kumazu, 2021 [76]	Multidisciplinary Sciences	Interdisciplinary	Visceral / General Surgery	Robotic Gastrectomy	Organs/Tissue, Technical guidance	Real	Private dataset(s)	n/a	Semantic	unclear	Unclear	n/a	CNN	not reported	Supervised
Kumazu, 2025 [77]	Multidisciplinary Sciences	Interdisciplinary	Visceral / General Surgery	Robotic Gastrectomy, Laparoscopic Gastrectomy, Laparoscopic Colorectal Surgery, Laparoscopic Hernia Repair	Organs/Tissue, Technical guidance	Real	Private dataset(s)	n/a	Semantic	unclear	Yes (clear criteria)	"Conversely, we excluded images in which the LCT was not clearly visible due to bleeding, smoking, or artifacts, and in which the LCT had degenerated due to inflammation or prior treatment. Additionally, we excluded evaluation scenes in which the surgical procedure was not progressing smoothly."	CNN	not reported	Supervised
Labrunie, 2022 [78]	Surgery; Engineering, Biomedical; Radiology, Nuclear Medicine & Medical Imaging	Interdisciplinary	Visceral / General Surgery	Laparoscopic Liver Resection	Organs/Tissue	Real	Private dataset(s)	n/a	Semantic	unclear	Unclear	n/a	CNN	not reported	Supervised
Lam, 2022 [79]	Robotics; Engineering, Biomedical	Computer Science	Visceral / General Surgery	Laparoscopic Gastric Band Insertion	Surgical Instruments	Real	Private dataset(s)	n/a	Semantic	VGG Image annotator	Unclear	n/a	CNN	Markov-chain	Supervised
Laplante, 2022 [80]	Surgery	Interdisciplinary	Visceral / General Surgery	Laparoscopic cholecystectomy	Organs/Tissue, Technical guidance	Real	Private dataset(s)	n/a	Semantic	Think Like A Surgeon	Yes (clear criteria)	"Exclusion criteria included: 1) top-down approach LC, and 2) poor visualization of the hepatocystic triangle whereby the entire triangle was never in full view"	CNN	not reported	Supervised
Lavanchy, 2021 [81]	Multidisciplinary Sciences	Interdisciplinary	Visceral / General Surgery	Laparoscopic cholecystectomy	Surgical Instruments	Real	Private dataset(s)	n/a	Bounding box, Classification	unclear	Unclear	n/a	CNN	not reported	Supervised
Le, 2023 [82]	Engineering Conference Paper	Computer Science	Visceral / General Surgery	Laparoscopic cholecystectomy	Surgical Instruments	Real	Public dataset(s)	m2cai-tl	Bounding box	unclear	No	n/a	CNN	not reported	Supervised
Lee, 2024 [83]	Computer Science, Interdisciplinary Applications; Computer Science, Information Systems; Mathematical & Computational Biology; Medical Informatics	Computer Science	Visceral / General Surgery	Laparoscopic cholecystectomy, Laparoscopic Colectomy, Laparoscopic Rectal Resection, Laparoscopic Sigmoid Resection	Organs/Tissue, Surgical Instruments	Real	Public dataset(s)	CholecSeg8K, ROBUST-MIS	Semantic, Bounding box	PixelAnnotaion Tool, understand.ai	Unclear	n/a	CNN, Others/Unclear (Generative Model (DIPO))	not reported	Supervised
Leifman, 2024 [84]	Computer Science; Artificial Intelligence; Computer Science Applications	Interdisciplinary	Visceral / General Surgery	Laparoscopic cholecystectomy	Organs/Tissue, Technical guidance	Real	Private dataset(s), Public dataset(s)	Cholec80	Classification, Semantic	unclear	Yes (unclear criteria)	"Eligibility criteria were laparoscopic cholecystectomy for biliary colic or acute and chronic cholecystitis in patients 18 years of age or older."	CNN	"Inspired by the success of the above methods, we propose an approach similar in spirit to DeepCVS, but we add the temporal aggregation stage, and our approach can work in real-time settingsapproach similar in spirit to DeepCVS, but we add the temporal aggregation stage, and our approach can work in real-time settings"	Supervised
Leifmann, 2022 [85]	Engineering Conference Paper	Computer Science	Visceral / General Surgery	Laparoscopic Colectomy, Laparoscopic Rectal Resection, Laparoscopic	Surgical Instruments	Synthetic, Real, Also non-human data included	Public dataset(s)	Robotool	Bounding box	Understand.ai	Unclear	n/a	CNN	not reported	Supervised

				Sigmoid Resection, Unclear											
Li, 2025 [86]	Preprint	Interdisciplinary	Urological Surgery, Gynecological Surgery, Visceral / General Surgery	Laparoscopic cholecystectomy, Unclear	Organs/Tissue, Surgical Instruments, Bleeding	Real	Public dataset(s)	CholecSeg8K	Classification, No annotation	unclear	Unclear	n/a	TM	Spatial-temporal fusion layer	Weakly-supervised
Li, 2023 [87]	Engineering Conference Paper	Interdisciplinary	Visceral / General Surgery	Laparoscopic cholecystectomy	Organs/Tissue, Technical guidance	Real	Public dataset(s)	CholecT50, Cholec80, open source videos	Semantic	PixelAnnotaion Tool	Unclear	n/a	TM	not reported	Supervised
Liao, 2024 [88]	Preprint	Interdisciplinary	Visceral / General Surgery	Laparoscopic cholecystectomy	Surgical Instruments	Real	Public dataset(s)	Cholec80, CholecSeg8k	Semantic	PixelAnnotaion Tool	Unclear	n/a	CNN	"Both modules utilize a spatio-temporal class activation map (ST-CAM) and share input from ViT [4] encoded frames and video-level class labels as the supervision signal."	Weakly-supervised
Lin, 2024 [89]	Engineering, Biomedical; Radiology, Nuclear Medicine & Medical Imaging; Engineering, Electrical & Electronic; Imaging Science & Photographic Technology; Computer Science, Interdisciplinary Applications	Interdisciplinary	Visceral / General Surgery	Laparoscopic cholecystectomy	Organs/Tissue, Surgical Instruments	Real	Public dataset(s)	CholecQ	Bounding box, Classification	unclear	Unclear	n/a	CNN	"Global Average Pooling Layer and Long Short-Term Memory (LSTM) Layer to obtain the snippet context features. These obtained snippet context features include temporal information about the key frame and reference frames."	Supervised
Lin, 2021 [90]	Robotics	Interdisciplinary	Visceral / General Surgery	Laparoscopic Proctocolectomy	Surgical Instruments	Real, Synthetic	Public dataset(s)	ROBUST-MIS	Semantic	Understand.ai	Yes (unclear criteria)	"As an initial exploration, we evaluated our method only on the proctocolectomy dataset for the binary segmentation task."	CNN	"Multi-frame Feature Aggregation (MFFA) module that aggregates features both temporally and spatially for segmentation"	Others/unclear
Liu, 2022 [91]	Surgery	Interdisciplinary	Visceral / General Surgery	Laparoscopic cholecystectomy	Organs/Tissue, Technical guidance	Real	Private dataset(s)	n/a	Bounding box	unclear	Yes (clear criteria)	"excluding videos that were incomplete (n = 1), pixels or format incompatible (n = 17), or gallbladder gangrene (n = 1)"	CNN	not reported	Supervised
Liu, 2024 [92]	Engineering, Biomedical; Radiology, Nuclear Medicine & Medical Imaging; Engineering, Electrical & Electronic; Imaging Science & Photographic Technology; Computer Science, Interdisciplinary Applications	Computer Science	Visceral / General Surgery	Robotic Rectal Resection	Surgical Instruments	Real	Private dataset(s)	n/a	Semantic	unclear	Unclear	n/a	CNN, TM	not reported	Supervised
Liu, 2022 [93]	Robotics; Engineering, Biomedical	Interdisciplinary	Visceral / General Surgery	Laparoscopic cholecystectomy	Surgical Instruments	Real, Also non-human data included	Public dataset(s)	Cholec80-locations	Bounding box	unclear	Unclear	n/a	CNN	"Given that the input image is a continuous video frame, which is time-dependent, RNN was added to the CNN framework. On the other hand, since the input multi-channel color image is a tensor, the convolutional LSTM structure was used to extract the image’s temporal information. At this stage, we formed the DSCNet-CLSTM network, which could utilize the temporal and spatial context information of laparoscopic video frames"	Supervised
Lou, 2023 [94]	Engineering, Biomedical; Radiology, Nuclear Medicine & Medical Imaging; Engineering, Electrical & Electronic; Imaging Science & Photographic Technology; Computer Science, Interdisciplinary Applications	Interdisciplinary	Gynecological Surgery	Unclear	Surgical Instruments	Real	Public dataset(s)	ART-NET	Semantic	unclear	Unclear	n/a	CNN	not reported	Semi-supervised
Loukas, 2020 [95]	Surgery	Computer Science	Visceral / General Surgery	Laparoscopic cholecystectomy	Organs/Tissue, Others	Real	Public dataset(s)	m2cai-w	Classification	unclear	Unclear	n/a	Others/Unclear (Bayesian gaussian mixture models (VBGMM))	not reported	Others/unclear, Unsupervised
Loza, 2024 [96]	Engineering, Biomedical	Computer Science	Visceral / General Surgery	Laparoscopic cholecystectomy	Surgical Instruments	Real	Public dataset(s)	m2cai-tl	Bounding box	unclear	Unclear	n/a	TM	not reported	Supervised
Maack, 2024 [97]	Engineering Conference Paper	Computer Science	Visceral / General Surgery	Robotic Rectal Resection	Organs/Tissue	Real	Public dataset(s)	DSAD	Semantic	3DSlicer	No	n/a	CNN, TM	not reported	Supervised

Madad Zadeh, 2020 [98]	Surgery	Interdisciplinary	Gynecological Surgery	Laparoscopic Hysterectomy	Organs/Tissue, Surgical Instruments	Real	Public dataset(s), Private dataset(s)	SurgAI	Semantic	Supervisely	Unclear	n/a	CNN	not reported	Supervised
Madani, 2022 [99]	Surgery	Interdisciplinary	Visceral / General Surgery	Laparoscopic cholecystectomy	Organs/Tissue, Technical guidance	Real	Public dataset(s)	Cholec80, m2cai-w	Semantic	Think Like A Surgeon	Yes (unclear criteria)	"Frames wherein the camera was outside & 2900 frames were extracted, 273 of which were excluded"	CNN	not reported	Supervised
Maqbool, 2020 [100]	Preprint	Computer Science	Visceral / General Surgery	Laparoscopic cholecystectomy	Organs/Tissue, Surgical Instruments, Bleeding	Real	Public dataset(s)	m2cai-t	Semantic	MatLab	Unclear	n/a	CNN	not reported	Supervised
Marullo, 2023 [101]	Pharmacology & Pharmacy	Interdisciplinary	Urological Surgery	Robotic Prostatectomy	Surgical Instruments, Bleeding	Real	Private dataset(s)	n/a	Semantic, Classification	unclear	Yes (clear criteria)	"due to poor resolution, or the absence of surgical equipment were dataset, which comprised 318 images. The dataset was then divided into train, validation, removed"	CNN	not reported	Supervised
Mascagni, 2022 [102]	Surgery	Interdisciplinary	Visceral / General Surgery	Laparoscopic cholecystectomy	Organs/Tissue, Technical guidance	Real	Private dataset(s)	n/a	Semantic	Pixel Annotation Tool	Yes (unclear criteria)	"After manual screening, 2854 images of CVS were annotated and included in the CVS dataset."	CNN	not reported	Supervised
Matsumoto, 2024 [103]	Multidisciplinary Sciences	Interdisciplinary	Visceral / General Surgery	Laparoscopic Gastrectomy	Surgical Instruments	Real	Private dataset(s)	n/a	Semantic	unclear	Unclear	n/a	CNN	not reported	Supervised
Mehta, 2024 [104]	Surgery; Engineering, Biomedical; Radiology, Nuclear Medicine & Medical Imaging	Computer Science	Visceral / General Surgery	Laparoscopic cholecystectomy	Organs/Tissue, Technical guidance	Real	Unclear	n/a	Semantic	unclear	Unclear	n/a	TM	not reported	Supervised
Mishra, 2017 [105]	Engineering Conference Paper	Computer Science	Visceral / General Surgery	Laparoscopic cholecystectomy	Surgical Instruments	Real	Public dataset(s)	m2cai-t	Classification	unclear	Unclear	n/a	CNN	"long short-term memory (LSTM) on the extracted spatial features of the video sequence to capture the temporal connectionism across deep residual visual features and thereby increase the accuracy in prediction."	Supervised
Murali, 2024 [106]	Preprint	Computer Science	Visceral / General Surgery	Laparoscopic cholecystectomy	Organs/Tissue, Surgical Instruments, Technical guidance	Real	Public dataset(s)	Endoscapes-Seg50	Semantic	unclear	Unclear	n/a	CNN, TM	CycleSelect	Supervised
Murali, 2023 [107]	Preprint	Interdisciplinary	Visceral / General Surgery	Laparoscopic cholecystectomy	Organs/Tissue, Technical guidance	Real	Public dataset(s)	Endoscapes+	Classification, Semantic, Bounding box	unclear	Unclear	n/a	CNN, TM	"latent spatiotemporal graph representations of entire surgical videos, with each node representing a surgical tool or anatomical structure and edges representing relationships between nodes across space and time."	Supervised
Murali, 2022 [108]	Engineering, Biomedical; Radiology, Nuclear Medicine & Medical Imaging; Engineering, Electrical & Electronic; Imaging Science & Photographic Technology; Computer Science, Interdisciplinary Applications	Interdisciplinary	Visceral / General Surgery	Laparoscopic cholecystectomy	Organs/Tissue, Technical guidance, Surgical Instruments	Real	Public dataset(s)	DeepCVS, Endoscapes+	Classification, Semantic, Bounding box	unclear	Unclear	n/a	CNN, TM	not reported	Semi-supervised
Myo, 2024 [109]	Telecommunications; Computer Science, Information Systems; Engineering, Electrical & Electronic	Interdisciplinary	Visceral / General Surgery	Laparoscopic Colectomy, Laparoscopic Rectal Resection, Laparoscopic Sigmoid Resection	Surgical Instruments	Real	Public dataset(s)	ROBUST-MIS	Semantic	Roboflow online annotation tool	No	n/a	CNN	"Modified Y+BT algorithm consists of YOLOv8 as the core segmentation and ByteTrack as an adapted module. Two confidence thresholds (high and low) have to be defined for ByteTrack in order to take advantage of having 2 associations between the current frame and the previous frame"	Supervised
Nakanuma, 2023 [110]	Surgery	Interdisciplinary	Visceral / General Surgery	Laparoscopic cholecystectomy	Organs/Tissue, Technical guidance	Real	Private dataset(s)	n/a	Semantic	unclear	Unclear	n/a	CNN	not reported	Supervised
Namazi, 2022 [111]	Surgery	Interdisciplinary	Visceral / General Surgery	Laparoscopic cholecystectomy	Surgical Instruments	Real	Public dataset(s)	m2cai-td, Cholec80	Classification	unclear	Unclear	n/a	CNN	not reported	Supervised
Nema , 2023 [112]	Surgery	Computer Science	Visceral / General Surgery	Robotic Colorectal Surgery	Surgical Instruments	Real, Also non-human data included, Synthetic	Public dataset(s)	EndoVis15	Semantic	unclear	No	n/a	CNN, Others/Unclear (Generative Adversarial Network (GAN))	not reported	Supervised
Nwoye, 2024 [113]	Preprint	Computer Science	Visceral / General Surgery	Laparoscopic cholecystectomy	Surgical Instruments	Real	Public dataset(s)	CholecTrack20	Bounding box	unclear	Unclear	n/a	CNN	Multi-Class Multi-Object Tracking (MC-MOT)	Weakly-supervised, Self-supervised, Supervised

Nwoye, 2019 [114]	Surgery; Engineering, Biomedical; Radiology, Nuclear Medicine & Medical Imaging	Computer Science	Visceral / General Surgery	Laparoscopic cholecystectomy	Surgical Instruments	Real	Public dataset(s)	Cholec80	Classification, Bounding box	unclear	Unclear	n/a	CNN	"As temporal model, we propose to use a recurrent neural network (RNN), with the aim to determine the current position of each tool from the input feature map along with information from prior images captured in RNN's state."	Weakly-supervised
Oh, 2024 [115]	Multidisciplinary Sciences	Interdisciplinary	Visceral / General Surgery	Laparoscopic Hepatectomy	Organs/Tissue	Real	Private dataset(s)	n/a	Semantic	CVAT	Yes (clear criteria)	"Frames with obscured fields due to smoke, completely obscured biliary structures by surgical instruments, or camera positioned outside the surgical field were excluded."	CNN	not reported	Supervised
Owen, 2022 [116]	Surgery; Engineering, Biomedical; Radiology, Nuclear Medicine & Medical Imaging	Computer Science	Visceral / General Surgery	Laparoscopic cholecystectomy	Organs/Tissue, Technical guidance	Real	Private dataset(s)	unclear	Semantic	unclear	Unclear	n/a	CNN	not reported	Semi-supervised
Ozbulak, 2025 [117]	Preprint	Interdisciplinary	Visceral / General Surgery	Laparoscopic cholecystectomy	Organs/Tissue, Surgical Instruments	Real	Public dataset(s)	CholecSeg8k	Semantic	PixelAnnotaion Tool	Unclear	n/a	TM	not reported	Others/unclear
Pan, 2023 [118]	Surgery; Engineering, Biomedical; Radiology, Nuclear Medicine & Medical Imaging	Interdisciplinary	Visceral / General Surgery	Laparoscopic cholecystectomy, Laparoscopic Gastrectomy	Surgical Instruments	Real, Also non-human data included	Public dataset(s), Private dataset(s)	m2cai-tl	Bounding box	VoTT, LabelMe	Unclear	n/a	CNN	not reported	Supervised
Penza, 2018 [119]	Radiology, Nuclear Medicine & Medical Imaging; Engineering, Biomedical; Computer Science, Interdisciplinary Applications; Computer Science, Artificial Intelligence	Computer Science	Visceral / General Surgery	Unclear	Organs/Tissue	Real, Also non-human data included	Private dataset(s)	n/a	Semantic, Classification	unclear	Unclear	n/a	Others/Unclear (Kanade-Lucas-Tomasi (KLT) Tracker and SURF (Speeded-Up Robust Features))	Optical Flow	Others/unclear
Pradeep, 2022 [120]	Engineering Conference Paper	Computer Science	Visceral / General Surgery	Laparoscopic cholecystectomy	Surgical Instruments	Real	Public dataset(s)	m2cai-tl	Bounding box	unclear	Unclear	n/a	CNN	"Surgical Phase Aware Encoder-Decoder Architecture for Surgical Tool Presence Detection and Tool Localization"	Supervised
Prokopetc, 2015 [121]	Engineering Conference Paper	Computer Science	Gynecological Surgery	Laparoscopic Gynecological Surgery	Organs/Tissue	Real	Unclear	unclear	Bounding box	unclear	Unclear	n/a	Others/Unclear (DPM Model)	not reported	Supervised
Protserov, 2024 [122]	Health Care Sciences & Services; Medical Informatics	Interdisciplinary	Visceral / General Surgery	Laparoscopic cholecystectomy	Organs/Tissue, Technical guidance	Real	Public dataset(s), Private dataset(s)	open source videos	Semantic	unclear	Unclear	n/a	CNN, TM	not reported	Supervised
Rahbar, 2020 [123]	Surgery	Computer Science	Urological Surgery, Others	Robotic Lobectomy, Robotic Thoracoscopy, Robotic Prostectomy	Bleeding	Real	Public dataset(s)	US NLM	Dot	Adobe After Effects 2020	No	n/a	Others/Unclear	"For real-time detection of arterial-type bleeding, the algorithm tracks the change in the entropy map distribution. That is, it tracks the local entropy maps from the prior step for each frame over a period of time. The local entropy map localizes the regions with a high degree of homogeneity. For the sake of quantification, we binarize the entropy map and compute the number of non-zeropixels as an indicator of uniformity of the different regions of the video's content with respect to time."	Supervised
Rahbar, 2024 [124]	Health Care Sciences & Services	Computer Science	Visceral / General Surgery	Laparoscopic cholecystectomy, Robotic Lobectomy, Robotic Thoracoscopy, Robotic Prostectomy	Surgical Instruments	Real, Also non-human data included	Public dataset(s)	US NLM, ROBUST-MIS, Endoscapes	Semantic	unclear	Unclear	n/a	CNN	not reported	Supervised
Raja, 2024 [125]	Engineering Conference Paper	Computer Science	Visceral / General Surgery	Laparoscopic cholecystectomy	Surgical Instruments	Real	Public dataset(s)	m2cai-tl	Bounding box	unclear	Unclear	n/a	CNN	not reported	Supervised
Ryu, 2024 [126]	Surgery	Interdisciplinary	Visceral / General Surgery	Laparoscopic Hemicolectomy	Organs/Tissue	Real	Private dataset(s)	n/a	Semantic	CVAT	Unclear	n/a	CNN	not reported	Supervised
Sahu, 2017 [127]	Surgery; Engineering, Biomedical; Radiology, Nuclear Medicine & Medical Imaging	Computer Science	Visceral / General Surgery	Laparoscopic cholecystectomy	Surgical Instruments	Real	Public dataset(s)	m2cai-t	Classification	unclear	No	n/a	CNN	"Temporal smoothing: It assumes that each tool transition within the endoscopic videos is	Supervised

														smooth and takes previous frame detections into account in a weighted scheme. A window of five frames (including current and four previous frames) with normalized linear weights determines the current output detection."	
Samuel, 2021 [128]	Robotics	Computer Science	Visceral / General Surgery	Laparoscopic cholecystectomy	Surgical Instruments	Real, Also non-human data included	Public dataset(s)	Cholec80	Classification	unclear	Unclear	n/a	CNN	not reported	Unsupervised
Sanchez-Matilla, 2022 [129]	Surgery; Engineering, Biomedical; Radiology, Nuclear Medicine & Medical Imaging	Computer Science	Visceral / General Surgery	Laparoscopic cholecystectomy	Organs/Tissue, Surgical Instruments	Real	Public dataset(s)	CholecSeg8k	Semantic	PixelAnnotaion Tool	Unclear	n/a	CNN	Multi-Stage TCN	Supervised
Satyanaik, 2024 [130]	Surgery; Engineering, Biomedical; Radiology, Nuclear Medicine & Medical Imaging	Computer Science	Visceral / General Surgery	Laparoscopic cholecystectomy	Organs/Tissue, Technical guidance	Real	Public dataset(s)	Endoscapes2023, Endoscapes-WC70	Semantic	unclear	Unclear	n/a	CNN	not reported	Supervised, Others/unclear, Semi-supervised
Satyanaik, 2024 [131]	Engineering Conference Paper	Interdisciplinary	Visceral / General Surgery	Laparoscopic cholecystectomy	Organs/Tissue, Technical guidance	Real	Public dataset(s)	Endoscapes2023, Endoscapes-WC70	Semantic, Classification	unclear	Unclear	n/a	CNN	not reported	Semi-supervised
Seenivasan, 2023 [132]	Preprint	Computer Science	Visceral / General Surgery	Laparoscopic cholecystectomy, Robotic Prostatectomy	Surgical Instruments	Real	Public dataset(s)	Cholec80, PSI-AVA	Classification	unclear	Unclear	n/a	CNN, TM	not reported	Supervised
Sengun, 2023 [133]	Surgery	Interdisciplinary	Visceral / General Surgery	Laparoscopic Adrenalectomy	Organs/Tissue	Real	Private dataset(s)	n/a	Semantic	CVAT	Yes (unclear criteria)	"of 66 videos, 40 met the inclusion criteria"	CNN	not reported	Supervised
Sengun, 2024 [134]	Surgery	Interdisciplinary	Visceral / General Surgery	Laparoscopic Adrenalectomy	Organs/Tissue	Real	Private dataset(s)	n/a	Semantic	LabelBox	No	n/a	CNN, TM	not reported	Supervised
Shen, 2023 [135]	Engineering, Biomedical; Radiology, Nuclear Medicine & Medical Imaging; Engineering, Electrical & Electronic; Imaging Science & Photographic Technology; Computer Science, Interdisciplinary Applications	Computer Science	Visceral / General Surgery	Unclear	Surgical Instruments	Real, Also non-human data included	Private dataset(s)	n/a	Semantic	unclear	Unclear	n/a	CNN, TM	not reported	Supervised
Sheng, 2024 [136]	Preprint	Interdisciplinary	Visceral / General Surgery	Laparoscopic cholecystectomy	Surgical Instruments, Organs/Tissue	Real, Also non-human data included, Synthetic	Public dataset(s)	CholecSeg8k	Semantic, No annotation	PixelAnnotaion Tool	Unclear	n/a	TM	not reported	Unsupervised
Shi, 2020 [137]	Telecommunications; Computer Science, Information Systems; Engineering, Electrical & Electronic	Interdisciplinary	Visceral / General Surgery	Laparoscopic cholecystectomy	Surgical Instruments	Real	Public dataset(s)	Cholec80	Bounding box, Classification	unclear	Unclear	n/a	CNN	not reported	Supervised
Shimgekar, 2021 [138]	Engineering Conference Paper	Computer Science	Visceral / General Surgery	Unclear	Surgical Instruments	Real	Private dataset(s)	n/a	Semantic	LabelMe	Unclear	n/a	CNN	not reported	Supervised
Silva, 2022 [139]	Engineering Conference Paper	Computer Science	Visceral / General Surgery	Laparoscopic cholecystectomy	Organs/Tissue, Surgical Instruments	Real	Public dataset(s)	CholecSeg8k	Semantic	PixelAnnotaion Tool	Unclear	n/a	CNN, TM	not reported	Supervised
Smithmaitrie, 2024 [140]	Multidisciplinary Sciences	Interdisciplinary	Visceral / General Surgery	Unclear	Organs/Tissue	Real	Private dataset(s)	n/a	Bounding box	LabelImg	Yes (clear criteria)	"no cases involving cancerous lesions were included"	CNN	not reported	Supervised
Sonsilphong, 2022 [141]	Engineering Conference Paper	Interdisciplinary	Gynecological Surgery	Laparoscopic Hysterectomy	Organs/Tissue, Surgical Instruments	Real	Private dataset(s)	n/a	Bounding box	unclear	Unclear	n/a	CNN	not reported	Supervised
Streckert, 2023 [142]	Engineering Conference Paper	Computer Science	Visceral / General Surgery	Robotic Colorectal Surgery	Surgical Instruments	Real, Synthetic, Also non-human data included	Public dataset(s)	EndoVis15	Semantic	unclear	Unclear	n/a	CNN	not reported	Supervised
Strong, 2024 [143]	Gastroenterology & Hepatology	Interdisciplinary	Visceral / General Surgery	Robotic Gastrectomy	Surgical Instruments	Real	Private dataset(s)	n/a	Semantic	unclear	Unclear	n/a	CNN	not reported	Supervised
Sun, 2022 [144]	Computer Science	Computer Science	Visceral / General Surgery	Unclear	Surgical Instruments	Real	Private dataset(s)	n/a	Classification	unclear	Unclear	n/a	CNN	not reported	Supervised
Takeuchi, 2023 [145]	Surgery	Interdisciplinary	Visceral / General Surgery	Laparoscopic Hernia Repair	Organs/Tissue, Technical guidance	Real	Private dataset(s)	n/a	Bounding box	CVAT	Yes (unclear criteria)	"set consisting of both positive examples (images where anatomical structured were present) and negative examples (images where structures were not present)."	CNN	not reported	Supervised
Tao, 2023 [146]	Engineering, Biomedical; Radiology, Nuclear Medicine & Medical Imaging; Engineering, Electrical & Electronic; Imaging Science & Photographic Technology; Computer Science, Interdisciplinary Applications	Computer Science	Visceral / General Surgery	Laparoscopic cholecystectomy	Surgical Instruments	Real	Public dataset(s)	Cholec80, m2cai-t	Classification	unclear	No	n/a	TM, CNN	"we propose to use a banded causal mask to limit the dependency range between frames while ensuring that future information is masked, thus supporting on-line surgical workflow analysis."	Supervised
Tokuyasu, 2021 [147]	Surgery	Interdisciplinary	Visceral / General Surgery	Laparoscopic cholecystectomy	Organs/Tissue	Real	Private dataset(s)	n/a	Semantic	unclear	Yes (clear criteria)	"As the degree of difficulty in LC increases in tandem with the extent of fibrosis and/or scarring inside the abdominal cavity, the technical platform of our system was established using videos with minimal fibrosis and/or scarring; videos with bleeding or less-visible landmarks were also excluded."	CNN	not reported	Supervised
Urrea, 2024 [148]	Pharmacology & Pharmacy; Biochemistry & Molecular Biology; Medicine, Research & Experimental	Computer Science	Visceral / General Surgery	Laparoscopic cholecystectomy	Organs/Tissue, Surgical Instruments,	Real	Public dataset(s)	CholecSeg8K	Semantic	PixelAnnotaion Tool	Unclear	n/a	CNN	Spatial–Temporal Convolutional Network (SP-TCN)	Supervised

					Technical guidance										
Valderrama, 2022 [149]	Preprint	Interdisciplinary	Urological Surgery	Robotic Prostatectomy	Surgical Instruments	Real	Private dataset(s)	PSI-AVA	Bounding box, Classification	unclear	Yes (unclear criteria)	"removing video segments with non-surgical content and potential patient identifiers"	TM, CNN	"We build upon the Multiscale Vision Transformer (MViT) model developed for video and image recognition tasks [9]. The connection between multiscale feature hierarchies with a transformer model, provides rich features to model the complex temporal cues of the surgical scene around each keyframe"	Supervised
Vardazaryan, 2018 [150]	Preprint	Computer Science	Visceral / General Surgery	Laparoscopic cholecystectomy	Surgical Instruments	Real	Public dataset(s)	Cholec80	Classification, Bounding box	unclear	Unclear	n/a	CNN	not reported	Weakly-supervised
Wagner, 2023 [151]	Radiology, Nuclear Medicine & Medical Imaging; Engineering, Biomedical; Computer Science, Interdisciplinary Applications; Computer Science, Artificial Intelligence	Interdisciplinary	Visceral / General Surgery	Laparoscopic cholecystectomy	Surgical Instruments	Real	Public dataset(s), Private dataset(s)	ROBUST-MIS	Classification	Anvil	Unclear	n/a	CNN	not reported	Supervised
Wang, 2023 [152]	Engineering Conference Paper	Computer Science	Visceral / General Surgery	Laparoscopic cholecystectomy	Surgical Instruments	Real	Public dataset(s)	Cholec80	Classification	unclear	Unclear	n/a	CNN	not reported	Supervised
Wang, 2017 [153]	Engineering Conference Paper	Computer Science	Visceral / General Surgery	Laparoscopic cholecystectomy	Surgical Instruments	Real	Public dataset(s)	m2cai-t	Classification	unclear	Unclear	n/a	CNN	not reported	Supervised
Wang, 2024 [154]	Preprint	Interdisciplinary	Visceral / General Surgery	Laparoscopic cholecystectomy	Surgical Instruments	Real	Public dataset(s)	Cholec80, m2cai-tl	Classification, Bounding box	unclear	Unclear	n/a	CNN	"HMM-stabilized deep learning model; LSTM-enhanced version of EndoNet and FCN compared to 4 frame-independent methods, ToolNet, EndoNet, SwinNet, and FR-CNN"	Semi-supervised
Wang, 2023 [155]	Surgery	Interdisciplinary	Urological Surgery	Robotic Partial Nephrectomy	Surgical Instruments	Real	Private dataset(s)	n/a	Semantic	unclear	Unclear	n/a	CNN	"mtCNN processes the sequence of instrument features across all the video frames"	Supervised
Wang, 2025 [156]	Radiology, Nuclear Medicine & Medical Imaging; Engineering, Biomedical; Computer Science, Interdisciplinary Applications; Computer Science, Artificial Intelligence	Computer Science	Urological Surgery	Robotic Prostatectomy	Surgical Instruments	Real	Public dataset(s)	GraSP	Bounding box, Semantic	unclear	Unclear	n/a	CNN, TM	Stereo-temporal set classifier (STSCls)	Supervised
Wang, 2021 [157]	Engineering Conference Paper	Computer Science	Visceral / General Surgery	Laparoscopic cholecystectomy	Surgical Instruments	Real	Public dataset(s)	m2cai-tl	Bounding box	unclear	Unclear	n/a	CNN	not reported	Supervised
Wang, 2020 [158]	Telecommunications; Computer Science, Information Systems; Engineering, Electrical & Electronic	Computer Science	Visceral / General Surgery	Laparoscopic cholecystectomy	Surgical Instruments	Real	Private dataset(s), Public dataset(s)	m2cai-tl	Bounding box	unclear	Unclear	n/a	CNN	not reported	Supervised
Wang, 2024 [159]	Preprint	Interdisciplinary	Visceral / General Surgery	Laparoscopic cholecystectomy, Robotic Liver Surgery	Surgical Instruments	Real	Public dataset(s)	Cholec80	Semantic, Classification	unclear	Unclear	n/a	TM	"We take temporal properties into account by inducing two temporal constraints for traditional paradigm, temporal equivariance and class-aware temporal semantic continuity constraints, together with temporal-enhanced pseudo masks generation; CAM"	Weakly-supervised
Wang, 2023 [160]	Robotics; Engineering, Biomedical	Computer Science	Visceral / General Surgery	Laparoscopic cholecystectomy	Surgical Instruments	Real	Public dataset(s)	m2cai-tl	Bounding box	unclear	Unclear	n/a	CNN	not reported	Supervised
Ward, 2022 [161]	Surgery	Interdisciplinary	Visceral / General Surgery	Laparoscopic cholecystectomy	Organs/Tissue, Others	Real	Private dataset(s)	n/a	Classification	unclear	Unclear	n/a	CNN	not reported	Supervised
Wei, 2024 [162]	Surgery; Engineering, Biomedical; Radiology, Nuclear Medicine & Medical Imaging	Computer Science	Visceral / General Surgery	Laparoscopic cholecystectomy, Laparoscopic Colectomy, Laparoscopic Rectal Resection, Laparoscopic Hernia Repair, Laparoscopic Sigmoid Resection	Surgical Instruments	Real	Public dataset(s)	ROBUST-MIS	Semantic	understand.ai	Unclear	n/a	TM, CNN	not reported	Supervised
Wei, 2023 [163]	Preprint	Interdisciplinary	Visceral / General Surgery	Laparoscopic Colectomy, Laparoscopic Rectal Resection, Laparoscopic Hernia Repair, Laparoscopic Sigmoid Resection	Surgical Instruments	Real, Also non-human data included	Public dataset(s)	ROBUST-MIS	Semantic	Understand.ai	Unclear	n/a	CNN	not reported	Semi-supervised

Wu, 2024 [164]	Computer Science, Interdisciplinary Applications; Biology; Mathematical & Computational Biology; Engineering, Biomedical	Interdisciplinary	Gynecological Surgery	Laparoscopic Hysterectomy	Surgical Instruments	Real	Public dataset(s)	AutoLaparo	Semantic	LabelMe	Unclear	n/a	CNN	not reported	Supervised
Xi, 2022 [165]	Engineering, Electrical & Electronic	Computer Science	Visceral / General Surgery	Laparoscopic cholecystectomy	Surgical Instruments	Real	Public dataset(s)	CholecT50	Classification	unclear	Unclear	n/a	Others/Unclear (Graph Convolutional Network (GCN))	not reported	Supervised
Xu, 2024 [166]	Preprint	Computer Science	Urological Surgery	Robotic Prostatectomy	Surgical Instruments	Real, Also non-human data included	Public dataset(s)	SAR-RARP	Semantic	unclear	Unclear	n/a	CNN, TM	not reported	Supervised, Others/unclear
Xue, 2022 [167]	Engineering, Biomedical	Computer Science	Visceral / General Surgery	Laparoscopic cholecystectomy	Surgical Instruments	Real, Also non-human data included	Public dataset(s)	m2cai-tl	Bounding box	unclear	Unclear	n/a	CNN	not reported	Supervised
Yamazaki, 2020 [168]	Surgery	Interdisciplinary	Visceral / General Surgery	Laparoscopic Gastrectomy	Surgical Instruments	Real	Private dataset(s)	n/a	Bounding box	unclear	Unclear	n/a	CNN	not reported	Supervised
Yamlahi, 2023 [169]	Preprint	Computer Science	Visceral / General Surgery	Laparoscopic cholecystectomy	Surgical Instruments	Real	Public dataset(s)	CholecT45	Classification	unclear	Unclear	n/a	TM	not reported	Supervised
Yang, 2024 [170]	Engineering Conference Paper	Computer Science	Visceral / General Surgery	Laparoscopic cholecystectomy	Organs/Tissue	Real	Private dataset(s)	n/a	Bounding box	Labelimg	Yes (unclear criteria)	"Out of the 101 surgical videos, 80 were selected based on their good visual effects and compliance with the selection criteria."	CNN	not reported	Supervised
Yang, 2023 [171]	Preprint	Interdisciplinary	Visceral / General Surgery	Laparoscopic cholecystectomy	Organs/Tissue, Surgical Instruments	Real	Public dataset(s)	CholecSeg8K	Semantic	PixelAnnotaion Tool	Unclear	n/a	CNN	not reported	Supervised
Yang, 2021 [172]	Engineering Conference Paper	Interdisciplinary	Visceral / General Surgery	Laparoscopic cholecystectomy	Surgical Instruments	Real	Public dataset(s)	Cholec80	Unclear	unclear	Yes (unclear criteria)	"13 videos from the dataset are eliminated due to bad segmentation performance."	CNN	not reported	Others/unclear
Yin, 2024 [173]	Preprint	Interdisciplinary	Visceral / General Surgery	Laparoscopic cholecystectomy	Organs/Tissue, Surgical Instruments, Technical guidance	Real	Public dataset(s)	CholecT45, Cholec80-CVS	Classification	unclear	Unclear	n/a	TM	"Prediction of following steps in the next frames"	Supervised
Yuan, 2024 [174]	Surgery; Engineering, Biomedical; Radiology, Nuclear Medicine & Medical Imaging	Computer Science	Visceral / General Surgery	Laparoscopic cholecystectomy	Surgical Instruments, Organs/Tissue	Real	Public dataset(s)	CholecT45, m2cai-tl, CholecSeg8k	Bounding box, Semantic, Classification	PixelAnnotaion Tool	Yes (unclear criteria)	"To reduce class-imbalance"	CNN, TM	not reported	Supervised
Zhang, 2024 [175]	Robotics	Interdisciplinary	Visceral / General Surgery	Laparoscopic cholecystectomy	Organs/Tissue	Real	Private dataset(s)	n/a	Semantic, Bounding box	unclear	Unclear	n/a	Others/Unclear (TAI-GNN)	"Temporal Graph: During the CTS process, video frame data is transformed into a sequence of spatial graphs. These graphs are then cascaded by connecting the same-type target nodes to each graph, forming a spatial-temporal graph"	Supervised
Zhang, 2023 [176]	Engineering Conference Paper	Computer Science	Visceral / General Surgery	Laparoscopic cholecystectomy	Surgical Instruments	Real	Public dataset(s)	Cholec80	Classification, Bounding box	LabelIMG	Unclear	n/a	CNN	DeepSORT (Simple Online and Realtime Tracker mit Deep Learning)	Supervised
Zhang, 2023 [177]	Engineering, Biomedical	Computer Science	Visceral / General Surgery	Laparoscopic cholecystectomy	Surgical Instruments	Real	Public dataset(s)	Cholec80	Classification	unclear	Unclear	n/a	TM, CNN	"MS-TCN, a recent state-of-the-art architecture in action segmentation, improved on previous approaches by adopting a fully convolutional architecture for processing the temporal dimension of the video"	Supervised
Zhang, 2020 [178]	Telecommunications; Computer Science, Information Systems; Engineering, Electrical & Electronic	Interdisciplinary	Visceral / General Surgery	Laparoscopic cholecystectomy	Surgical Instruments	Real	Public dataset(s), Private dataset(s)	m2cai-tl	Bounding box	unclear	Unclear	n/a	CNN	not reported	Supervised
Zhang, 2023 [179]	Computer Science, Interdisciplinary Applications; Computer Science, Information Systems; Mathematical & Computational Biology; Medical Informatics	Interdisciplinary	Visceral / General Surgery	Laparoscopic cholecystectomy	Surgical Instruments, Technical Guidance	Real	Private dataset(s)	n/a	Classification	LabelImg	Unclear	n/a	CNN	Spatial-temporal patterns	Supervised
Zhang, 2020 [180]	Surgery; Engineering, Biomedical; Radiology, Nuclear Medicine & Medical Imaging	Computer Science	Visceral / General Surgery	Laparoscopic cholecystectomy	Surgical Instruments	Real	Public dataset(s)	m2cai-t	Classification, Dot	unclear	Unclear	n/a	CNN	not reported	Supervised
Zhao, 2021 [181]	Radiology, Nuclear Medicine & Medical Imaging; Engineering, Biomedical; Computer Science, Interdisciplinary Applications; Computer Science, Artificial Intelligence	Interdisciplinary	Urological Surgery, Others	Robotic Prostatectomy	Surgical Instruments	Real, Also non-human data included	Private dataset(s)	n/a	Semantic	Viame	Unclear	n/a	CNN	Anchor matching mechanism	Supervised, Unsupervised
Zhao, 2023 [182]	Medical Informatics	Interdisciplinary	Visceral / General Surgery, Others	Laparoscopic cholecystectomy	Surgical Instruments	Real	Public dataset(s)	Cholec80	Bounding box	unclear	Unclear	n/a	CNN	"cross-layer aggregated attention detection network (CLAD-Net)"	Supervised

Zhao, 2021 [183]	Preprint	Interdisciplinary	Urological Surgery	Robotic Prostatectomy	Surgical Instruments	Real, Also non-human data included	Public dataset(s)	HKPWH	Semantic	unclear	Unclear	n/a	CNN	"In order to avoid the negative effect of the noisy supervision and increase the model robustness to tackle large temporal variation, we propose to selectively update the model by leveraging the available label of first frame to assess the quality of each pseudo mask."	Semi-supervised
Zhao, 2019 [184]	Surgery	Computer Science	Others, Visceral / General Surgery	Unclear	Surgical Instruments	Real, Also non-human data included	Unclear	unclear	Bounding box	unclear	Unclear	n/a	CNN, TM	"spatial transformer network (STN) for the location-determination process and a spatio-temporal context learning algorithm (STC) for the process of tracking frame by frame"	Supervised
Zheng, 2023 [185]	Surgery	Interdisciplinary	Urological Surgery, Visceral / General Surgery	Laparoscopic cholecystectomy, Laparoscopic Prostatectomy, Laparoscopic Partial Adrenal Resection, Laparoscopic Bladder Resection	Surgical Instruments	Real	Private dataset(s), Public dataset(s)	Cholec80	Semantic	LabelMe	Yes (clear criteria)	"Exclusion criteria included any of the following, (i) A visual pedestal, (ii) smoke, (iii) blurred image, or (iv) specular reflection."	CNN	not reported	Supervised
Zhou, 2024 [186]	Engineering Conference Paper	Interdisciplinary	Visceral / General Surgery	Laparoscopic cholecystectomy	Organs/Tissue, Technical guidance	Real	Public dataset(s)	Cholec80	Semantic	unclear	Unclear	n/a	TM	not reported	Supervised
Zhou, 2022 [187]	Engineering Conference Paper	Computer Science	Visceral / General Surgery	Laparoscopic cholecystectomy	Surgical Instruments	Real	Public dataset(s)	m2cai-tl	Bounding box	unclear	Unclear	n/a	CNN	not reported	Supervised
Zygomalas, 2024 [188]	Surgery	Interdisciplinary	Visceral / General Surgery	Laparoscopic Hernia Repair	Organs/Tissue, Surgical Instruments	Real	Private dataset(s), unclear	unclear	Bounding box	SuperAnnotate	Unclear	n/a	CNN	not reported	Supervised

Abbreviations: *n/a: not applicable, CNN: Convolutional Neural Network, TM: Transformer Model*

Footnotes:

a. Searched on: https://clarivate.com/academia-government/scientific-and-academic-research/research-funding-analytics/journal-citation-reports/. If the journal was not available, for example, because it is too new, the criteria from the official journal website were used. If it is a preprint journal, it was marked as "Preprint." If it is a conference paper, it was labeled as "Engineering Conference Paper," since they are exclusively in the field of engineering.
b. If the name was provided and if it is a dataset consisting of human data.

Supplementary Table 4: Studies and study characteristics included in the systematic review and meta-analysis (2)

The following table presents an overview of all included studies, detailing key characteristics across four main categories: **Validation Strategies, Dataset Size, Model Performance Assessment, Uncertainty Estimation**.

Publication	Validation Strategies					Dataset Size							Model Performance Assessment						Uncertainty Estimation			
First author, year [reference]	Validation method	If cross-validation X Fold	Validation distribution[a]	Dataset Validation[b]	Independence of test samples when splitting the data	Number of Patients[c]	Number of institutions[c]	Dataset Size[d]	No. videos training + validation	No. frames training + validation	No. videos testing	No. frames testing	Object detection metrics	Calculation of performance metrics[e]	Temporal metrics use	Visual Reporting	Additional Imaging Modality	Real-time modeling (inference time)	Metrics for reporting model uncertainty and variability	Mode of reporting model uncertainty and prediction variability	Calculation of model uncertainty and prediction variability[e]	Other statistical tests[f]
Acharya, 2022 [1]	Cross-Validation, Hold-out	10	unclear	Internal Retrospective	"Yes, on frame level"	102	1	25682 frames	unclear	unclear	unclear	unclear	Accuracy, Recall, Precision	Unclear	no	no	No	unclear	SD	Exact numbers	Unclear	none
Al Hajj, 2018 [2]	Hold-out	n/a	45/5/50	Internal Retrospective	"Yes, on patient and frame level"	80	1	80 videos	40	unclear	40	unclear	ROC Curve, mAP	Unclear	no	others	No	2.87 ms	None	None	none	"Frequentist parametric (e.g., t-test)"
Alapatt, 2021 [3]	Hold-out	n/a	unclear	Internal Retrospective	"Yes, on frame level"	201	1	1933 frames	unclear	unclear	unclear	unclear	F1-Score, mIoU, Accuracy	Unclear	no	Qualitative results	No	n/a	None	None	none	none
Ali, 2022 [4]	Hold-out	n/a	unclear	Internal Retrospective	"Yes, on frame level"	15	1	2812 frames	unclear	2531	unclear	281	mAP	Unclear	no	Qualitative results	No	n/a	None	None	none	"Frequentist parametric (e.g., t-test)"
Alkhamaiseh, 2023 [5]	Cross-Validation	5	unclear	Internal Retrospective	"Yes, on frame level"	200	unclear	1550 frames	unclear	1395	unclear	155	DSC, Precision, Recall, IoU, Others	Over frame-level averages of n cross-validation folds	no	Qualitative results	No	24 ms	None	None	none	none
Alshirbaji, 2021 [6]	Cross-Validation	6	50/50	Internal Retrospective	"Yes, on patient and frame level"	80	1	80 videos	40	unclear	40	unclear	mAP	Over sample-level averages of n cross-validation folds	no	Class activation map	No	n/a	SD	Graph	Over sample-level averages of n cross-validation folds	none
Alshirbaji, 2021 [7]	Hold-out	n/a	unclear	Internal Retrospective	Unclear	100	3	92305 frames	80, 20	unclear	20/80	unclear	AP	Unclear	no	no	No	n/a	SD	Graph	Unclear	none
Alshirbaji, 2018 [8]	Hold-out	n/a	50/50	Internal Retrospective	"Yes, on patient and frame level"	80	1	80 videos	40	unclear	40	unclear	Others, Accuracy, Recall, Precision	Unclear	no	others	No	n/a	None	None	none	none
Ángeles-Cerón, 2021 [9]	Hold-out	n/a	unclear	Internal Retrospective	"Yes, on frame level", "Yes, on patient and frame level"	unclear	1	4987 frames	unclear	4239	unclear	748	Others	Unclear	no	Qualitative results	No	>45 FPS	IQR	Graph	Unclear	none
Ángeles-Cerón, 2022 [10]	Hold-out	n/a	unclear	Internal Retrospective	"Yes, on patient and frame level", "Yes, on frame level"	30	1	9044 frames	unclear	4987	unclear	4057	Others	Over n frames in test set	no	Qualitative results, others, Feature Map	No	24 FPS	IQR	Graph	Over n frames in test set	none
Aoyama, 2024 [11]	Hold-out	n/a	45/5	Prospective Interventional	"Yes, on patient and frame level"	60	1	2771 frames	unclear	2493	unclear	278	DSC	Unclear	no	Qualitative results	No	210 ms	None	None	none	"Frequentist non-parametric (e.g., Mann-Whitney)"
Arabian, 2022 [12]	Hold-out	n/a	50/x/50	Internal Retrospective	"Yes, on patient and frame level"	80	1	80 Videos	40	unclear	40	unclear	mAP	Over n frames in test set	no	Class activation map	No	n/a	SD	Graph	Unclear	none
Arabian, 2023 [13]	Hold-out	n/a	50/50	Internal Retrospective, External Retrospective	"Yes, on patient and frame level"	80	1	80 videos	40	unclear	40	unclear	mAP	Over n frames in test set	no	no	No	n/a	SD	Exact numbers, Graph	Unclear	none
Aspart, 2022 [14]	Cross-Validation	5	90/10	Internal Retrospective	"Yes, on patient and frame level"	300	1	122470 frames	271	111154	29	11316	ROC Curve, Sensitivity, Specificity	Unclear	no	Class activation map	No	16 FPS	SD	Exact numbers	Unclear	none
Attia, 2017 [15]	Hold-out	n/a	unclear	Internal Retrospective	"Yes, on frame level"	4	1	unclear	unclear	unclear	unclear	unclear	IoU, Sensitivity, Specificity, Accuracy	Unclear	no	Qualitative results	No	n/a	None	None	none	none
Ayobi, 2024 [16]	Cross-Validation	2	unclear	Prospective Observational	"Yes, on patient and frame level"	13	1	13 videos	8	unclear	5	unclear	mAP, mIoU, Others	Unclear	unclear	Qualitative results	No	n/a	SD	Exact numbers	Unclear	none
Bai, 2023 [17]	Hold-out	n/a	unclear	Internal Retrospective	"Yes, on frame level"	15	1	2811 frames	unclear	unclear	unclear	unclear	Accuracy, mIoU	Unclear	no	Qualitative results	No	n/a	None	None	none	none
Bakker, 2024 [18]	Hold-out	n/a	90/10	Internal Retrospective	"Yes, on patient and frame level"	111	2	1694 frames	unclear	1522	unclear	172	DSC, Others	Unclear	no	Qualitative results	No	n/a	SD	Exact numbers, Graph	Unclear	Other
Bamba, 2021 [19]	Hold-out	n/a	unclear	Internal Retrospective	"Yes, on patient and frame level", "Yes, on frame level"	10	1	1270 frames	unclear	1070	unclear	200	Accuracy, Precision, Recall, mAP, IoU	Unclear	no	Qualitative results	No	unclear	CI	Exact numbers	Unclear	none

Bamba, 2021 [20]	Hold-out	n/a	unclear	Prospective Observational	"Yes, on patient and frame level"	17	1	1673 frames	11	1173	6	500	Accuracy, mAP, Precision, Recall, IoU, Covidence Score	Unclear	no	Qualitative results	No	n/a	CI	Exact numbers	Unclear	none
Ban, 2023 [21]	Cross-Validation, Hold-out	3, 5	90/10	Internal Retrospective	"Yes, on patient and frame level"	345	unclear	345 videos	unclear	unclear	unclear	unclear	AP, Accuracy, Others	Unclear	no	others	No	n/a	SD	Exact numbers	Unclear	none
Batić, 2024 [22]	unclear	unclear	unclear	External Retrospective	"Yes, on frame level"	unclear	unclear	743724 frames	unclear	unclear	unclear	unclear	mIoU, mAP	Unclear	no	Qualitative results	No	n/a	SD	Exact numbers	Unclear	none
Batić, 2023 [23]	Hold-out	n/a	unclear	External Retrospective	"Yes, on frame level"	unclear	unclear	>700000 frames	unclear	unclear	unclear	unclear	mAP	Unclear	no	Qualitative results	No	unclear	SD	Exact numbers	Unclear	none
Boonkong, 2022 [24]	unclear	n/a	unclear	Internal Retrospective	"Yes, on frame level"	unclear	unclear	870 frames	unclear	unclear	unclear	unclear	Precision, Recall, F1-Score	Unclear	no	Qualitative results	No	n/a	None	None	none	none
Brandenburg, 2023 [25]	Hold-out	n/a	ca 85/15	Prospective Observational, Prospective Interventional	"Yes, on patient and frame level"	26	2	14004 frames	22	13400	4	604	F1-Score, Precision, Recall	Over n frames in test set	no	others	No	12 FPS	SD	Exact numbers, Graph	Over n frames in test set	"Frequentist parametric (e.g., t-test)"
Casella, 2021 [26]	Hold-out	n/a	75/12.5/12.5	Internal Retrospective	"Yes, on patient and frame level"	8	1	1871 frames	7	1631	1	240	Precision, Recall, DSC	Over n frames in test set	no	Qualitative results	No	n/a	Standard Error	Exact numbers	Over n frames in test set	"Frequentist non-parametric (e.g., Mann-Whitney)"
Chen, 2024 [27]	Hold-out	n/a	unclear	Internal Retrospective, External Retrospective	"Yes, on frame level"	unclear	2	unclear (0ver 3 mio. frames)	unclear	unclear	unclear	unclear	mIoU, mDSC	Unclear	no	Qualitative results	No	n/a	None	None	none	"Frequentist non-parametric (e.g., Mann-Whitney)"
Chen, 2013 [28]	Hold-out	n/a	n/a	Prospective Observational	"Yes, on patient and frame level"	1	1	907 frames	1	10	1	897	Others	Over n frames in test set	no	Qualitative results	No	8 FPS	None	None	none	none
Chen, 2017 [29]	Hold-out	n/a	unclear	Internal Retrospective	"Yes, on patient and frame level"	unclear	unclear	1000 frames	unclear	unclear	unclear	unclear	Accuracy	Over n frames in test set	no	Qualitative results	No	25.41 FPS	None	None	none	none
Choi, 2017 [30]	Hold-out	n/a	70/30	Internal Retrospective	"Yes, on patient and frame level"	10	1	10 videos	7	unclear	3	unclear	Recall, Precision, mAP	"Over n independent samples (i.e., one patient/video) in independent test set"	no	Qualitative results	No	48.9 FPS	Other	Exact numbers	Other	none
Ciaparrone, 2020 [31]	Hold-out	n/a	unclear	Internal Retrospective	"Yes, on patient and frame level"	14	unclear	4955 frames	unclear	3485	unclear	1470	AP	Over n frames in test set	no	Qualitative results	No	400 ms	None	None	none	none
Colleoni, 2024 [32]	Hold-out	n/a	unclear	Internal Retrospective	"Yes, on frame level"	unclear	unclear	254678 frames	1772	230455	183	24223	mIoU	Over n frames in test set	no	Qualitative results	No	n/a	SD	Exact numbers	Over n frames in test set	none
Colleoni, 2022 [33]	Hold-out	n/a	unclear	Internal Retrospective	Unclear	23	1	7236 frames	unclear	6248	unclear	988	IoU	Unclear	no	Qualitative results	No	n/a	None	None	none	none
Daneshgar Rahbar, 2023 [34]	Hold-out	n/a	unclear	External Retrospective	"Yes, on frame level"	unclear	unclear	unclear	unclear	unclear	unclear	unclear	mDSC, mIoU, Accuracy	Over n frames in test set	no	Qualitative results	No	34.2 ms	None	None	none	none
Davila, 2023 [35]	unclear	unclear	unclear	Internal Retrospective	Unclear	80	1	80 videos	unclear	86331	unclear	unclear	AP, Accuracy	Unclear	no	no	No	n/a	Standard Error	Graph	Unclear	none
De Backer, 2023 [36]	Hold-out	n/a	70/20/10	Internal Retrospective	"Yes, on patient and frame level"	67	1	15100 frames	52	13592	5	1508	IoU, DSC	Over n frames in test set	no	Qualitative results	No	500 ms	None	None	none	none
den Boer, 2023 [37]	Cross-Validation	5	80/20	Internal Retrospective	"Yes, on patient and frame level"	83	1	1050 frames	66	850	17	200	DSC, Others	Unclear	no	Qualitative results	No	39 FPS	IQR	Exact numbers	Unclear	"Frequentist non-parametric (e.g., Mann-Whitney)"
Derathé, 2020 [38]	Cross-Validation	10	unclear	Internal Retrospective	"Yes, on patient and frame level"	29	1	29 videos	unclear	unclear	unclear	unclear	Accuracy, Sensitivity, Specificity	Unclear	no	no	No	n/a	SD	Exact numbers, Graph	Unclear	none
Du, 2019 [39]	unclear	n/a	unclear	External Retrospective, Internal Retrospective	Unclear	unclear	unclear	unclear	unclear	unclear	unclear	unclear	Others	Unclear	no	Qualitative results	No	unclear	None	None	none	none
Endo, 2023 [40]	unclear	n/a	80/20	Internal Retrospective	"Yes, on patient and frame level"	115	1	1754 frames	unclear	unclear	unclear	unclear	Others	Unclear	no	Qualitative results	No	unclear	None	None	none	none
Fernandez-Rodríguez, 2024 [41]	Hold-out	n/a	58/22/20	Internal Retrospective	"Yes, on patient and frame level"	80	1	8080 frames	13	6480	4	1600	DSC, Recall, Precision	Over sample-level averages of n cross-validation folds	no	Qualitative results	No	n/a	SD	Exact numbers	Over sample-level averages of n cross-validation folds	none
Fuentes-Hurtado, 2019 [42]	Cross-Validation, Hold-out	5	unclear	Internal Retrospective	"Yes, on patient and frame level"	29	2	50366 frames	unclear	unclear	unclear	unclear	IoU, Others	Over sample-level averages of n cross-validation folds	no	Qualitative results, others	No	n/a	None	None	none	none
Fujinaga, 2023 [43]	unclear	unclear	unclear	Prospective Interventional	"Yes, on patient and frame level"	122	1	1826 frames	unclear	unclear	unclear	unclear	DSC	Unclear	no	Qualitative results	No	30 FPS	SD	Exact numbers	Unclear	Other
Ghamsarian, 2024 [44]	Cross-Validation	4	75/x/25	Internal Retrospective	Unclear	108	1	158 frames	unclear	119	unclear	39	IoU, DSC	Unclear	no	no	No	n/a	None	None	none	none
Gitau, 2024 [45]	Hold-out	n/a	unclear	Internal Retrospective	"Yes, on frame level"	unclear	1	3299 frames	unclear	unclear	unclear	unclear	Accuracy	Over n frames in test set	no	no	No	n/a	None	None	none	none
Grammatikopoulou, 2023 [46]	Hold-out	n/a	85/5/10 (PN) 75/25 (CholecSeg8k)	Internal Retrospective	"Yes, on patient and frame level"	154	2	61080 frames	126	unclear	18	unclear	mIoU	Over n frames in test set	TC: 56.13 (PN), 87.26 (Cholec)	Qualitative results	No	n/a	None	None	none	none

Guédon, 2021 [47]	Cross-Validation	5	24/9/x (LC) 26/9/x (TLH)	Internal Retrospective	"Yes, on patient and frame level"	68	1	68 videos	50	unclear	18	unclear	Accuracy, Precision, Recall	Unclear	no	no	No	n/a	None	None	none	none
Hasan, 2021 [48]	Hold-out	n/a	unclear	Internal Retrospective	Unclear	4	1	unclear	unclear	unclear	unclear	unclear	DSC	Over n frames in test set	no	Qualitative results	No	n/a	SD	Exact numbers	Over n frames in test set	none
Huang, 2022 [49]	Hold-out	n/a	unclear	External Retrospective	"Yes, on frame level"	unclear	unclear	48702 frames	unclear	34320	unclear	14382	IoU, DSC, Others	Unclear	no	Qualitative results, Depth map	No	5.8 ms / 172 FPS	SD	Exact numbers	Unclear	none
Jalal, 2022 [50]	Hold-out	n/a	50/50	Internal Retrospective	"Yes, on patient and frame level"	80	1	15691 frames (bounding boxes)	40	unclear	40	unclear	mDSC, Precision, Others	Over n frames in test set	no	no	No	n/a	None	None	none	none
Jalal, 2023 [51]	Hold-out	n/a	50/50	Internal Retrospective	"Yes, on patient and frame level"	80	1	unclear	40	unclear	40	unclear	mAP, F1-Score	Over n frames in test set	no	Qualitative results, Localisation Map	No	20 ms	None	None	none	none
Jamal, 2024 [52]	Hold-out	n/a	unclear	Internal Retrospective	"Yes, on patient and frame level", "Yes, on frame level"	88	unclear	26130 frames	unclear	unclear	unclear	unclear	IoU	Unclear	no	no	Other	n/a	None	None	none	none
Jang, 2023 [53]	unclear	n/a	unclear	Internal Retrospective	Unclear	unclear	unclear	157 frames	unclear	137	unclear	20	Others	Unclear	no	Qualitative results	No	n/a	None	None	none	none
Jaspers, 2024 [54]	Cross-Validation	5	77/23	External Retrospective	"Yes, on patient and frame level"	162	3	9231 frames	unclear	7801	unclear	1430	DSC	Unclear	no	Qualitative results	No	n/a	SD	Graph	Unclear	none
Jearanai, 2023 [55]	Hold-out	n/a	unclear	Internal Retrospective, Prospective Interventional	"Yes, on frame level"	unclear	1	3600 frames	unclear	3200	unclear	400	Precision, Recall, mAP	Over n frames in test set	no	Qualitative results	No	unclear	None	None	none	none
Jha, 2021 [56]	Hold-out	n/a	unclear	Internal Retrospective	"Yes, on frame level"	30	1	5983 frames	unclear	5385	unclear	598	mIoU, DSC, Precision, Recall, Accuracy, Others	Unclear	no	Qualitative results	No	101.36 FPS	None	None	none	none
Jin, 2020 [57]	Hold-out	n/a	50/50	Internal Retrospective	"Yes, on patient and frame level"	80	1	80 videos	40	unclear	40	unclear	mAP	"Over n independent samples (i.e., one patient/video) in independent test set"	no	Qualitative results	No	n/a	SD	Exact numbers, Graph	"Over n independent samples (i.e., one patient/video) in independent test set"	none
Jin, 2018 [58]	Hold-out	n/a	67/33 (class)	Internal Retrospective	Unclear	15	1	2532 frames (bounding box), 23000 (classification)	10 (classification)	2026 (bounding box)	5 (classification)	506 (bounding box)	mAP	Unclear	no	Qualitative results, Heat map, others	No	5 FPS	None	None	none	none
Kamrul Hasan, 2021 [59]	Hold-out	n/a	unclear	Internal Retrospective	"Yes, on frame level"	30	1	Semantic: 635 frames Classification: 4270 frames	unclear	Semantic: 508, Classification: 1016	unclear	Semantic: 127, Classification: 3254	AP, Accuracy, DSC, IoU, Sensitivity, Specificity, Others	Unclear	no	Qualitative results	No	unclear	Other	Exact numbers, Graph	Unclear	none
Kanakatte, 2020 [60]	Hold-out	n/a	50/50	Internal Retrospective	"Yes, on frame level"	10	1	1210 frames	unclear	960	unclear	250	mAP	Unclear	no	Qualitative results	No	n/a	None	None	none	none
Kawamura, 2023 [61]	Hold-out	n/a	unclear	Internal Retrospective	"Yes, on patient and frame level"	72	1	23793 frames	52	unclear	20	unclear	Precision, Recall, F1-Score, Specificity, Accuracy	Unclear	no	Class activation map	No	6 FPS	None	None	none	none
Khalid, 2023 [62]	Others	n/a	unclear	External Retrospective	"Yes, on patient and frame level"	22	unclear	22 videos	n/a	n/a	n/a	n/a	Others	Unclear	no	Qualitative results	No	unclear	CI	Exact numbers	Unclear	none
Khalid, 2023 [63]	unclear	n/a	unclear	Internal Retrospective	Unclear	unclear	2	unclear	unclear	unclear	unclear	unclear	Precision, Recall, F1-Score, Others	Unclear	no	Qualitative results, Depth map	No	n/a	None	None	none	none
Kim, 2024 [64]	Hold-out	n/a	50/50	Internal Retrospective	"Yes, on frame level"	unclear	1	1300 frames	unclear	1000	unclear	300	IoU, Precision, Recall, mAP, Others	Unclear	no	Qualitative results	No	unclear	None	None	none	none
Kinoshita, 2024 [65]	Hold-out	n/a	unclear	Internal Retrospective	"Yes, on patient and frame level"	75	2	800 frames	70	740	5	60	IoU, DSC	Unclear	no	Qualitative results	No	60 FPS	None	None	none	Other
Kitaguchi, 2023 [66]	Hold-out	n/a	unclear	Prospective Interventional	"Yes, on patient and frame level"	Ureter: 344, Nerves: 249	1	25122 frames (Ureter), 18176 frames (Nerve)	288 (Ureter), 194 (Nerve)	20506 (Ureter), 14577 (Nerve)	56 (Ureter) 55 (Nerve)	4616 (Ureter), 3599 (Nerve)	Precision, Recall, DSC	Over n frames in test set	no	Qualitative results	No	8-10 FPS	None	None	none	Other
Kitaguchi, 2022 [67]	Cross-Validation, Hold-out	5	unclear	Internal Retrospective	"Yes, on patient and frame level"	337	unclear	38628 frames	unclear	unclear	unclear	unclear	mAP	Unclear	no	Qualitative results	No	unclear	SD	Exact numbers	Unclear	none

Kitaguchi, 2022 [68]	Hold-out	n/a	4 different	Internal Retrospective	"Yes, on patient and frame level"	128	5	5238 frames	94	4419	34	819	IoU, AP, mAP	Over n frames in test set	no	Qualitative results	No	n/a	SD	Exact numbers, Graph	Over n frames in test set	none
Kletz, 2019 [69]	Hold-out	n/a	unclear	Internal Retrospective	"Yes, on frame level"	unclear	unclear	333 frames	unclear	267	unclear	66	AP, Recall	Unclear	no	Qualitative results	No	n/a	None	None	none	none
Kolbinger, 2023 [70]	Hold-out, Cross-Validation	4	unclear	Internal Retrospective	"Yes, on patient and frame level"	32	1	13195 frames	24	uncelar	8	unclear	F1-Score, IoU, Precision, Recall, Specificity	Over n frames in test set	no	Qualitative results	No	28 ms	SD	Graph, Exact numbers	Over n frames in test set	none
Kolbinger, 2024 [71]	Cross-Validation	4	unclear	Internal Retrospective	"Yes, on patient and frame level"	33	1	9037 frames	unclear	unclear	unclear	unclear	F1-Score, IoU, Precision, Recall, Specificity	Over n frames in test set	no	Qualitative results	No	n/a	SD	Exact numbers, Graph	Over n frames in test set	none
Kolbinger, 2024 [72]	Hold-out	n/a	unclear	Internal Retrospective	"Yes, on patient and frame level"	32	1	13195 frames	24	uncelar	8	unclear	Accuracy, Precision, Recall, F1-Score, IoU, Specificity, Others	Over n frames in test set	no	no	No	n/a	None	None	none	none
Kondo, 2021 [73]	Cross-Validation	4	unclear	Internal Retrospective	"Yes, on patient and frame level"	80	1	80 videos	60	unclear	20	unclear	Precision, Recall, F1-Score	Over n frames in test set	no	no	No	n/a	None	None	none	none
Konduri, 2024 [74]	Hold-out	n/a	unclear	Internal Retrospective	"Yes, on patient and frame level", "Yes, on frame level"	unclear	unclear	unclear	unclear	unclear	unclear	unclear	Accuracy, Precision, Recall, F1-Score	Unclear	no	no	No	n/a	None	None	none	none
Kong, 2021 [75]	Cross-Validation	4	unclear	Internal Retrospective	"Yes, on patient and frame level"	2	1	2760 frames	unclear	unclear	unclear	unclear	IoU, DSC, Precision, Recall	"Over n independent samples (i.e., one patient/video) in independent test set"	no	Qualitative results	No	n/a	SD	Exact numbers	"Over n independent samples (i.e., one patient/video) in independent test set"	none
Kumazu, 2021 [76]	Hold-out	n/a	60/10/30	Internal Retrospective	"Yes, on patient and frame level"	33	1	1880 frames	23	1800	10	80	Recall, F1-Score, DSC	Over n frames in test set	no	Qualitative results	No	30 FPS	SD	Exact numbers	Over n frames in test set	Other
Kumazu, 2025 [77]	Hold-out	n/a	60/10/30	Prospective Observational	"Yes, on patient and frame level"	93	unclear	31880 frames	unclear	unclear	unclear	unclear	DSC	Over n frames in test set	no	Qualitative results	No	unclear	SD	Exact numbers	Over n frames in test set	none
Labrunie, 2022 [78]	Cross-Validation	10	unclear	Internal Retrospective	"Yes, on patient and frame level"	68	unclear	1415 frames	unclear	unclear	unclear	unclear	Others	Unclear	no	others	No	n/a	Other	Exact numbers	Unclear	none
Lam, 2022 [79]	Hold-out	n/a	unclear	Prospective Observational	"Yes, on frame level"	5	1	2600 frames	3	2600	2	unclear	mAP, F1-Score	Over n frames in test set	no	Qualitative results, others	No	n/a	SD	Exact numbers	Over n frames in test set	none
Laplante, 2022 [80]	Cross-Validation	10	unclear	External Retrospective	"Yes, on patient and frame level"	333	143	333 videos	308	unclear	25	47	IoU, F1-Score, DSC	Unclear	no	Qualitative results, Probability map	No	unclear	SD	Exact numbers	Unclear	none
Lavanchy, 2021 [81]	Cross-Validation	10	unclear	Internal Retrospective	"Yes, on frame level"	242	1	13823 frames	unclear	10935	unclear	2888	AP, Recall	Unclear	no	Qualitative results	No	n/a	SD	Exact numbers	Unclear	"Frequentist parametric (e.g., t-test)"
Le, 2023 [82]	Hold-out	n/a	unclear	Internal Retrospective	"Yes, on patient and frame level"	15	1	2811 frames	unclear	1968	unclear	843	Precision, Recall, mAP, IoU	Over n frames in test set	no	Qualitative results	No	n/a	None	None	none	none
Lee, 2024 [83]	Hold-out	n/a	unclear	Internal Retrospective	Unclear	47	2	18120 frames	n/a	n/a	n/a	n/a	mIoU, AP	Unclear	no	Qualitative results	No	n/a	None	None	none	none
Leifman, 2024 [84]	Hold-out	n/a	80/20	Prospective Observational, Internal Retrospective	"Yes, on patient and frame level"	740	6	740 videos	560	unclear	180	unclear	Confusion Matrix, Others, Sensitivity, Specificity	Unclear	no	Qualitative results	No	49.2 ms	CI	Exact numbers	Unclear	none
Leifmann, 2022 [85]	Hold-out	n/a	unclear	Internal Retrospective	"Yes, on patient and frame level", "Yes, on frame level"	30	1	12764 frames (incl. non-human)	unclear	unclear	unclear	unclear	IoU, DSC	Unclear	no	Qualitative results	No	n/a	None	None	none	none
Li, 2025 [86]	Hold-out	n/a	unclear	Internal Retrospective	Unclear	2200	unclear	901000 frames	unclear	unclear	unclear	unclear	Precision, Recall, mAP	Unclear	no	no	No	100.8 ms	None	None	none	none
Li, 2023 [87]	Hold-out	n/a	unclear	External Retrospective	"Yes, on frame level", "Yes, on patient and frame level"	117	1	14080 frames	17	8080	100	6000	IoU, DSC	Unclear	no	Qualitative results	No	n/a	None	None	none	none
Liao, 2024 [88]	Hold-out	n/a	unclear	Internal Retrospective	"Yes, on patient and frame level", "Yes, on frame level"	80	1	80 videos	63 (incl. 5296 TOP clips)	unclear	17	3000	Accuracy, IoU, Others	Unclear	no	Qualitative results, Class activation map	No	n/a	None	None	none	none
Lin, 2024 [89]	Hold-out		unclear	Internal Retrospective	"Yes, on frame level"	unclear	1	14480 frames	145 snips	unclear	36 snips	unclear	mAP	Unclear	no	Qualitative results	No	51 ms	None	None	none	"Frequentist non-parametric (e.g., Mann-Whitney)"
Lin, 2021 [90]	Cross-Validation	3	75/12.5/12.5	Internal Retrospective	"Yes, on patient and frame level"	8	1	10040 frames	7	unclear	1	unclear	DSC, IoU	Unclear	no	Qualitative results	No	6.4 ms	SD	Exact numbers	Unclear	none

Liu, 2022 [91]	Hold-out	n/a	32/4/5	External Retrospective	"Yes, on patient and frame level"	41	1	5533 frames	unclear	4980	unclear	553	Precision, Recall, IoU, AP	Unclear	no	no	No	n/a	None	None	none	Other
Liu, 2024 [92]	Hold-out	n/a	unclear	Internal Retrospective	"Yes, on frame level"	unclear	unclear	3000 frames	unclear	2250	unclear	750	mIoU, Others	Unclear	no	Qualitative results	No	29.14 FPS	None	None	none	none
Liu, 2022 [93]	Hold-out	n/a	50/30/20	Internal Retrospective	"Yes, on patient and frame level"	80	1	80 videos	40	unclear	40	unclear	mAP	Other	no	Qualitative results, Heat map	No	50 FPS, 20 ms	SD, Standard Error	Graph	Other	none
Lou, 2023 [94]	Hold-out, unclear	n/a	unclear	Internal Retrospective	"Yes, on frame level"	29	1	816 frames	unclear	662	unclear	154	DSC, IoU, F1-Score	Unclear	no	Qualitative results	No	40 FPS	Other	Exact numbers	Unclear	none
Loukas, 2020 [95]	Cross-Validation	10	unclear	Internal Retrospective	"Yes, on frame level"	unclear	1	482 frames	unclear	unclear	unclear	unclear	Accuracy	Unclear	no	Qualitative results	No	n/a	SD, Other	Exact numbers	Unclear	"Frequentist parametric (e.g., t-test)"
Loza, 2024 [96]	Hold-out	n/a	50/30/20	Internal Retrospective	"Yes, on frame level"	15	1	2532 frames	unclear	2248	unclear	563	mAP	Over n frames in test set	no	Qualitative results	No	36 FPS	None	None	none	none
Maack, 2024 [97]	Hold-out	n/a	unclear	Internal Retrospective	"Yes, on patient and frame level"	32	1	13195 frames	24	uncelar	8	unclear	mDSC	Unclear	no	Qualitative results	No	n/a	None	None	none	none
Madad Zadeh, 2020 [98]	Hold-out	n/a	unclear	Internal Retrospective	"Yes, on patient and frame level"	8	1	461 frames	unclear	361	unclear	100	IoU, Accuracy	Unclear	no	Qualitative results	No	n/a	None	None	none	none
Madani, 2022 [99]	Cross-Validation	10	unclear	External Retrospective	"Yes, on frame level"	290	136	2627 frames	unclear	unclear	unclear	unclear	F1-Score, IoU, Accuracy, Sensitivity, Specificity, NPV, PPV	Over frame-level averages of n cross-validation folds	no	Qualitative results, Heat map, Probability map	No	<10 ms	SD	Exact numbers	Over frame-level averages of n cross-validation folds	Other
Maqbool, 2020 [100]	Hold-out	n/a	unclear	Internal Retrospective	"Yes, on patient and frame level"	2	1	307 frames	unclear	245	unclear	62	Precision, Recall, F1-Score, IoU	Unclear	no	Qualitative results	No	n/a	None	None	none	none
Marullo, 2023 [101]	Hold-out	n/a	unclear	Internal Retrospective	"Yes, on patient and frame level", "Yes, on frame level"	26	1	318 frames	unclear	232	unclear	86	Accuracy, DSC	Over n frames in test set	no	Qualitative results	No	15 FPS	None	None	none	none
Mascagni, 2022 [102]	Cross-Validation	5	60/20/20	Internal Retrospective	Unclear	200	1	2854 frames	unclear	unclear	unclear	unclear	IoU, AP	Unclear	no	Qualitative results, Saliency Map	No	n/a	SD	Exact numbers	Unclear	none
Matsumoto, 2024 [103]	unclear	n/a	66/34	Internal Retrospective	"Yes, on frame level"	18	1	18 Videos	6	1080	12	unclear	DSC, Accuracy	Unclear	no	Qualitative results	No	unclear	None	None	none	"Frequentist non-parametric (e.g., Mann-Whitney)"
Mehta, 2024 [104]	Hold-out	n/a	80/10/10	Internal Retrospective	"Yes, on patient and frame level"	1107	25	65000 frames	997	unclear	110	unclear	F1-Score, Precision, Recall, DSC	Over sample-level averages of n cross-validation folds	no	Qualitative results, others	No	12.5 FPS	SD	Exact numbers	Over sample-level averages of n cross-validation folds	none
Mishra, 2017 [105]	Hold-out	n/a	67/33	Internal Retrospective	"Yes, on patient and frame level"	15	1	15 Videos	10	unclear	5	unclear	Accuracy	Unclear	no	Qualitative results	No	2.42 ms	None	None	none	none
Murali, 2024 [106]	Hold-out	n/a	unclear	Internal Retrospective	Unclear	50	1	493 frames	40	unclear	10	unclear	mIoU, mDSC	Unclear	no	no	No	n/a	None	None	none	none
Murali, 2023 [107]	Hold-out	n/a	unclear	Internal Retrospective	"Yes, on patient and frame level"	201	1	11090 frames	unclear	unclear	unclear	unclear	mAP	Unclear	no	no	No	n/a	SD	Exact numbers	Unclear	none
Murali, 2022 [108]	Hold-out	n/a	60/20/20	Internal Retrospective	"Yes, on patient and frame level"	201	1	11090 frames	161	unclear	40	unclear	mAP, DSC	Unclear	no	Qualitative results	No		SD	Graph	Unclear	none
Myo, 2024 [109]	Hold-out	n/a	80/20	Prospective Observational	"Yes, on frame level"	30	1	10040 frames	unclear	5983	unclear	4057	Precision, Recall, F1-Score, DSC, mAP	Over sample-level averages of n cross-validation folds	no	Qualitative results	No	16.67 ms (60 FPS)	None	None	none	none
Nakanuma, 2023 [110]	Hold-out	n/a	unclear	Prospective Interventional	"Yes, on patient and frame level"	10	1	unclear	unclear	unclear	unclear	unclear	DSC, Others	Unclear	no	Qualitative results	No	0.09s time lag	None	None	none	"Frequentist non-parametric (e.g., Mann-Whitney)"
Namazi, 2022 [111]	Hold-out	n/a	unclear	Internal Retrospective	"Yes, on frame level", "Yes, on patient and frame level"	80	1	95 videos	50	unclear	45	unclear	Accuracy, F1-Score, Precision, Recall	Unclear	no	Class activation map	No	n/a	None	None	none	none
Nema , 2023 [112]	Hold-out	n/a	67/33	Internal Retrospective	"Yes, on frame level"	6	1	9195 frames	4	4560	2	4635	IoU, Others, Specificity	Over n frames in test set	no	Qualitative results	No	n/a	None	None	none	none
Nwoye, 2024 [113]	Hold-out	n/a	50/10/40	Internal Retrospective	"Yes, on patient and frame level"	20	1	35000 frames	unclear	unclear	unclear	unclear	Others	Unclear	„consistency accuracy metric	Qualitative results	No	45828	None	None	none	none
Nwoye, 2019 [114]	Hold-out	n/a	50/12.5/37,5	Internal Retrospective	"Yes, on patient and frame level"	80	1	80 videos	50	unclear	30	unclear	Others	Unclear	no	Qualitative results, Heat map	No	25 FPS	Other	Exact numbers	Unclear	none
Oh, 2024 [115]	Cross-Validation	5	24/6/5	Prospective Observational	"Yes, on patient and frame level"	35	1	300 frames	30	300	5	50	Recall, Precision, DSC	Over frame-level averages of n cross-validation folds	no	Qualitative results, Heat map	Fluorescence Imaging	15.3 FPS	SD	Exact numbers	Over frame-level averages of n cross-validation folds	none
Owen, 2022 [116]	Hold-out	n/a	60/20/20	Internal Retrospective	"Yes, on patient and frame level"	232	unclear	6732 frames	150	6122	15	610	IoU, Precision, Recall, F1-Score	Over n frames in test set	no	Qualitative results	No	n/a	None	None	none	none
Ozbulak, 2025 [117]	Others	n/a	n/a	Internal Retrospective	No	6	1	3600 frames	n/a	n/a	6	unclear	IoU	Other	no	no	No	25 FPS	None	None	none	none

Pan, 2023 [118]	Hold-out	n/a	unclear	Internal Retrospective	"Yes, on frame level"	36	2	4468 frames	unclear	unclear	unclear	unclear	mAP, Precision, Recall	Unclear	no	Qualitative results	No	n/a	None	None	none	none
Penza, 2018 [119]	Others	n/a	n/a	Internal Retrospective	Unclear	unclear	1	unclear	unclear	unclear	unclear	unclear	Precision, Recall, F1-Score	Unclear	no	Qualitative results	No	1.6 FPS	None	None	none	none
Pradeep, 2022 [120]	Hold-out	n/a	unclear	Internal Retrospective	Unclear	80	1	80 videos	unclear	2025	unclear	unclear	mAP	Unclear	no	Qualitative results	No	n/a	None	None	none	none
Prokopetc, 2015 [121]	Cross-Validation	7	unclear	Internal Retrospective	"Yes, on patient and frame level"	38	unclear	195 frames	unclear	unclear	unclear	unclear	Precision, Recall	Unclear	no	Qualitative results	No	n/a	SD	Exact numbers	Unclear	none
Protserov, 2024 [122]	Hold-out	n/a	70/15/15 + 2. Dataset for testing again	Prospective Observational	"Yes, on patient and frame level"	314	143	2674 frames	unclear	unclear	unclear	unclear	Precision, Recall, DSC	Over n frames in test set	no	Qualitative results, Heat map	No	70 ms	SD	Exact numbers	Over n frames in test set	none
Rahbar, 2020 [123]	Hold-out	n/a	unclear	Internal Retrospective	"Yes, on patient and frame level"	15	unclear	unclear	5	unclear	10	unclear	Others	"Over n independent samples (i.e., one patient/video) in independent test set"	no	others	No	0.1245 milliseconds per frame	SD, Other	Exact numbers	"Over n independent samples (i.e., one patient/video) in independent test set"	none
Rahbar, 2024 [124]	unclear	n/a	unclear	Internal Retrospective	Unclear	unclear	unclear	unclear	unclear	unclear	unclear	unclear	mIoU, mDSC	Over n frames in test set	no	Qualitative results	No	30.2 ms	None	None	none	none
Raja, 2024 [125]	Hold-out	n/a	unclear	Internal Retrospective	Unclear	10	1	2811 frames	unclear	2248	unclear	563	Precision, Recall, mAP	Over n frames in test set	no	Qualitative results	No	2.5 ms	None	None	none	none
Ryu, 2024 [126]	Cross-Validation	5	unclear	Internal Retrospective	"Yes, on frame level"	104	1	2624 frames	unclear	unclear	unclear	unclear	DSC, Precision, Recall	Unclear	no	Qualitative results	No	80 ms	None	None	none	none
Sahu, 2017 [127]	Hold-out	n/a	unclear	Internal Retrospective	"Yes, on patient and frame level"	15	1	15 videos	10	unclear	5	unclear	AP, mAP	Over n frames in test set	no	no	No	n/a	None	None	none	none
Samuel, 2021 [128]	unclear	n/a	unclear	Internal Retrospective	"Yes, on patient and frame level"	80	1	unclear	unclear	unclear	unclear	10383	Recall, Precision, F1-Score	Over n frames in test set	no	no	No	25 ms	None	None	none	none
Sanchez-Matilla, 2022 [129]	Cross-Validation	5	unclear	Internal Retrospective	"Yes, on patient and frame level"	17	1	8080 frames	14	unclear	3	unclear	Accuracy, F1-Score, mIoU, mDSC, Others	Over frame-level averages of n cross-validation folds	no	no	No	n/a	SD	Exact numbers	Over frame-level averages of n cross-validation folds	none
Satyanaik, 2024 [130]	Hold-out	n/a	60/20/20	Internal Retrospective	"Yes, on patient and frame level"	271	2	271 videos	216	unclear	55	unclear	mAP	Unclear	no	no	No	n/a	SD	Exact numbers	Unclear	none
Satyanaik, 2024 [131]	Hold-out	n/a	unclear	Internal Retrospective	"Yes, on patient and frame level"	271	2	18780 frames	216	unclear	55	unclear	mAP	Unclear	no	no	No	n/a	SD	Exact numbers	Unclear	none
Seenivasan, 2023 [132]	Hold-out	n/a	unclear	Internal Retrospective	"Yes, on patient and frame level"	unclear	2	unclear	unclear	unclear	unclear	unclear	Accuracy, Recall, F1-Score	Unclear	no	Qualitative results	No	n/a	None	None	none	none
Sengun, 2023 [133]	Cross-Validation	5	70/15/15	Internal Retrospective	"Yes, on patient and frame level"	40	1	2000 frames	34	1700	6	300	IoU, mDSC, PPV, Sensitivity	Unclear	no	Qualitative results	No	unclear	SD	Exact numbers	Unclear	none
Sengun, 2024 [134]	Cross-Validation	5	80/20/x	Internal Retrospective	"Yes, on patient and frame level"	20	1	1000 frames	16	800	4	200	Accuracy, Sensitivity, Specificity, mIoU, mDSC	Over frame-level averages of n cross-validation folds	no	Qualitative results, Probability Map	No	n/a	None	None	none	none
Shen, 2023 [135]	Hold-out	n/a	unclear	Internal Retrospective	"Yes, on frame level"	unclear	1	3000 frames	unclear	2250	unclear	750	mIoU, mDSC	Over n frames in test set	no	Qualitative results	No	9.78 ms	None	None	none	none
Sheng, 2024 [136]	unclear	n/a	unclear	Internal Retrospective	Unclear	17	1	8080 frames	unclear	unclear	unclear	unclear	mIoU	Unclear	no	others, Saliency Map	No	n/a	SD	Exact numbers	Unclear	none
Shi, 2020 [137]	Hold-out	n/a	unclear	Internal Retrospective	"Yes, on frame level"	80	1	4011 frames, Frames from Cholec80	unclear	3289	unclear	722	mAP	Over n frames in test set	no	Qualitative results	No	55.5 FPS	None	None	none	none
Shimgekar, 2021 [138]	Hold-out	n/a	unclear	Internal Retrospective	Unclear	unclear	unclear	242 frames	unclear	220	unclear	22	IoU	Unclear	no	Qualitative results	Other	n/a	None	None	none	none
Silva, 2022 [139]	Hold-out	n/a	unclear	Internal Retrospective	"Yes, on patient and frame level"	17	1	8080 frames	15	unclear	2	unclear	Precision, Recall, IoU, DSC	Over n frames in test set	no	Qualitative results	No	n/a	None	None	none	none
Smithmaitrie, 2024 [140]	Hold-out	n/a	unclear	Prospective Interventional	"Yes, on patient and frame level", "Yes, on frame level"	40	1	3200 frames	unclear	2560	unclear	640	AP, Precision, Recall	Over n frames in test set	no	Qualitative results	No	32.90 ms	None	None	none	none
Sonsilphong, 2022 [141]	Others	n/a	unclear	Prospective Interventional	"Yes, on patient and frame level"	5	unclear	unclear	5	unclear	cadaver	unclear	Accuracy	Unclear	no	Qualitative results	No	20 FPS	None	None	none	none
Streckert, 2023 [142]	Hold-out	n/a	unclear	Internal Retrospective	"Yes, on frame level"	6	1	300 frames	unclear	160	unclear	140	IoU	Over n frames in test set	no	Qualitative results	No	n/a	None	None	none	none
Strong, 2024 [143]	Hold-out	n/a	unclear	Internal Retrospective	"Yes, on frame level"	55	1	1383 frames	unclear	1234	unclear	149	IoU, Accuracy	Over n frames in test set	no	Qualitative results	No	n/a	SD	Exact numbers	Over n frames in test set	"Frequentist non-parametric (e.g., Mann-Whitney)"
Sun, 2022 [144]	Hold-out	n/a	unclear	Internal Retrospective	"Yes, on patient and frame level"	unclear	unclear	8000 frames	10	5000	10	3000	Accuracy	Unclear	no	Qualitative results	No	53 FPS	None	None	none	none

Takeuchi, 2023 [145]	Hold-out	n/a	81/19	Prospective Observational	"Yes, on patient and frame level"	160	1	2656 frames	130	2356	30	300	mAP, Accuracy, F1-Score, Sensitivity, Specificity, Precision	Over n frames in test set	no	Qualitative results	No	unclear	None	None	none	Other
Tao, 2023 [146]	Hold-out	n/a	unclear	Internal Retrospective	"Yes, on patient and frame level"	121	1	121 videos	67	unclear	54	unclear	mAP	Other	no	no	No	23.8 FPS	SD	Exact numbers	Other	none
Tokuyasu, 2021 [147]	Hold-out	n/a	unclear	Prospective Interventional	"Yes, on patient and frame level"	100	1	2339 frames	76	2145	23	194	AP	Unclear	no	Qualitative results	No	37.2 FPS	None	None	none	none
Urrea, 2024 [148]	Cross-Validation	unclear	unclear	Internal Retrospective	"Yes, on frame level"	17	1	8080 frames	unclear	6464	unclear	1616	Accuracy, IoU, DSC, F1-Score, Recall, Precision	Unclear	no	Qualitative results	No	56 ms	None	None	none	none
Valderrama, 2022 [149]	Cross-Validation	2	unclear	Internal Retrospective	"Yes, on patient and frame level"	8	1	2238 frames (bounding box)	unclear	unclear	unclear	unclear	mAP	Unclear	no	Qualitative results	No	n/a	SD	Exact numbers	Unclear	none
Vardazaryan, 2018 [150]	Hold-out	n/a	50/12.5/37,5	Internal Retrospective	"Yes, on patient and frame level"	80	1	80 videos	50	unclear	30	unclear	mAP	Unclear	no	Qualitative results, Localisation Map	No	unclear	None	None	none	none
Wagner, 2023 [151]	Hold-out	n/a	73/27	Internal Retrospective	"Yes, on patient and frame level"	33	3	33 videos	24	unclear	9	unclear	F1-Score	"Over n independent samples (i.e., one patient/video) in independent test set"	no	no	No	n/a	None	None	none	Other
Wang, 2023 [152]	Hold-out	n/a	unclear	Internal Retrospective	"Yes, on frame level"	20	1	2798 frames	unclear	2518	unclear	280	mAP, Precision, Recall	Over n frames in test set	no	Qualitative results	No	37.049 FPS	None	None	none	none
Wang, 2017 [153]	Cross-Validation	5	unclear	Internal Retrospective	"Yes, on patient and frame level"	15	1	15 Videos	unclear	unclear	unclear	unclear	mAP	Over n frames in test set	no	no	No	n/a	None	None	none	none
Wang, 2024 [154]	Hold-out	n/a	unclear	Internal Retrospective	"Yes, on patient and frame level", "Yes, on frame level"	95	1	187309 Keyframes	40 + unclear	88551	40 + unclear	98758	mAP	Over n frames in test set	no	no	No	n/a	None	None	none	none
Wang, 2023 [155]	Hold-out	n/a	80/20	Internal Retrospective	"Yes, on patient and frame level"	50	1	1372 frames	unclear	872	unclear	500	IoU, Accuracy	Over n frames in test set	no	Qualitative results	No	n/a	SD	Exact numbers, Graph	Over n frames in test set	none
Wang, 2025 [156]	Cross-Validation	2	60/40	Internal Retrospective	Unclear	13	1	unclear	8	unclear	5	unclear	IoU, AP, DSC, Others	Unclear	TSC (only for EV18)	Qualitative results	No	219 ms	None	None	none	none
Wang, 2021 [157]	Hold-out	n/a	unclear	Internal Retrospective	"Yes, on patient and frame level"	15	1	15 videos	10	2532	5	unclear	Recall, Precision, mAP	Over n frames in test set	no	Qualitative results	No	40.2 FPS	None	None	none	none
Wang, 2020 [158]	Hold-out	n/a	unclear	External Retrospective	"Yes, on frame level"	35	2	5696 frames	unclear	4557	unclear	1139	mAP	Unclear	no	Heat map	No	32.3 FPS	None	None	none	none
Wang, 2024 [159]	Hold-out	n/a	unclear	Internal Retrospective	"Yes, on patient and frame level"	81	2	81 videos	unclear	unclear	unclear	3645	IoU, AP	Over n frames in test set	no	Qualitative results, Class activation map	No	unclear	None	None	none	none
Wang, 2023 [160]	Hold-out	n/a	unclear	Internal Retrospective	"Yes, on frame level"	15	1	2532 frames	unclear	2026	unclear	506	mAP, Precision, Recall, F1-Score	Over n frames in test set	no	Qualitative results	No	50 FPS	None	None	none	none
Ward, 2022 [161]	Cross-Validation	10	90/x/10	Internal Retrospective	"Yes, on patient and frame level"	200	1	200 Videos	180	unclear	20	unclear	Others, Confusion Matrix	Unclear	no	others	No	n/a	Other	Exact numbers, Graph	Unclear	Unclear
Wei, 2024 [162]	Cross-Validation	unclear	80/20	Internal Retrospective	"Yes, on patient and frame level"	30	1	10040 frames	24	7160	6	2880	DSC, Others	Unclear	no	Qualitative results	No	n/a	None	None	none	none
Wei, 2023 [163]	Hold-out	n/a	unclear	Internal Retrospective	"Yes, on frame level"	30	1	30 videos	unclear	23600	unclear	4057	DSC, Others	Unclear	no	Qualitative results	No	n/a	None	None	none	none
Wu, 2024 [164]	Hold-out	n/a	unclear	Internal Retrospective	"Yes, on frame level"	21	1	1800 frames	unclear	1440	unclear	360	DSC, Accuracy, Specificity, Sensitivity	Over n frames in test set	no	Qualitative results	No	26.32 ms	None	None	none	none
Xi, 2022 [165]	Hold-out	n/a	unclear	Internal Retrospective	"Yes, on patient and frame level"	45	1	90489 frames	35	unclear	10	unclear	AP, mAP	"Over n independent samples (i.e., one patient/video) in independent test set"	no	Qualitative results	No	n/a	None	None	none	none
Xu, 2024 [166]	Hold-out	n/a	unclear	Internal Retrospective	"Yes, on patient and frame level"	22	1	11480 (incl. non-human)	17	4710	5	1138	IoU, DSC, Others	Over n frames in test set	no	Qualitative results, others	No	15.2 ms	SD	Exact numbers	Over n frames in test set	none
Xue, 2022 [167]	Hold-out	n/a	unclear	Internal Retrospective	"Yes, on frame level"	15	1	29533 frames	unclear	unclear	unclear	unclear	mAP	Over n frames in test set	no	Qualitative results	No	29.27 ms	None	None	none	none

Yamazaki, 2020 [168]	Hold-out	n/a	unclear	Internal Retrospective	"Yes, on patient and frame level"	62	1	13833 frames	52	10716	10	3117	IoU, Precision, Recall	Over n frames in test set	no	Qualitative results	No	n/a	None	None	none	none
Yamlahi, 2023 [169]	Cross-Validation	5	unclear	Internal Retrospective	"Yes, on patient and frame level"	45	1	90489 frames	40	unclear	5	unclear	Accuracy, mAP	Unclear	no	others	No	n/a	None	None	none	none
Yang, 2024 [170]	Hold-out	n/a	unclear	Internal Retrospective	"Yes, on frame level"	80	1	1510 frames	unclear	1208	unclear	302	Precision, Recall, mAP	Unclear	no	Qualitative results	No	unclear	None	None	none	none
Yang, 2023 [171]	Hold-out	n/a	unclear	Internal Retrospective	"Yes, on frame level"	17	1	8080 frames	unclear	6464	unclear	1616	DSC	Over n frames in test set	no	Qualitative results, Uncertainty Map	No	52 ms	SD, Other	Exact numbers, Graph	Over n frames in test set	none
Yang, 2021 [172]	Cross-Validation	3	unclear	Internal Retrospective	"Yes, on frame level"	67	1	67 videos	unclear	unclear	unclear	unclear	mDSC, Accuracy	Unclear	no	Heat map	No	n/a	None	None	None	none
Yin, 2024 [173]	Hold-out	n/a	80/20/x	Internal Retrospective	"Yes, on frame level"	unclear	1	unclear	unclear	unclear	unclear	unclear	Accuracy, AP	Over n frames in test set	no	no	No	n/a	None	None	none	none
Yuan, 2024 [174]	Hold-out	n/a	unclear	Internal Retrospective	"Yes, on patient and frame level"	45	1	unclear	40	unclear	5	unclear	Precision, Recall, DSC	Unclear	no	no	No	n/a	None	None	none	none
Zhang, 2024 [175]	Hold-out	n/a	85/15/x	Internal Retrospective	"Yes, on patient and frame level"	17	1	4298 frames	13	3246	4	1052	Accuracy, Others	Unclear	no	Qualitative results, Class activation map	No	n/a	SD	Exact numbers	Unclear	none
Zhang, 2023 [176]	Hold-out	n/a	unclear	Prospective Interventional	"Yes, on frame level"	unclear	1	2000 frames	unclear	1800	unclear	200	Precision, Recall, mAP	Over n frames in test set	no	Qualitative results	No	unclear	None	None	none	none
Zhang, 2023 [177]	Hold-out	n/a	50/50	Internal Retrospective	"Yes, on patient and frame level"	80	1	80 videos	40	unclear	40	unclear	mAP	"Over n independent samples (i.e., one patient/video) in independent test set"	no	others	No	n/a	SD	Exact numbers	"Over n independent samples (i.e., one patient/video) in independent test set"	"Frequentist non-parametric (e.g., Mann-Whitney)"
Zhang, 2020 [178]	Hold-out	n/a	unclear	External Retrospective	"Yes, on frame level"	35	2	5975 frames	unclear	4780	unclear	1195	mAP	Unclear	no	Qualitative results, Heat map	No	unclear	None	None	none	none
Zhang, 2023 [179]	unclear	unclear	67/17/16	Internal Retrospective	"Yes, on patient and frame level"	12	1	unclear	10	unclear	2	unclear	mAP	Unclear	no	Qualitative results	No	10-35 ms	Other	Exact numbers	Unclear	none
Zhang, 2020 [180]	Hold-out	n/a	unclear	Internal Retrospective	"Yes, on frame level"	15	1	1000 Frames	unclear	unclear	unclear	unclear	Sensitivity, DSC, Specificity, Others	Over n frames in test set	no	Qualitative results	No	15 ms	None	None	none	none
Zhao, 2021 [181]	Hold-out	n/a	unclear	External Retrospective	"Yes, on patient and frame level"	unclear	1	unclear	unclear	unclear	unclear	unclear	IoU, DSC	"Over n independent samples (i.e., one patient/video) in independent test set"	no	Qualitative results	No	300 ms	None	None	none	none
Zhao, 2023 [182]	Hold-out	n/a	unclear	Internal Retrospective	"Yes, on frame level"	15	1	5199 frames	unclear	4679	unclear	520	AP	Unclear	no	Qualitative results	No	68.5 FPS	None	None	none	none
Zhao, 2021 [183]	Hold-out	n/a	unclear	External Retrospective	"Yes, on patient and frame level"	8	1	unclear	unclear	unclear	unclear	unclear	IoU, DSC	"Over n independent samples (i.e., one patient/video) in independent test set"	no	Qualitative results	No	4 FPS	None	None	none	none
Zhao, 2019 [184]	Hold-out	n/a	unclear	External Retrospective, Internal Retrospective	Unclear	unclear	unclear	9376 frames	unclear	unclear	unclear	unclear	Accuracy	Unclear	no	Qualitative results	No	33.19	SD	Exact numbers	Unclear	none
Zheng, 2023 [185]	Hold-out	n/a	unclear	External Retrospective	"Yes, on patient and frame level"	132	2	1888 frames	unclear	1500	unclear	388	Precision, F1-Score, IoU	Unclear	no	Qualitative results	No	< 10 ms	None	None	none	Other
Zhou, 2024 [186]	Cross-Validation	5	unclear	Internal Retrospective	Unclear	55	1	531 frames	unclear	unclear	unclear	unclear	IoU, DSC	Unclear	no	Qualitative results, others	No	n/a	None	None	none	none
Zhou, 2022 [187]	Hold-out	n/a	67/33	Internal Retrospective	"Yes, on patient and frame level"	15	1	2532 frames	10	unclear	5	unclear	Precision, Recall, mAP	Unclear	no	Qualitative results	No	102.7 FPS	None	None	none	none
Zygomalas, 2024 [188]	Hold-out	n/a	unclear	Internal Retrospective	"Yes, on frame level"	25	unclear	1095 frames	unclear	800	unclear	295	F1-Score, mAP	Unclear	no	Qualitative results	No	n/a	None	None	none	none

Abbreviations: *n/a: not applicable, SD: standard deviation, CI: confidence interval, IQR: Interquartile Range*

Footnotes:

a. Data Split into Training/Validation/Testing: Only if specified based on the number of patients or videos per patient.
b. Indicates whether the model was evaluated on data from the same dataset as used for training (Internal Retrospective), on data from different institutions or datasets (External Retrospective), on prospectively collected data (Prospective Observational), or in clinical testing (Prospective Interventional).

c. Reported numbers of the data used throughout the entire study.

d. If the number of images was specified, it was reported as such; otherwise, the number of videos was provided.

e. Possible options for the calculation of performance metrics as well as model uncertainty and prediction variability: Over n independent samples (i.e., one patient/video) in independent test set (i.e., final models were tested on independent test set. Metrics were aggregated within one sample, if applicable (i.e., all frames of one video). Metrics/metric aggregates were aggregated over all samples in the test set)

- Over n independent samples (i.e., one patient/video) in independent test set (i.e., final models were tested on independent test set. Metrics were aggregated within one sample, if applicable (i.e., all frames of one video). Metrics/metric aggregates were aggregated over all samples in the test set)
- Over n independent samples across all cross-validation folds (i.e., models were cross-validated on CV folds split along patient/video lines, i.e., they are independent. Metrics were aggregated within one sample of the validation fold, if applicable (i.e., all frames of one video). This process was repeated for all CV folds and per-sample metrics/metric aggregates were aggregated over all samples across all CV folds)
- Over sample-level averages of n cross-validation folds (i.e., models were cross-validated on CV folds split along patient/video lines, i.e., they are independent. Metrics were aggregated within one sample of the validation fold, if applicable (i.e., all frames of one video). Then, per-sample metrics/metric aggregates were aggregated for each CV fold. The per-fold aggregates of n CV folds were aggregated for the final reported metric.)
- Over n frames in test set (i.e., final models were tested on test set. Metrics were calculated per frame and there were multiple frames per sample/patient/video in the test set, which were not aggregated. Per-frame metrics were aggregated over all frames in the test set, without consideration of “patient lines”)
- Over n frames across all cross-validation folds (i.e., models were cross-validated on CV folds. Metrics were calculated per frame and there were multiple frames per sample/patient/video in the test set, which were not aggregated. This process was repeated for all CV folds and per-frame metrics were aggregated over all individual frames across all CV folds)
- Over frame-level averages of n cross-validation folds (i.e., models were cross-validated on CV folds. Metrics were calculated per frame and there were multiple frames per sample/patient/video in the test set, which were not aggregated. Per-frame metrics were aggregated for each CV fold. The per-fold aggregates of n CV folds were aggregated for the final reported metric.)
- Other
- Unclear

f. Other statistical tests such as comparisons of model performance or with human expert annotations using frequentist parametric (e.g., t-test) or non-parametric methods.

Supplementary Table 5: Studies and study characteristics included in the systematic review and meta-analysis (3)

The following table presents an overview of all included studies, detailing key characteristics across four main categories: **Performance Metrics**. If the values were reported as average metrics for the most commonly used metrics in our review (not per every segment).

First author, year [reference]	IoU	DSC	Precision	Recall	mAP	Accuracy
Acharya, 2022 [1]	n/a	n/a	n/a	n/a	n/a	70/30 Hold-out: 96.09, 10-fold CV: 97.80
Al Hajj, 2018 [2]	n/a	n/a	n/a	n/a	97.89	n/a
Alapatt, 2021 [3]	61.58	n/a	n/a	n/a	n/a	95.22
Ali, 2022 [4]	n/a	n/a	n/a	n/a	42.64 (10% labelled data)	n/a
Alkhamaiseh, 2023 [5]	74.65	n/a	93.9	n/a	n/a	92
Alshirbaji, 2021 [6]	n/a	n/a	n/a	n/a	94.95	n/a
Alshirbaji, 2021 [7]	n/a	n/a	n/a	n/a	n/a	n/a
Alshirbaji, 2018 [8]	n/a	n/a	73.93	63.24	n/a	93.65
Ángeles-Cerón, 2021 [9]	n/a	31.3	n/a	n/a	n/a	n/a
Ángeles-Cerón, 2022 [10]	n/a	48.9	n/a	n/a	n/a	n/a
Aoyama, 2024 [11]	n/a	n/a	n/a	n/a	n/a	n/a
Arabian, 2022 [12]	n/a	n/a	n/a	n/a	88.4	n/a
Arabian, 2023 [13]	n/a	n/a	n/a	n/a	93.14	n/a
Aspart, 2022 [14]	n/a	n/a	n/a	n/a	n/a	n/a
Attia, 2017 [15]	82.7	n/a	n/a	n/a	n/a	93.3
Ayobi, 2024 [16]	87.05	n/a	n/a	n/a	n/a	n/a
Bai, 2023 [17]	n/a	n/a	n/a	n/a	n/a	n/a
Bakker, 2024 [18]	n/a	DSAD: 39.7 (All classes), Private: 73.5 (Catheter) and 75.5 (Urethra)	n/a	n/a	n/a	n/a
Bamba, 2021 [19]	n/a	n/a	93.4	91.3	n/a	n/a
Bamba, 2021 [20]	n/a	n/a	grasping forceps: 98, ultrasonic scalpel: 93.9, clip forceps: 92.7, angled forceps: 100, spatula forceps: 94.5	grasping forceps: 98.1, ultrasonic scalpel: 99.4, clip forceps: 96.2, angled forceps: 94.9, spatula forceps: 98.1	n/a	n/a
Ban, 2023 [21]	n/a	n/a	n/a	n/a	n/a	CVS100: 67.1, PGS200: 67.5, CholecT45: 30.6 (all triplets)
Batić, 2024 [22]	Semantic: 65.32	n/a	n/a	n/a	Action Triplet: 30.17	n/a
Batić, 2023 [23]	n/a	n/a	n/a	n/a	Action Triplet: 30.17	n/a
Boonkong, 2022 [24]	n/a	n/a	96.18	90.9	n/a	n/a
Brandenburg, 2023 [25]	n/a	n/a	n/a	n/a	n/a	n/a
Casella, 2021 [26]	n/a	71.76	89.76	50.66	n/a	n/a
Chen, 2024 [27]	n/a	n/a	n/a	n/a	n/a	n/a
Chen, 2013 [28]	n/a	n/a	n/a	n/a	n/a	n/a
Chen, 2017 [29]	n/a	n/a	n/a	n/a	n/a	93.2
Choi, 2017 [30]	n/a	n/a	n/a	n/a	72.26	n/a
Ciaparrone, 2020 [31]	n/a	n/a	87	n/a	n/a	n/a
Colleoni, 2024 [32]	LC: 84.5, Robotic Partial Nephrectomy: 66.35, Robotic Prostatectomy: 47.88	n/a	n/a	n/a	n/a	n/a
Colleoni, 2022 [33]	Tool Head: 38.99, Tool Shaft: 56.95	n/a	n/a	n/a	n/a	n/a
Daneshgar Rahbar, 2023 [34]	n/a	n/a	n/a	n/a	n/a	n/a
Davila, 2023 [35]	n/a	n/a	n/a	n/a	n/a	n/a
De Backer, 2023 [36]	94.4	n/a	n/a	n/a	n/a	n/a
den Boer, 2023 [37]	n/a	Azygos vein: 79, Aorta: 74, Lung: 89	n/a	n/a	n/a	n/a
Derathé, 2020 [38]	n/a	n/a	n/a	n/a	n/a	68
Du, 2019 [39]	n/a	n/a	n/a	n/a	n/a	n/a
Endo, 2023 [40]	n/a	n/a	n/a	n/a	n/a	n/a
Fernandez-Rodríguez, 2024 [41]	n/a	63.14	66.5	71.43	n/a	n/a
Fuentes-Hurtado, 2019 [42]	EndoVis15: 75.43, Sleeve Gastrectomy: 59	n/a	n/a	n/a	n/a	n/a
Fujinaga, 2023 [43]	n/a	Common Bile Duct: 45, Cystic Duct: 20, S4 Segment: 27, Rouvier Sulcus: 30	n/a	n/a	n/a	n/a
Ghamsarian, 2024 [44]	Endometriosis: 54.04, Colorectal: 60.01	Endometriosis: 66.37, Colorectal: 73.09	n/a	n/a	n/a	n/a
Gitau, 2024 [45]	n/a	n/a	n/a	n/a	n/a	n/a
Grammatikopoulou, 2023 [46]	LC: 69.38, PN: 62.91	n/a	n/a	n/a	n/a	n/a
Guédon, 2021 [47]	n/a	n/a	LC: 89, LH: 88	LC: 81, LH: 79	n/a	n/a
Hasan, 2021 [48]	n/a	89.56	n/a	n/a	n/a	n/a
Huang, 2022 [49]	74.92	85.63	n/a	n/a	n/a	n/a
Jalal, 2022 [50]	n/a	n/a	n/a	n/a	n/a	n/a
Jalal, 2023 [51]	n/a	n/a	n/a	n/a	95.6	n/a

Jamal, 2024 [52]	RARP: 85.8, LH: 78	n/a	n/a	n/a	n/a	n/a
Jang, 2023 [53]	n/a	n/a	n/a	n/a	n/a	n/a
Jaspers, 2024 [54]	n/a	n/a	n/a	n/a	n/a	n/a
Jearanai, 2023 [55]	n/a	n/a	n/a	n/a	n/a	n/a
Jha, 2021 [56]	81.83	87.39	93.01	88.99	n/a	98.98
Jin, 2020 [57]	n/a	n/a	n/a	n/a	89.2	n/a
Jin, 2018 [58]	n/a	n/a	n/a	n/a	63.1	n/a
Kamrul Hasan, 2021 [59]	n/a	n/a	n/a	n/a	n/a	n/a
Kanakatte, 2020 [60]	n/a	n/a	n/a	n/a	82	n/a
Kawamura, 2023 [61]	n/a	n/a	97.1	73.7	n/a	83.4
Khalid, 2023 [62]	n/a	n/a	n/a	n/a	n/a	n/a
Khalid, 2023 [63]	n/a	n/a	n/a	n/a	n/a	n/a
Kim, 2024 [64]	n/a	n/a	n/a	n/a	n/a	n/a
Kinoshita, 2024 [65]	29.2	44.2	n/a	n/a	n/a	n/a
Kitaguchi, 2023 [66]	n/a	Ureter: 72.2, Hypogastric Nerves: 57.9, Aortic Plexus: 62.8	Ureter: 70.7, Hypogastric Nerves: 59.2, Aortic Plexus: 65.6	Ureter: 73.9, Hypogastric Nerves: 56.6, Aortic Plexus: 60.3	n/a	n/a
Kitaguchi, 2022 [67]	n/a	n/a	n/a	n/a	91.9	n/a
Kitaguchi, 2022 [68]	n/a	n/a	n/a	n/a	n/a	n/a
Kletz, 2019 [69]	n/a	n/a	n/a	n/a	n/a	n/a
Kolbinger, 2023 [70]	n/a	n/a	n/a	n/a	n/a	n/a
Kolbinger, 2024 [71]	n/a	n/a	n/a	n/a	n/a	n/a
Kolbinger, 2024 [72]	n/a	n/a	n/a	n/a	n/a	n/a
Kondo, 2021 [73]	n/a	n/a	80.2	68	n/a	n/a
Konduri, 2024 [74]	n/a	n/a	n/a	n/a	n/a	LapGyn4: 99.74, Cholec80: 99.45, Heichole: 99.34
Kong, 2021 [75]	40.46	48.42	71.34	49.03	n/a	n/a
Kumazu, 2021 [76]	n/a	54.9	n/a	60.6	n/a	n/a
Kumazu, 2025 [77]	n/a	46	n/a	53	n/a	n/a
Labrunie, 2022 [78]	n/a	n/a	n/a	n/a	n/a	n/a
Lam, 2022 [79]	n/a	n/a	n/a	n/a	83.9	n/a
Laplante, 2022 [80]	n/a	n/a	n/a	n/a	n/a	Go: 92, No-Go: 92
Lavanchy, 2021 [81]	n/a	n/a	n/a	n/a	n/a	87
Le, 2023 [82]	n/a	n/a	96.9	93.7	n/a	n/a
Lee, 2024 [83]	n/a	n/a	n/a	n/a	n/a	n/a
Leifman, 2024 [84]	n/a	n/a	n/a	n/a	n/a	n/a
Leifmann, 2022 [85]	n/a	n/a	n/a	n/a	n/a	n/a
Li, 2025 [86]	n/a	n/a	n/a	n/a	n/a	n/a
Li, 2023 [87]	n/a	n/a	n/a	n/a	n/a	CVS: 92
Liao, 2024 [88]	n/a	n/a	n/a	n/a	n/a	n/a
Lin, 2024 [89]	n/a	n/a	n/a	n/a	n/a	n/a
Lin, 2021 [90]	73.5	81	n/a	n/a	n/a	n/a
Liu, 2022 [91]	n/a	n/a	n/a	n/a	71	n/a
Liu, 2024 [92]	48.2	n/a	n/a	n/a	n/a	n/a
Liu, 2022 [93]	n/a	n/a	n/a	n/a	n/a	n/a
Lou, 2023 [94]	n/a	n/a	n/a	n/a	n/a	n/a
Loukas, 2020 [95]	n/a	n/a	n/a	n/a	n/a	n/a
Loza, 2024 [96]	n/a	n/a	n/a	n/a	n/a	n/a
Maack, 2024 [97]	n/a	n/a	n/a	n/a	n/a	n/a
Madad Zadeh, 2020 [98]	Uterus: 84.5, Ovaries: 29.6, Tools: 54.5	n/a	n/a	n/a	n/a	n/a
Madani, 2022 [99]	Go: 53, No-Go: 71, Liver: 86, Gallbladder: 72, Triangle: 65	n/a	n/a	n/a	n/a	n/a
Maqbool, 2020 [100]	46	n/a	66	55	n/a	n/a
Marullo, 2023 [101]	n/a	81.89	n/a	n/a	n/a	90.63
Mascagni, 2022 [102]	n/a	n/a	n/a	n/a	n/a	n/a
Matsumoto, 2024 [103]	n/a	83.7	n/a	n/a	n/a	n/a
Mehta, 2024 [104]	n/a	71.5	n/a	n/a	n/a	n/a
Mishra, 2017 [105]	n/a	n/a	n/a	n/a	n/a	88.75
Murali, 2024 [106]	45.02	57.6	n/a	n/a	n/a	n/a
Murali, 2023 [107]	n/a	n/a	n/a	n/a	69.7	n/a
Murali, 2022 [108]	n/a	n/a	n/a	n/a	n/a	n/a
Myo, 2024 [109]	n/a	n/a	n/a	n/a	n/a	n/a
Nakanuma, 2023 [110]	n/a	n/a	n/a	n/a	n/a	n/a
Namazi, 2022 [111]	n/a	n/a	n/a	n/a	n/a	m2cai: 81.84, Cholec80: 91.92
Nema , 2023 [112]	80.89	n/a	n/a	n/a	n/a	n/a
Nwoye, 2024 [113]	n/a	n/a	n/a	n/a	n/a	n/a
Nwoye, 2019 [114]	n/a	n/a	n/a	n/a	n/a	n/a
Oh, 2024 [115]	n/a	BD: 72.8, AW: 42.9	n/a	n/a	n/a	n/a
Owen, 2022 [116]	68.1	n/a	86.7	76.1	n/a	n/a
Ozbulak, 2025 [117]	n/a	n/a	n/a	n/a	n/a	n/a
Pan, 2023 [118]	n/a	n/a	n/a	n/a	m2cai: 96.8, LGIL: 95.6	n/a
Penza, 2018 [119]	n/a	n/a	n/a	n/a	n/a	n/a
Pradeep, 2022 [120]	n/a	n/a	n/a	n/a	97.51	n/a
Prokopetc, 2015 [121]	n/a	n/a	n/a	Left FU: 73, Right FU: 64	n/a	n/a

Protserov, 2024 [122]	n/a	n/a	n/a	n/a	n/a	n/a
Rahbar, 2020 [123]	n/a	n/a	n/a	n/a	n/a	n/a
Rahbar, 2024 [124]	n/a	n/a	n/a	n/a	n/a	85.5
Raja, 2024 [125]	n/a	n/a	n/a	n/a	n/a	n/a
Ryu, 2024 [126]	n/a	n/a	n/a	n/a	n/a	n/a
Sahu, 2017 [127]	n/a	n/a	n/a	n/a	65	n/a
Samuel, 2021 [128]	n/a	n/a	91.5	78.4	n/a	n/a
Sanchez-Matilla, 2022 [129]	38.4	49.33	n/a	n/a	72.67	n/a
Satyanaik, 2024 [130]	n/a	n/a	n/a	n/a	n/a	n/a
Satyanaik, 2024 [131]	n/a	n/a	n/a	n/a	n/a	n/a
Seenivasan, 2023 [132]	n/a	n/a	n/a	n/a	n/a	n/a
Sengun, 2023 [133]	66	77	n/a	n/a	n/a	n/a
Sengun, 2024 [134]	63.7	77.1	n/a	n/a	n/a	n/a
Shen, 2023 [135]	63.37	75.48	n/a	n/a	n/a	n/a
Sheng, 2024 [136]	46.31	n/a	n/a	n/a	n/a	n/a
Shi, 2020 [137]	n/a	n/a	n/a	n/a	91.65	n/a
Shimgekar, 2021 [138]	n/a	n/a	n/a	n/a	n/a	n/a
Silva, 2022 [139]	55	n/a	73	72	n/a	n/a
Smithmaitrie, 2024 [140]	n/a	n/a	89.8	91.7	95.8	n/a
Sonsilphong, 2022 [141]	n/a	n/a	n/a	n/a	n/a	n/a
Streckert, 2023 [142]	76.15	n/a	n/a	n/a	n/a	n/a
Strong, 2024 [143]	82	n/a	n/a	n/a	n/a	87.2
Sun, 2022 [144]	n/a	n/a	n/a	n/a	n/a	n/a
Takeuchi, 2023 [145]	n/a	n/a	n/a	n/a	n/a	77.1
Tao, 2023 [146]	n/a	n/a	n/a	n/a	Cholec80: 95.15, m2cai: 93.4	n/a
Tokuyasu, 2021 [147]	n/a	n/a	Common bile duct: 32, Cystic duct: 74, Lower edge of the left medial liver segment: 31.4, Rouviere's sulcus: 10.1	n/a	n/a	n/a
Urrea, 2024 [148]	97.7	n/a	n/a	n/a	n/a	97.6
Valderrama, 2022 [149]	n/a	n/a	n/a	n/a	n/a	n/a
Vardazaryan, 2018 [150]	n/a	n/a	n/a	n/a	87.4	n/a
Wagner, 2023 [151]	n/a	n/a	n/a	n/a	n/a	n/a
Wang, 2023 [152]	n/a	n/a	97.4	98.1	n/a	n/a
Wang, 2017 [153]	n/a	n/a	n/a	n/a	n/a	n/a
Wang, 2024 [154]	n/a	n/a	n/a	n/a	n/a	n/a
Wang, 2023 [155]	n/a	n/a	n/a	n/a	n/a	n/a
Wang, 2025 [156]	n/a	GRASP: 88.72	n/a	n/a	n/a	n/a
Wang, 2021 [157]	n/a	n/a	n/a	n/a	87.6	n/a
Wang, 2020 [158]	n/a	n/a	n/a	n/a	m2cai: 70.1, AJU: 77.3	n/a
Wang, 2024 [159]	n/a	n/a	n/a	n/a	n/a	n/a
Wang, 2023 [160]	n/a	n/a	97.8	n/a	n/a	n/a
Ward, 2022 [161]	n/a	n/a	n/a	n/a	n/a	n/a
Wei, 2024 [162]	Robust-Mis: 86.6, CholecSeg8k: 70.2	Robust-Mis: 92.9, CholecSeg8k: 86.5	n/a	n/a	n/a	n/a
Wei, 2023 [163]	n/a	n/a	n/a	n/a	n/a	n/a
Wu, 2024 [164]	n/a	92.33	n/a	n/a	n/a	n/a
Xi, 2022 [165]	n/a	n/a	67.01	n/a	n/a	n/a
Xu, 2024 [166]	80.26	87.37	n/a	n/a	n/a	n/a
Xue, 2022 [167]	n/a	n/a	n/a	n/a	85.43	n/a
Yamazaki, 2020 [168]	n/a	n/a	87	83	n/a	n/a
Yamlahi, 2023 [169]	n/a	n/a	n/a	n/a	37.4	74
Yang, 2024 [170]	n/a	n/a	n/a	n/a	69.34	n/a
Yang, 2023 [171]	n/a	n/a	n/a	n/a	n/a	n/a
Yang, 2021 [172]	n/a	n/a	n/a	n/a	n/a	n/a
Yin, 2024 [173]	n/a	n/a	n/a	n/a	n/a	n/a
Yuan, 2024 [174]	n/a	n/a	n/a	n/a	n/a	n/a
Zhang, 2024 [175]	n/a	n/a	n/a	10.59	n/a	77.1
Zhang, 2023 [176]	n/a	n/a	n/a	95	95	n/a
Zhang, 2023 [177]	n/a	n/a	n/a	n/a	96.23	n/a
Zhang, 2020 [178]	n/a	n/a	n/a	n/a	m2cai: 69.6, AJU: 76.5	n/a
Zhang, 2023 [179]	n/a	n/a	n/a	n/a	n/a	n/a
Zhang, 2020 [180]	n/a	n/a	n/a	n/a	n/a	n/a
Zhao, 2021 [181]	80.1	89.5	n/a	n/a	n/a	n/a
Zhao, 2023 [182]	n/a	n/a	98.9	n/a	n/a	n/a
Zhao, 2021 [183]	77.7	86.2	n/a	n/a	n/a	n/a
Zhao, 2019 [184]	n/a	n/a	n/a	n/a	n/a	n/a
Zheng, 2023 [185]	82.8	n/a	91.35	n/a	n/a	n/a
Zhou, 2024 [186]	72.6	84.1	n/a	n/a	n/a	n/a
Zhou, 2022 [187]	n/a	n/a	96.3	92.3	n/a	n/a
Zygomalas, 2024 [188]	n/a	n/a	n/a	n/a	n/a	n/a

n/a: not applicable

Supplementary Figure 1: Overview of Findings by Category

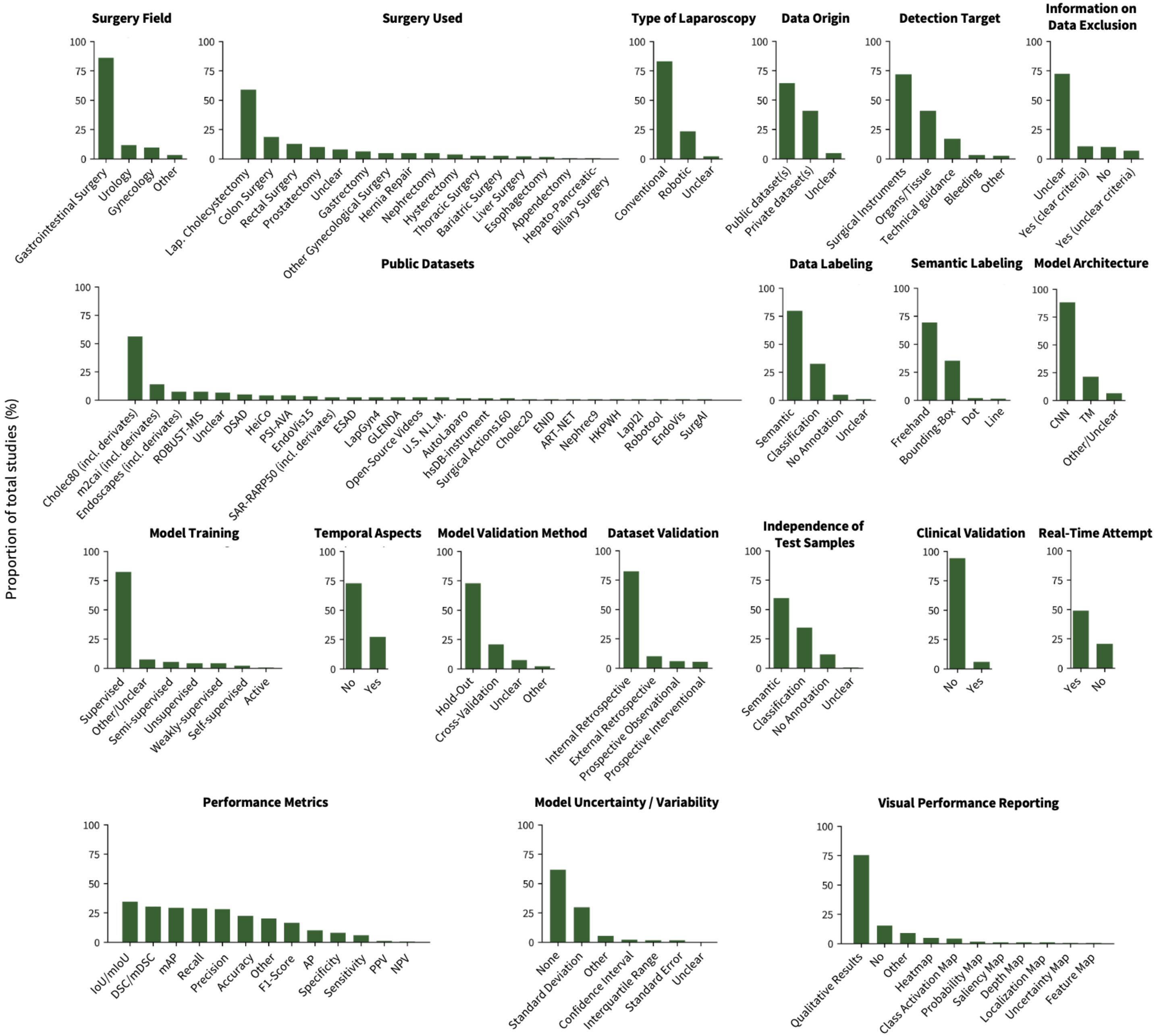

Bar charts display the proportion of individual data points within specific categories, based on all 188 included publications.

Supplementary Table 6: Clinical Translation

All 11 studies that tested the developed AI application in real-life scenarios on patients.

First author, year [reference]	Surgery	Number of patients	Type of AI implementation in the OR
Nakanuma, 2023 [110]	Laparoscopic Cholecystectomy	10	AI-based landmark detection and its performance evaluation clinically using a novel expert-based assessment method to verify its feasibility and accuracy in real surgical settings.
Fujinaga, 2023 [43]	Laparoscopic Cholecystectomy	10	A cross-AI system combining landmark detection and surgical phase recognition was developed to support surgeons in identifying key anatomical structures, potentially helping to prevent bile duct injury.
Smithmaitrie, 2024 [140]	Laparoscopic Cholecystectomy	unclear	AI application for detecting key anatomical landmarks and generating a real-time guided dissection line, supporting safer and more precise surgical navigation to help prevent bile duct injury.
Tokuyasu, 2021 [147]	Laparoscopic Cholecystectomy	1	AI–based system was developed to detect four key anatomical landmarks during laparoscopic cholecystectomy, aiming to raise intraoperative awareness and reduce the risk of bile duct injury.
Leifman, 2024 [84]	Laparoscopic Cholecystectomy	40	AI-based assessment of the achievement of the Critical View of Safety, demonstrating high sensitivity and specificity, for improving surgical safety and outcomes.
Brandenburg, 2023 [25]	Robotic Esophagectomy	unclear	AI-based recognition of key surgical features related to bleeding from intraoperative videos, aiming to personalize surgical outcome predictions.
Aoyama, 2024 [11]	Laparoscopic Gastrectomy	10	AI-based real-time navigation system that identifies specific anatomical landmarks linked to postoperative pancreatic fistula, helping surgeons reduce complication risks by highlighting critical structures during surgery.
Kitaguchi, 2023 [66]	Laparoscopic Left-sided colorectal resection	20	AI-based real-time recognition of critical anatomical structures, specifically the ureter and autonomic nerves, achieving faster and accurate identification compared to surgeons as a decision-support tool.
Zheng, 2023 [185]	Laparoscopic Partial nephrectomy	12	AI-based real-time system that evaluates surgeons' laparoscope control during surgery, helping non-experts reaching higher expert-level performance in visual field management.
De Backer, 2023 [36]	Different robotic kidney surgeries	10	AI-based detecting surgical instruments in real time during AR-guided robotic kidney surgeries, aiming to enhance safety by preventing hazardous overlay issues and enabling more reliable augmented reality integration in clinical settings.
Jearanai, 2023 [55]	Different laparoscopic surgeries	Unclear	AI model with a real-time alarm system to accurately detect abdominal wall layers during trocar insertion, aiming to enhance surgical safety by reducing complications through timely anatomical recognition and feedback.

Supplementary Table 7: Reporting Quality

All reporting quality ratings by category and publication. The following coding is used:

- Reported = 3
- Partially Reported = 2
- Reference to another publication = 1
- Not Reported = 0
- Not applicable = NA

Each cell shows three values (e.g., 3/2/2), representing the ratings from Annotator 1, Annotator 2, and the final consensus rating by Annotator 3, which was used for statistical analysis. Row 1 shows the interrater reliability between Annotator 1 and Annotator 2, assessed using Cohen's Kappa for each of the 18 reporting quality categories.

	Clinical Rationale					Data				Model Training and Validation						Critical appraisal		Ethics and Reproducibility	
**First author, year [reference]**	**Study Design**	**Prediction Problem**	**Clinical Problem**	**Clinical Goal**	**Existing AI**	**Data Sources**	**Data Selection**	**Data Preprocessing**	**Labeling**	**Model Type**	**Model Development**	**Model Validation**	**Model Performance**	**Statistical Methods**	**Performance Errors**	**Clinical Implications**	**Limitations**	**Ethical Statement**	**Code Publication**
**Cohen's Kappa Value**	**0.854**	**0.946**	**0.907**	**0.843**	**0.725**	**0.94**	**0.95**	**0.884**	**0.947**	**1**	**0.854**	**0.814**	**0.883**	**0.821**	**0.932**	**0.939**	**0.938**	**0.975**	**0.954**
Acharya, 2022 [1]	3/3/3	2/2/2	0/0/0	0/0/0	2/2/2	1/1/1	0/NA/0	1/1/1	1/1/1	3/3/3	2/2/2	2/2/2	2/2/2	2/2/2	2/3/2	0/0/0	0/0/0	NA/NA/NA	0/0/0
Al Hajj, 2018 [2]	3/3/3	3/3/3	0/0/0	0/0/0	3/3/3	1/1/1	0/0/0	2/2/2	1/1/1	3/3/3	3/3/3	2/2/2	3/3/3	3/3/3	3/3/3	0/0/0	2/2/2	NA/NA/NA	2/2/2
Alapatt, 2021 [3]	3/3/3	2/2/2	0/0/0	0/0/0	2/2/2	2/2/2	0/0/0	2/2/2	0/0/0	3/3/3	2/2/2	2/2/2	2/2/2	2/2/2	0/0/0	0/0/0	0/0/0	3/3/3	0/0/0
Ali, 2022 [4]	3/3/3	3/3/3	2/2/2	2/2/2	3/3/3	1/1/1	1/1/1	2/2/2	1/1/1	3/3/3	3/3/3	3/3/3	3/3/3	3/3/3	2/2/2	0/0/0	0/0/0	NA/NA/NA	3/3/3
Alkhamaiseh, 2023 [5]	3/3/3	3/3/3	3/3/3	3/3/3	2/2/2	2/2/2	2/2/2	2/2/2	3/3/3	3/3/3	2/2/2	3/3/3	2/2/2	2/2/2	0/0/0	3/3/3	3/3/3	0/0/0	0/0/0
Alshirbaji, 2021 [6]	3/3/3	3/3/3	0/0/0	0/0/0	3/3/3	2/2/2	1/1/1	2/2/2	1/1/1	3/3/3	3/3/3	3/3/3	2/2/2	2/2/2	2/2/2	0/0/0	2/2/2	NA/NA/NA	0/0/0
Alshirbaji, 2021 [7]	3/3/3	2/2/2	0/0/0	0/0/0	2/3/2	1/1/1	0/0/0	0/0/0	1/1/1	3/3/3	0/0/0	2/2/2	2/2/2	0/0/0	0/0/0	0/0/0	2/2/2	3/3/3	0/0/0
Alshirbaji, 2018 [8]	3/3/3	2/2/2	0/2/0	0/2/0	2/3/2	1/1/1	0/0/0	0/0/0	1/1/1	3/3/3	2/2/2	2/3/2	2/2/2	0/0/0	0/0/0	0/0/0	0/2/0	NA/NA/NA	0/0/0
Ángeles-Cerón, 2021 [9]	3/3/3	2/2/2	0/0/0	0/0/0	2/2/2	1/1/1	2/2/2	2/3/3	1/1/1	3/3/3	2/2/2	2/2/2	2/2/2	2/2/2	0/0/0	0/0/0	2/2/2	NA/NA/NA	0/0/0
Ángeles-Cerón, 2022 [10]	3/3/3	3/3/3	2/2/2	2/2/2	2/2/2	1/1/1	2/2/2	3/3/3	1/1/1	3/3/3	3/3/3	3/3/3	3/3/3	2/2/2	0/0/0	2/2/2	0/0/0	NA/NA/NA	0/0/0
Aoyama, 2024 [11]	3/3/3	3/3/3	3/3/3	3/3/3	2/3/2	3/3/3	2/2/2	2/2/2	3/3/3	3/3/3	0/0/0	2/2/2	2/2/2	2/2/2	2/2/2	3/3/3	3/3/3	3/3/3	0/0/0
Arabian, 2022 [12]	3/3/3	3/3/3	0/0/0	0/0/0	2/2/2	1/1/1	0/0/0	0/0/0	1/1/1	3/3/3	2/3/2	2/2/2	2/2/2	0/0/0	0/0/0	0/0/0	0/0/0	NA/NA/NA	0/0/0
Arabian, 2023 [13]	3/3/3	3/3/3	2/2/2	2/2/2	3/3/3	1/1/1	0/0/0	2/2/2	1/1/1	3/3/3	2/2/2	3/3/3	2/2/2	2/2/2	0/0/0	0/0/0	0/0/0	NA/NA/NA	0/0/0
Aspart, 2022 [14]	3/3/3	3/3/3	3/3/3	3/3/3	2/2/2	2/2/2	2/2/2	2/2/2	3/3/3	3/3/3	3/3/3	3/3/3	3/3/3	2/3/2	2/2/2	3/3/3	2/2/2	3/3/3	0/0/0
Attia, 2017 [15]	3/3/3	2/2/2	0/0/0	0/2/0	2/2/2	1/1/1	0/0/0	2/2/2	1/1/1	3/3/3	2/3/2	1/1/1	2/2/2	0/3/2	0/2/2	0/2/0	0/3/0	NA/NA/NA	0/0/0
Ayobi, 2024 [16]	3/3/3	2/2/2	0/0/0	0/0/0	3/3/3	2/2/2	0/0/0	2/2/2	3/3/3	3/3/3	3/3/3	2/2/2	3/3/3	2/2/2	2/2/2	0/0/0	0/0/0	2/2/2	3/3/3
Bai, 2023 [17]	3/3/3	3/3/3	0/0/0	0/0/0	2/3/2	1/1/1	0/0/0	0/0/0	1/1/1	3/3/3	2/2/2	2/2/2	3/3/3	2/2/2	0/0/0	0/0/0	0/0/0	NA/NA/NA	3/3/3
Bakker, 2024 [18]	3/3/3	3/3/3	3/3/3	3/3/3	2/2/2	2/2/2	2/2/2	2/2/2	3/3/3	3/3/3	2/2/2	2/2/2	2/2/2	2/2/2	2/2/2	2/2/2	3/3/3	3/3/3	0/0/0
Bamba, 2021 [19]	3/3/3	2/3/3	0/0/0	0/0/0	2/2/2	2/2/2	0/0/0	0/0/0	0/0/0	3/3/3	0/0/0	2/2/2	2/2/2	0/0/0	0/0/0	2/2/2	3/3/3	3/3/3	0/0/0
Bamba, 2021 [20]	3/3/3	3/3/3	2/2/2	2/2/2	3/3/3	2/3/3	2/2/2	2/3/2	2/2/2	3/3/3	2/2/2	2/2/2	3/3/3	2/2/2	2/2/2	3/3/3	3/3/3	3/3/3	2/2/2
Ban, 2023 [21]	3/3/3	3/3/3	0/0/0	2/2/2	2/2/2	1/1/1	0/0/0	2/2/2	1/1/1	3/3/3	3/3/3	2/3/2	3/3/3	2/2/2	0/0/0	2/2/2	0/0/0	0/0/0	0/0/0
Batić, 2024 [22]	3/3/3	2/2/2	0/0/0	0/0/0	2/2/2	1/1/1	0/0/0	1/1/1	1/1/1	3/3/3	2/2/2	2/2/2	2/2/2	2/2/2	0/0/0	0/0/0	0/0/0	NA/NA/NA	3/3/3
Batić, 2023 [23]	3/3/3	2/2/2	0/0/0	0/0/0	2/2/2	1/1/1	0/0/0	2/2/2	0/1/0	3/3/3	0/0/0	2/2/2	2/2/2	0/0/0	2/2/2	0/0/0	2/2/2	NA/NA/NA	2/2/2
Boonkong, 2022 [24]	3/3/3	2/2/2	0/0/0	0/0/0	2/2/2	0/0/0	0/0/0	0/0/0	0/0/0	3/3/3	0/0/0	2/2/2	2/2/2	0/0/0	0/0/0	0/0/0	0/0/0	0/0/0	0/0/0
Brandenburg, 2023 [25]	3/3/3	3/3/3	2/2/2	2/2/2	2/2/2	3/3/3	3/3/3	2/2/2	3/3/3	3/3/3	2/2/2	2/2/2	3/3/3	3/3/3	3/3/3	3/3/3	3/3/3	3/3/3	3/3/3
Casella, 2021 [26]	3/3/3	3/3/3	2/2/2	2/3/3	2/2/2	1/1/1	2/2/2	3/3/3	1/1/1	3/3/3	2/2/2	2/2/2	2/2/2	2/2/2	2/2/2	2/2/2	3/3/3	NA/NA/NA	0/0/0
Chen, 2024 [27]	3/3/3	2/2/2	2/2/2	2/2/2	0/0/0	2/2/2	0/0/0	0/0/0	NA/NA/NA	3/3/3	2/2/2	2/2/2	2/2/2	2/2/2	0/0/0	2/2/2	0/0/0	0/0/0	0/0/0
Chen, 2013 [28]	3/3/3	2/2/2	0/0/0	0/0/0	2/2/2	0/0/0	0/0/0	0/0/0	0/0/0	3/3/3	2/2/2	0/0/0	2/2/2	0/0/0	0/0/0	0/0/0	0/0/0	0/0/0	0/0/0
Chen, 2017 [29]	3/3/3	3/3/3	2/2/2	2/2/2	2/2/2	2/2/2	0/0/0	3/3/3	2/2/2	3/3/3	3/3/3	2/2/2	2/2/2	2/2/2	3/3/3	0/0/0	0/0/0	3/3/3	3/3/3
Choi, 2017 [30]	3/3/3	2/2/2	0/0/0	0/0/0	2/2/2	1/1/1	0/0/0	2/2/2	0/0/0	3/3/3	2/3/2	2/2/2	2/2/2	0/0/0	3/3/3	0/2/0	2/2/2	NA/NA/NA	0/0/0
Ciaparrone, 2020 [31]	3/3/3	2/2/2	2/2/2	2/2/2	2/2/2	2/2/2	2/2/2	3/3/3	2/2/2	3/3/3	2/2/2	2/2/2	2/2/2	0/0/0	0/0/0	2/2/2	0/2/0	0/0/0	0/0/0
Colleoni, 2024 [32]	3/3/3	2/2/2	2/2/2	2/2/2	2/2/2	2/2/2	0/0/0	2/2/2	0/0/0	3/3/3	2/2/2	2/2/2	3/3/3	3/3/3	0/0/0	2/2/2	0/0/0	0/0/0	0/0/0
Colleoni, 2022 [33]	3/3/3	2/2/2	0/0/0	0/0/0	2/2/2	2/2/2	0/0/0	2/2/2	0/0/0	3/3/3	3/3/3	2/2/2	3/3/3	2/2/2	0/0/0	0/0/0	0/0/0	0/0/0	3/3/3
Daneshgar Rahbar, 2023 [34]	3/3/3	2/2/2	2/2/2	2/2/2	2/2/2	2/2/2	0/0/0	2/2/2	0/0/0	3/3/3	2/2/2	2/2/2	2/2/2	2/2/2	2/2/2	0/0/0	0/0/0	NA/NA/NA	3/3/3
Davila, 2023 [35]	3/3/3	2/2/2	0/0/0	0/0/0	2/2/2	1/1/1	0/0/0	2/2/2	1/0/1	3/3/3	2/2/2	2/2/2	2/2/2	0/0/0	0/0/0	0/0/0	0/0/0	NA/NA/NA	0/0/0
De Backer, 2023 [36]	3/3/3	3/3/3	3/3/3	3/3/3	2/2/2	2/2/2	0/0/0	2/2/2	2/2/2	2/2/2	0/2/2	2/0/2	2/2/2	2/2/2	0/0/0	3/3/3	2/2/2	0/0/0	0/0/0
den Boer, 2023 [37]	3/3/3	3/3/3	3/3/3	2/3/3	2/2/2	3/3/3	3/3/3	2/2/2	3/3/3	3/3/3	2/3/3	2/2/2	3/3/3	3/3/3	0/0/0	3/3/3	3/3/3	3/3/3	0/0/0
Derathé, 2020 [38]	3/3/3	3/3/3	2/2/2	2/2/2	2/2/2	2/2/2	0/0/0	2/2/2	3/3/3	3/3/3	2/2/2	2/3/3	2/2/2	0/0/0	0/0/0	2/2/2	0/0/0	3/3/3	0/0/0
Du, 2019 [39]	3/3/3	0/0/0	0/0/0	0/0/0	3/3/3	2/2/2	2/2/2	2/2/2	1/1/1	3/3/3	3/3/3	2/2/2	2/2/2	2/2/2	2/2/2	0/0/0	2/2/2	NA/NA/NA	3/3/3
Endo, 2023 [40]	3/3/3	3/3/3	2/2/2	2/2/2	2/2/2	2/2/2	2/2/2	2/2/2	2/2/2	3/3/3	2/2/2	2/2/2	0/0/0	0/0/0	0/0/0	3/3/3	3/3/3	3/3/3	0/0/0
Fernandez-Rodríguez, 2024 [41]	3/3/3	2/2/2	0/0/0	0/0/0	2/2/2	1/1/1	0/0/0	2/2/2	1/1/1	3/3/3	2/2/2	2/2/2	3/3/3	2/3/2	0/0/0	0/0/0	2/2/2	NA/NA/NA	0/0/0
Fuentes-Hurtado, 2019 [42]	3/3/3	3/3/3	2/2/2	0/0/0	2/2/2	2/2/2	0/0/0	2/2/2	2/2/2	3/3/3	3/3/3	2/2/2	2/2/2	2/2/2	0/0/0	0/0/0	2/2/2	NA/NA/NA	0/0/0
Fujinaga, 2023 [43]	3/3/3	3/3/3	3/3/3	3/3/3	3/3/3	3/3/3	2/2/2	2/2/2	2/2/2	3/3/3	2/2/2	2/2/2	2/2/2	2/2/2	0/0/0	3/3/3	3/3/3	3/3/3	0/0/0

Ghamsarian, 2024 [44]	3/3/3	2/2/2	0/0/0	0/0/0	0/0/0	2/2/2	0/0/0	2/2/2	0/0/0	3/3/3	2/2/2	2/2/2	2/2/2	2/2/2	0/0/0	0/0/0	0/0/0	NA/NA/NA	3/3/3
Gitau, 2024 [45]	3/3/3	2/3/3	2/2/2	0/0/0	2/2/2	2/2/2	0/0/0	2/2/2	1/1/1	3/3/3	2/2/2	2/2/2	2/2/2	2/2/2	0/0/0	0/0/0	0/0/0	0/0/0	0/0/0
Grammatikopoulou, 2023 [46]	3/3/3	3/3/3	0/0/0	0/0/0	2/2/2	2/3/3	0/0/0	0/0/0	0/0/0	3/3/3	2/2/2	2/2/2	2/2/2	2/2/2	0/0/0	0/0/0	0/0/0	2/2/2	0/0/0
Guédon, 2021 [47]	3/3/3	3/3/3	2/2/2	2/2/2	2/2/2	3/3/3	2/2/2	2/2/2	2/2/2	3/3/3	2/2/2	2/2/2	2/2/2	0/0/0	0/0/0	3/3/3	3/3/3	2/2/2	0/0/0
Hasan, 2021 [48]	3/3/3	0/2/2	0/2/2	0/2/2	2/3/2	1/1/1	0/0/0	2/2/2	1/1/1	3/3/3	2/2/2	2/2/2	2/3/2	2/2/2	0/0/0	0/2/2	0/0/0	NA/NA/NA	0/0/0
Huang, 2022 [49]	3/3/3	2/2/2	0/0/0	0/0/0	2/2/2	1/1/1	0/0/0	2/2/2	0/0/0	3/3/3	3/3/3	3/3/3	2/2/2	0/0/0	0/0/0	0/0/0	0/0/0	NA/NA/NA	0/0/0
Jalal, 2022 [50]	3/3/3	2/2/2	0/0/0	0/0/0	2/2/2	1/1/1	1/1/1	2/2/2	0/0/0	3/3/3	2/2/2	2/2/2	2/2/2	0/0/0	0/2/0	0/0/0	0/0/0	NA/NA/NA	0/0/0
Jalal, 2023 [51]	3/3/3	3/3/3	2/2/2	2/2/2	2/2/2	1/1/1	0/0/0	2/2/2	2/2/2	3/3/3	2/2/2	2/2/2	2/2/2	2/2/2	0/0/0	2/2/2	2/2/2	NA/NA/NA	2/2/2
Jamal, 2024 [52]	3/3/3	2/2/2	2/2/2	0/0/0	2/2/2	1/1/1	0/0/0	0/0/0	1/1/1	3/3/3	3/3/3	2/2/2	2/2/2	2/3/2	0/0/0	0/0/0	0/0/0	NA/NA/NA	0/0/0
Jang, 2023 [53]	2/2/2	0/0/0	0/0/0	0/0/0	2/2/2	0/0/0	0/0/0	0/0/0	0/0/0	3/3/3	0/0/0	2/2/2	2/2/2	0/0/0	2/2/2	0/0/0	0/0/0	0/0/0	0/0/0
Jaspers, 2024 [54]	3/3/3	2/2/2	0/0/0	0/0/0	2/2/2	1/1/1	0/0/0	2/2/2	1/1/1	3/3/3	2/2/2	2/2/2	2/2/2	2/2/2	0/0/0	0/0/0	0/0/0	NA/NA/NA	3/3/3
Jearanai, 2023 [55]	3/3/3	3/3/3	3/3/3	3/3/3	2/2/2	3/3/3	0/0/0	2/3/2	0/0/0	3/3/3	3/3/3	3/2/2	2/2/2	2/2/2	0/0/0	3/3/3	3/3/3	3/3/3	0/0/0
Jha, 2021 [56]	3/3/3	3/3/3	0/0/0	0/0/0	2/2/2	1/1/1	0/0/0	2/2/2	1/1/1	3/3/3	2/2/2	2/2/2	2/2/2	2/2/2	0/0/0	0/0/0	0/0/0	NA/NA/NA	0/0/0
Jin, 2020 [57]	3/3/3	3/3/3	2/2/2	2/2/2	3/3/3	1/1/1	0/0/0	2/2/2	1/1/1	3/3/3	3/3/3	2/2/2	3/3/3	2/2/2	2/2/2	0/0/0	0/0/0	0/0/0	3/3/3
Jin, 2018 [58]	3/3/3	3/3/3	2/2/2	2/2/2	2/2/2	1/1/1	0/0/0	2/2/2	2/2/2	3/3/3	2/2/2	2/2/2	2/2/2	0/0/0	0/0/0	2/2/2	0/0/0	NA/NA/NA	0/0/0
Kamrul Hasan, 2021 [59]	3/3/3	3/3/3	0/0/0	0/0/0	2/2/2	2/2/2	0/0/0	2/2/2	0/0/0	3/3/3	3/3/3	2/2/2	2/2/2	3/3/3	2/2/2	0/0/0	0/0/0	0/0/0	2/2/2
Kanakatte, 2020 [60]	3/2/2	2/3/3	0/2/2	0/2/2	2/3/2	1/1/1	0/0/0	2/3/2	2/2/2	3/3/3	2/3/2	2/2/2	2/2/2	0/2/2	0/0/0	0/0/0	0/0/0	NA/NA/NA	0/0/0
Kawamura, 2023 [61]	3/3/3	3/3/3	3/3/3	3/3/3	2/2/2	2/2/2	2/2/2	2/2/2	0/0/0	3/3/3	2/2/2	2/2/2	2/2/2	2/2/2	0/0/0	3/3/3	3/3/3	3/3/3	3/3/3
Khalid, 2023 [62]	3/3/3	2/2/2	2/2/2	0/0/0	2/2/2	1/1/1	0/0/0	2/2/2	2/2/2	3/3/3	3/3/3	2/2/2	2/2/2	0/0/0	0/0/0	0/0/0	2/2/2	0/0/0	3/3/3
Khalid, 2023 [63]	3/3/3	3/3/3	3/3/3	3/3/3	2/2/2	2/2/2	2/2/2	2/2/2	2/2/2	3/3/3	1/1/1	2/2/2	1/1/1	0/0/0	2/2/2	3/3/3	3/3/3	3/3/3	0/0/0
Kim, 2024 [64]	3/3/3	2/2/2	0/2/2	0/2/2	2/2/2	1/1/1	0/2/0	2/2/2	1/1/1	3/3/3	2/2/2	2/2/2	2/2/2	0/0/0	0/0/0	0/0/0	0/0/0	NA/NA/NA	0/0/0
Kinoshita, 2024 [65]	3/3/3	3/3/3	3/3/3	3/3/3	2/2/2	3/3/3	2/2/2	0/0/0	3/2/3	2/2/2	0/0/0	2/2/2	2/2/2	2/3/3	0/0/0	2/2/2	3/3/3	3/3/3	0/0/0
Kitaguchi, 2023 [66]	3/3/3	3/3/3	2/3/3	2/2/2	2/3/2	2/2/2	0/0/0	2/2/2	3/3/3	3/3/3	2/2/2	2/2/2	2/2/2	0/0/0	0/0/0	3/3/3	3/3/3	3/3/3	0/0/0
Kitaguchi, 2022 [67]	3/3/3	3/3/3	2/2/2	2/2/2	2/2/2	2/2/2	2/2/2	2/2/2	3/3/3	3/3/3	2/2/2	2/2/2	2/2/2	2/2/2	0/0/0	2/2/2	3/3/3	3/3/3	2/2/2
Kitaguchi, 2022 [68]	3/3/3	3/3/3	2/3/3	3/3/3	2/2/2	3/3/3	2/2/2	2/3/2	2/3/3	3/3/3	3/3/3	2/2/2	3/3/3	3/3/3	2/2/2	3/3/3	3/3/3	3/3/3	2/2/2
Kletz, 2019 [69]	3/3/3	2/2/2	2/2/2	2/2/2	2/2/2	2/2/2	0/0/0	2/2/2	0/0/0	3/3/3	2/2/2	2/2/2	2/2/2	2/2/2	0/0/0	2/2/2	0/0/0	0/0/0	0/0/0
Kolbinger, 2023 [70]	3/3/3	3/3/3	3/3/3	3/3/3	2/2/2	2/2/2	1/1/1	2/2/2	1/1/1	3/3/3	2/2/2	3/3/3	3/3/3	2/2/2	0/0/0	3/3/3	3/3/3	3/3/3	3/3/3
Kolbinger, 2024 [71]	3/3/3	3/3/3	3/3/3	3/3/3	2/2/2	3/3/3	3/3/3	3/3/3	3/3/3	3/3/3	2/2/2	2/2/2	2/2/2	2/2/2	0/0/0	2/2/2	3/3/3	3/0/3	3/0/3
Kolbinger, 2024 [72]	3/3/3	3/3/3	2/2/2	0/0/0	2/2/2	1/1/1	0/0/0	2/2/2	1/0/1	3/3/3	1/1/1	2/2/2	3/3/3	2/2/2	0/0/0	0/0/0	3/3/3	NA/NA/NA	0/0/0
Kondo, 2021 [73]	3/3/3	3/3/3	2/2/2	2/2/2	2/2/2	1/1/1	0/0/0	2/2/2	1/1/1	3/3/3	3/3/3	3/3/3	2/2/2	2/2/2	0/0/0	0/0/0	0/0/0	NA/NA/NA	0/0/0
Konduri, 2024 [74]	3/3/3	2/2/2	0/0/0	0/0/0	2/2/2	1/1/1	0/0/0	2/2/2	1/1/1	3/3/3	2/2/2	2/2/2	2/2/2	2/2/2	0/0/0	0/0/0	2/2/2	NA/NA/NA	0/0/0
Kong, 2021 [75]	3/3/3	3/3/3	2/2/2	2/2/2	2/2/2	2/2/2	0/0/0	3/3/3	3/3/3	3/3/3	3/3/3	3/3/3	3/3/3	2/2/2	0/2/0	0/0/0	0/0/0	NA/NA/NA	3/3/3
Kumazu, 2021 [76]	3/3/3	3/3/3	3/3/3	3/3/3	2/2/2	2/2/2	3/3/3	2/2/2	2/2/2	3/3/3	2/2/2	1/1/1	2/2/2	2/2/2	3/3/3	3/3/3	3/3/3	3/3/3	0/0/0
Kumazu, 2025 [77]	3/3/3	3/3/3	3/3/3	3/3/3	2/2/2	2/2/2	2/2/2	2/2/2	2/2/2	3/3/3	2/2/2	2/2/2	2/2/2	0/0/0	2/2/2	3/3/3	3/3/3	3/3/3	0/0/0
Labrunie, 2022 [78]	3/3/3	2/2/2	2/2/2	2/2/2	2/2/2	2/2/2	0/0/0	2/2/2	0/0/0	2/2/2	2/2/2	2/2/2	2/2/2	2/2/2	2/2/2	2/2/2	2/2/2	3/3/3	0/0/0
Lam, 2022 [79]	3/3/3	3/3/3	2/2/2	2/3/2	2/2/2	2/2/2	0/0/0	2/2/2	3/3/3	3/3/3	2/2/2	2/2/2	2/2/2	0/0/0	0/0/0	0/0/0	2/2/2	0/0/0	0/0/0
Laplante, 2022 [80]	3/3/3	3/3/3	3/3/3	2/2/2	2/2/2	2/2/2	3/3/3	0/0/0	3/3/3	1/1/1	0/0/0	1/1/1	2/2/2	0/0/0	0/0/0	3/3/3	2/2/2	0/0/0	1/1/1
Lavanchy, 2021 [81]	3/3/3	3/3/3	2/2/2	2/3/3	2/2/2	3/3/3	0/0/0	2/2/2	2/2/2	3/3/3	2/2/2	3/3/3	3/3/3	2/3/2	2/2/2	3/3/3	0/0/0	3/3/3	0/0/0
Le, 2023 [82]	3/3/3	2/2/2	0/0/0	0/0/0	2/2/2	1/1/1	0/0/0	2/2/2	1/1/1	3/3/3	2/2/2	2/2/2	2/2/2	2/2/2	0/0/0	0/0/0	0/0/0	NA/NA/NA	0/0/0
Lee, 2024 [83]	3/3/3	2/2/2	2/2/2	0/0/0	2/2/2	1/1/1	0/0/0	2/2/2	1/1/1	3/3/3	3/3/3	2/3/2	3/3/3	3/3/3	0/0/0	0/0/0	0/0/0	NA/NA/NA	0/0/0
Leifman, 2024 [84]	3/3/3	3/3/3	2/2/2	0/0/0	3/3/3	1/1/1	0/0/0	0/0/0	1/1/1	3/3/3	2/2/2	1/1/1	2/2/2	2/2/2	0/0/0	0/0/0	0/0/0	NA/NA/NA	0/0/0
Leifmann, 2022 [85]	3/3/3	3/3/3	3/3/3	3/3/3	2/2/2	3/3/3	2/2/2	2/2/2	3/3/3	3/3/3	2/2/2	2/2/2	3/3/3	2/2/2	2/3/2	3/3/3	3/3/3	3/3/3	0/0/0
Li, 2025 [86]	3/3/3	3/3/3	3/3/3	3/3/3	2/2/2	1/1/1	0/0/0	2/2/2	1/1/1	3/3/3	2/2/2	2/2/2	2/3/2	2/3/2	0/0/0	2/3/3	2/2/2	0/0/0	0/0/0
Li, 2023 [87]	3/3/3	3/3/3	2/2/2	0/0/0	2/2/2	1/1/1	0/0/0	0/0/0	1/1/1	3/3/3	2/2/2	2/3/3	2/3/3	0/0/0	0/0/0	2/2/2	0/0/0	NA/NA/NA	3/3/3
Liao, 2024 [88]	3/3/3	0/0/0	0/0/0	0/2/2	2/2/2	1/1/1	0/0/0	2/2/2	1/1/1	3/3/3	3/2/3	2/2/2	2/2/2	3/3/3	0/0/0	0/2/0	0/2/0	NA/NA/NA	0/0/0
Lin, 2024 [89]	3/3/3	2/2/2	0/0/0	0/0/0	2/2/2	1/1/1	0/0/0	2/2/2	1/1/1	3/3/3	3/3/3	2/2/2	2/2/2	2/2/2	2/2/2	0/0/0	0/0/0	NA/NA/NA	2/2/2
Lin, 2021 [90]	3/3/3	3/3/3	0/0/0	0/0/0	2/2/2	1/1/1	2/2/2	2/2/2	1/1/1	3/3/3	3/3/3	2/2/2	3/3/3	3/3/3	0/0/0	0/0/0	0/0/0	NA/NA/NA	0/0/0
Liu, 2022 [91]	3/3/3	3/3/3	3/3/3	3/3/3	3/3/3	3/3/3	3/3/3	2/2/2	3/3/3	3/3/3	2/2/2	3/3/3	3/3/3	3/3/3	2/2/2	3/3/3	3/3/3	3/3/3	0/0/0
Liu, 2024 [92]	3/3/3	3/3/3	2/2/2	2/2/2	2/2/2	2/2/2	2/2/2	2/2/2	0/0/0	3/3/3	3/3/3	2/2/2	3/3/3	2/3/3	0/0/0	0/0/0	0/0/0	0/0/0	3/3/3
Liu, 2022 [93]	3/3/3	2/2/2	2/2/2	2/2/2	2/2/2	1/1/1	0/0/0	2/2/2	2/2/2	3/3/3	3/3/3	2/2/2	2/2/2	2/2/2	2/2/2	0/0/0	2/2/2	NA/NA/NA	0/0/0
Lou, 2023 [94]	3/3/3	3/3/3	0/0/0	0/0/0	3/3/3	1/1/1	0/0/0	2/2/2	1/1/1	3/3/3	3/3/3	2/2/2	2/2/2	2/2/2	3/3/3	0/0/0	0/0/0	NA/NA/NA	0/0/0
Loukas, 2020 [95]	3/3/3	3/3/3	2/2/2	0/0/0	2/2/2	1/1/1	0/0/0	2/2/2	1/1/1	3/3/3	2/2/2	3/3/3	2/2/2	2/2/2	2/2/2	2/2/2	2/2/2	NA/NA/NA	0/0/0
Loza, 2024 [96]	3/3/3	3/3/3	2/2/2	2/2/2	2/2/2	1/1/1	0/0/0	2/2/2	1/1/1	3/3/3	3/2/2	2/2/2	2/2/2	2/2/2	0/0/0	2/2/2	0/0/0	NA/NA/NA	0/0/0
Maack, 2024 [97]	3/3/3	3/3/3	2/2/2	2/2/2	2/2/2	1/1/1	0/0/0	2/2/2	1/1/1	3/3/3	2/0/2	2/2/2	2/3/2	2/2/2	0/0/0	0/0/0	0/0/0	NA/NA/NA	3/3/3
Madad Zadeh, 2020 [98]	3/3/3	2/2/2	0/0/0	0/0/0	2/2/2	2/2/2	0/0/0	0/0/0	2/2/2	3/3/3	0/0/0	2/2/2	2/2/2	0/0/0	0/0/0	2/2/2	2/2/2	3/3/3	2/2/2
Madani, 2022 [99]	3/3/3	3/3/3	3/3/3	3/3/3	2/3/2	2/2/2	2/2/2	2/2/2	3/3/3	3/3/3	3/3/3	2/2/2	2/2/2	0/0/0	2/2/2	3/3/3	3/3/3	0/0/0	0/0/0
Maqbool, 2020 [100]	3/3/3	3/3/3	2/2/2	2/2/2	3/2/3	1/1/1	0/0/0	2/2/2	1/1/1	3/3/3	2/2/2	2/2/2	2/2/2	2/2/2	0/0/0	0/0/0	0/0/0	NA/NA/NA	3/3/3
Marullo, 2023 [101]	3/3/3	3/3/3	2/2/2	2/2/2	0/0/0	2/3/3	0/0/0	2/2/2	2/2/2	3/3/3	2/3/2	2/2/2	2/2/2	0/0/0	0/0/0	2/2/2	2/2/2	3/3/3	0/0/0
Mascagni, 2022 [102]	3/3/3	3/3/3	3/3/3	3/3/3	2/2/2	3/3/3	0/0/0	2/2/2	3/3/3	3/3/3	2/3/3	2/2/2	3/3/3	2/2/2	0/0/0	3/3/3	3/3/3	3/3/3	0/0/0
Matsumoto, 2024 [103]	3/3/3	3/3/3	3/3/3	2/2/2	2/2/2	2/2/2	0/0/0	2/2/2	0/0/0	2/2/2	0/0/0	2/2/2	0/0/0	0/0/0	0/0/0	3/3/3	3/3/3	3/3/3	3/3/3
Mehta, 2024 [104]	3/3/3	2/2/2	2/2/2	2/2/2	2/2/2	2/2/2	0/0/0	2/2/2	0/0/0	3/3/3	3/3/3	3/3/3	2/3/2	2/2/2	2/2/2	2/2/2	2/2/2	3/3/3	0/0/0
Mishra, 2017 [105]	3/3/3	2/2/2	2/2/2	0/0/0	0/0/0	1/1/1	0/0/0	2/2/2	1/1/1	3/3/3	3/3/3	2/2/2	2/2/2	0/0/0	2/2/2	0/0/0	0/0/0	NA/NA/NA	3/3/3
Murali, 2024 [106]	3/3/3	2/2/2	0/0/0	0/0/0	2/2/2	1/1/1	0/0/0	0/0/0	1/1/1	3/3/3	2/3/3	0/3/2	2/2/2	2/3/2	0/0/0	0/0/0	0/0/0	NA/NA/NA	0/0/0
Murali, 2023 [107]	3/3/3	2/2/2	0/0/0	0/0/0	2/2/2	1/1/1	0/0/0	0/0/0	1/1/1	3/3/3	3/2/2	2/2/2	2/2/2	2/2/2	0/0/0	0/0/0	0/0/0	NA/NA/NA	0/0/0
Murali, 2022 [108]	3/3/3	3/3/3	2/2/2	2/2/2	2/2/2	2/2/2	0/0/0	2/2/2	2/2/2	3/3/3	3/3/3	2/2/2	3/3/3	2/2/2	2/2/2	0/0/0	0/0/0	0/0/0	0/0/0
Myo, 2024 [109]	3/3/3	3/3/3	2/2/2	2/2/2	2/2/2	1/1/1	0/0/0	3/3/3	3/3/3	3/3/3	3/3/3	3/3/3	2/2/2	2/2/2	0/0/0	3/3/3	3/3/3	3/3/3	0/0/0
Nakanuma, 2023 [110]	3/3/3	3/3/3	3/3/3	3/3/3	2/3/3	3/3/3	0/0/0	2/2/2	3/3/3	3/3/3	2/2/2	3/3/3	2/2/2	2/2/2	3/3/3	3/3/3	3/3/3	3/3/3	0/0/0
Namazi, 2022 [111]	3/3/3	3/3/3	2/2/2	2/2/2	2/3/2	2/2/2	1/1/1	2/2/2	1/1/1	3/3/3	3/3/3	2/3/2	2/2/2	3/3/3	0/0/0	2/2/2	0/2/0	NA/NA/NA	1/1/1
Nema , 2023 [112]	3/3/3	2/2/2	2/2/2	0/0/0	3/3/3	1/1/1	2/2/2	0/0/0	1/1/1	3/3/3	3/3/3	2/2/2	2/2/2	0/0/0	0/0/0	0/0/0	0/0/0	NA/NA/NA	0/0/0
Nwoye, 2024 [113]	3/3/3	3/3/3	2/2/2	2/2/2	2/3/2	1/1/1	0/0/0	3/3/3	1/1/1	3/3/3	3/3/3	2/2/2	3/3/3	2/2/2	3/3/3	2/2/2	3/3/3	NA/NA/NA	2/2/2
Nwoye, 2019 [114]	3/3/3	3/3/3	0/0/0	0/0/0	2/2/2	1/1/1	0/0/0	2/2/2	0/0/0	3/3/3	2/2/2	2/2/2	2/2/2	0/0/0	2/2/2	0/0/0	0/0/0	NA/NA/NA	0/0/0
Oh, 2024 [115]	3/3/3	3/3/3	3/3/3	3/3/3	3/3/3	3/3/3	3/3/3	2/2/2	3/3/3	3/3/3	3/3/3	3/3/3	2/2/2	2/2/2	0/0/0	3/3/3	3/3/3	3/3/3	0/0/0
Owen, 2022 [116]	3/3/3	3/3/3	2/3/3	2/2/2	2/2/2	0/0/0	0/0/0	2/2/2	2/2/2	3/3/3	2/2/2	3/3/3	2/2/2	2/2/2	2/2/2	2/2/2	0/0/0	NA/NA/NA	0/0/0
Ozbulak, 2025 [117]	3/3/3	2/3/3	2/2/2	2/2/2	2/2/2	1/1/1	0/0/0	0/0/0	1/1/1	3/3/3	2/3/3	2/2/2	2/2/2	0/0/0	2/2/2	2/2/2	0/0/0	NA/NA/NA	0/0/0

Pan, 2023 [118]	3/3/3	3/3/3	2/2/2	2/3/3	2/2/2	2/2/2	0/0/0	2/2/2	0/0/0	3/3/3	3/3/3	2/2/2	2/2/2	2/2/2	0/0/0	0/0/0	0/0/0	NA/NA/NA	0/0/0
Penza, 2018 [119]	2/2/2	2/2/2	0/0/0	0/0/0	0/0/0	2/2/2	0/0/0	2/3/2	0/0/0	3/3/3	2/2/2	0/0/0	3/2/2	0/0/0	2/2/2	0/0/0	0/0/0	0/0/0	0/0/0
Pradeep, 2022 [120]	3/3/3	2/2/2	0/0/0	0/0/0	2/2/2	1/1/1	0/0/0	2/2/2	1/1/1	3/3/3	2/3/2	2/2/2	2/2/2	2/2/2	0/0/0	0/0/0	0/0/0	NA/NA/NA	0/0/0
Prokopetc, 2015 [121]	3/3/3	3/3/3	2/2/2	2/2/2	0/2/2	0/0/0	0/0/0	0/0/0	2/2/2	2/2/2	0/0/0	2/2/2	2/2/2	0/2/2	0/3/2	0/2/0	0/2/0	0/0/0	0/0/0
Protserov, 2024 [122]	3/3/3	3/3/3	3/3/3	3/3/3	2/2/2	3/3/3	0/0/0	2/2/2	2/2/2	3/3/3	3/3/3	2/2/2	3/3/3	2/3/3	3/3/3	3/3/3	3/3/3	3/3/3	3/3/3
Rahbar, 2020 [123]	3/3/3	3/3/3	3/3/3	3/3/3	2/2/2	2/2/2	0/0/0	2/2/2	2/2/2	2/2/2	0/0/0	2/2/2	2/2/2	2/0/0	3/3/3	3/3/3	3/3/3	0/0/0	0/0/0
Rahbar, 2024 [124]	3/3/3	3/3/3	2/2/2	2/2/2	2/2/2	1/1/1	0/0/0	2/2/2	1/1/1	3/3/3	2/2/2	0/0/0	2/2/2	0/0/0	0/0/0	2/2/2	2/2/2	NA/NA/NA	2/2/2
Raja, 2024 [125]	3/3/3	2/2/2	0/2/0	0/3/0	0/2/0	1/1/1	0/0/0	2/2/2	1/1/1	3/3/3	2/3/2	2/2/2	2/2/2	0/2/2	0/0/0	0/0/0	0/0/0	NA/0/NA	0/3/0
Ryu, 2024 [126]	3/3/3	3/3/3	2/2/2	2/2/2	2/3/2	3/3/3	0/0/0	2/2/2	3/3/3	3/3/3	3/3/3	2/2/2	2/2/2	2/2/2	0/0/0	3/3/3	3/3/3	3/3/3	0/0/0
Sahu, 2017 [127]	3/3/3	2/2/2	2/2/2	2/2/2	2/2/2	1/1/1	1/1/1	2/2/2	1/1/1	3/3/3	2/2/2	2/2/2	3/3/3	3/3/3	3/3/3	0/0/0	2/2/2	NA/NA/NA	0/0/0
Samuel, 2021 [128]	3/3/3	2/2/2	0/0/0	0/0/0	2/2/2	1/1/1	0/0/0	2/2/2	0/0/0	3/3/3	2/2/2	2/2/2	2/2/2	0/0/0	2/2/2	0/0/0	0/0/0	NA/NA/NA	0/0/0
Sanchez-Matilla, 2022 [129]	3/3/3	2/2/2	0/0/0	0/0/0	2/2/2	1/1/1	0/0/0	2/2/2	1/1/1	3/3/3	3/3/3	2/2/2	2/3/2	2/2/2	0/0/0	0/0/0	0/0/0	NA/NA/NA	0/0/0
Satyanaik, 2024 [130]	3/3/3	3/3/3	2/2/2	0/0/0	2/2/2	1/1/1	0/0/0	2/2/2	1/1/1	3/3/3	2/2/2	2/2/2	2/2/2	2/2/2	0/0/0	0/0/0	0/0/0	NA/NA/NA	3/3/3
Satyanaik, 2024 [131]	2/2/2	2/2/2	2/2/2	2/2/2	2/2/2	3/3/3	0/0/0	2/2/2	2/2/2	3/3/3	2/2/2	2/2/2	2/2/2	2/2/2	0/0/0	2/2/2	0/0/0	3/3/3	3/3/3
Seenivasan, 2023 [132]	3/3/3	3/3/3	3/3/3	3/3/3	2/2/2	1/1/1	0/1/0	2/2/2	0/1/1	3/3/3	3/3/3	2/2/2	2/2/2	0/0/0	0/0/0	2/2/2	0/0/0	NA/NA/NA	3/3/3
Sengun, 2023 [133]	3/3/3	3/3/3	3/3/3	2/2/2	2/2/2	3/3/3	0/0/0	0/0/0	3/3/3	3/3/3	2/2/2	2/2/2	2/2/2	2/2/2	0/0/0	3/3/3	3/2/2	3/3/3	0/0/0
Sengun, 2024 [134]	3/3/3	3/3/3	2/2/2	3/3/3	2/2/2	3/3/3	2/2/2	0/0/0	3/3/3	3/3/3	0/0/0	3/3/3	2/2/2	0/0/0	0/0/0	3/3/3	2/2/2	3/3/3	0/0/0
Shen, 2023 [135]	3/3/3	2/2/2	2/2/2	2/2/2	2/2/2	1/1/1	0/0/0	2/2/2	1/1/1	3/3/3	3/3/3	2/2/2	2/2/2	2/2/2	0/0/0	0/0/0	0/0/0	0/0/0	3/3/3
Sheng, 2024 [136]	3/3/3	3/3/3	2/2/2	2/2/2	2/2/2	1/1/1	0/0/0	0/0/0	1/1/1	3/3/3	2/2/2	0/0/0	2/2/2	0/0/0	0/0/0	0/0/0	2/2/2	NA/NA/NA	3/3/3
Shi, 2020 [137]	3/3/3	3/3/3	2/2/2	2/2/2	2/2/2	1/1/1	0/0/0	2/2/2	0/0/0	3/3/3	3/3/3	2/2/2	2/2/2	2/3/2	0/0/0	2/3/2	2/2/2	0/0/0	0/0/0
Shimgekar, 2021 [138]	3/3/3	2/2/2	0/0/0	0/0/0	2/2/2	2/2/2	0/0/0	0/0/0	2/2/2	2/2/2	0/0/0	2/2/2	0/0/0	0/0/0	0/0/0	2/2/2	0/0/0	0/0/0	0/0/0
Silva, 2022 [139]	3/3/3	3/3/3	2/2/2	2/2/2	2/2/2	2/2/2	0/0/0	2/2/2	1/1/1	3/3/3	2/2/2	2/2/2	2/2/2	2/3/2	0/0/0	0/0/0	0/0/0	NA/NA/NA	0/0/0
Smithmaitrie, 2024 [140]	3/3/3	3/3/3	3/3/3	3/3/3	2/2/2	2/2/2	2/2/2	2/2/2	3/3/3	3/3/3	2/2/2	2/2/2	2/2/2	2/3/2	0/0/0	3/3/3	2/2/2	3/3/3	2/2/2
Sonsilphong, 2022 [141]	3/3/3	0/0/0	0/0/0	0/0/0	0/0/0	2/2/2	0/0/0	2/2/2	2/2/2	3/3/3	2/2/2	2/2/2	2/2/2	2/2/2	0/0/0	0/0/0	0/0/0	0/0/0	0/0/0
Streckert, 2023 [142]	3/3/3	3/3/3	0/0/0	0/0/0	2/2/2	1/1/1	0/0/0	2/2/2	1/1/1	3/3/3	2/2/2	2/2/2	2/2/2	0/0/0	0/0/0	0/0/0	0/0/0	NA/NA/NA	0/0/0
Strong, 2024 [143]	3/3/3	3/3/3	3/3/3	3/3/3	3/3/3	3/3/3	2/2/2	2/2/2	3/3/3	2/2/2	2/2/2	2/2/2	2/2/2	2/2/2	0/0/0	3/3/3	3/3/3	3/3/3	0/0/0
Sun, 2022 [144]	3/3/3	3/3/3	2/2/2	2/2/2	2/2/2	0/0/0	0/0/0	2/2/2	0/0/0	3/3/3	3/3/3	2/2/2	2/2/2	2/2/2	3/3/3	2/2/2	0/0/0	NA/NA/NA	2/2/2
Takeuchi, 2023 [145]	3/3/3	3/3/3	3/3/3	3/3/3	2/2/2	3/3/3	2/2/2	2/2/2	2/2/2	3/3/3	2/2/2	2/2/2	2/2/2	0/0/0	0/0/0	3/3/3	3/3/3	3/3/3	2/2/2
Tao, 2023 [146]	3/3/3	3/3/3	2/2/2	2/2/2	2/2/2	1/1/1	0/0/0	2/2/2	1/1/1	3/3/3	3/3/3	2/2/2	3/3/3	3/3/3	0/0/0	2/2/2	0/0/0	NA/NA/NA	0/0/0
Tokuyasu, 2021 [147]	3/3/3	3/3/3	3/3/3	3/3/3	2/2/2	2/2/2	3/3/3	2/3/2	3/3/3	3/3/3	2/2/2	2/2/2	2/2/2	2/3/2	0/0/0	3/3/3	2/2/2	2/2/2	0/0/0
Urrea, 2024 [148]	3/3/3	3/3/3	2/2/2	2/2/2	2/2/2	1/1/1	0/0/0	3/3/3	1/1/1	3/3/3	3/3/3	2/2/2	2/2/2	2/2/2	2/2/2	2/2/2	2/2/2	NA/NA/NA	2/2/2
Valderrama, 2022 [149]	3/3/3	3/3/3	2/2/2	2/2/2	2/2/2	2/2/2	2/2/2	2/2/2	2/2/2	3/3/3	2/2/2	2/2/2	2/2/2	0/0/0	0/0/0	0/0/0	0/0/0	3/3/3	3/3/3
Vardazaryan, 2018 [150]	3/3/3	2/2/2	0/0/0	0/0/0	0/0/0	1/1/1	0/0/0	2/2/2	0/0/0	3/3/3	2/2/2	2/2/2	2/2/2	0/0/0	2/2/2	0/0/0	0/0/0	0/0/0	0/0/0
Wagner, 2023 [151]	3/3/3	3/3/3	3/3/3	3/3/3	2/3/2	3/3/3	2/2/2	2/2/2	3/3/3	3/3/3	1/1/1	1/3/3	3/3/3	3/3/3	3/3/3	3/3/3	3/3/3	NA/NA/NA	3/3/3
Wang, 2023 [152]	3/3/3	3/3/3	2/2/2	2/2/2	3/3/3	1/1/1	1/1/1	2/2/2	1/1/1	3/3/3	2/2/2	2/2/2	2/2/2	2/2/2	0/0/0	0/0/0	0/0/0	NA/NA/NA	0/0/0
Wang, 2017 [153]	3/3/3	2/2/2	2/2/2	0/3/2	2/2/2	1/1/1	0/0/0	2/2/2	1/1/1	3/3/3	2/2/2	2/2/2	2/2/2	0/0/0	0/0/0	0/0/0	0/0/0	NA/NA/NA	0/0/0
Wang, 2024 [154]	3/3/3	2/2/2	0/0/0	0/0/0	2/2/2	1/1/1	0/0/0	2/2/2	1/1/1	3/3/3	3/3/3	2/2/2	3/3/3	3/3/3	0/0/0	0/0/0	0/0/0	NA/NA/NA	0/0/0
Wang, 2023 [155]	3/3/3	3/3/3	3/3/3	3/3/3	3/3/3	2/2/2	0/0/0	2/3/2	3/3/3	3/3/3	3/3/3	2/3/2	2/2/2	2/2/2	2/2/2	2/2/2	3/3/3	3/3/3	2/2/2
Wang, 2025 [156]	3/3/3	3/3/3	2/2/2	2/2/2	3/3/3	1/1/1	0/0/0	2/2/2	1/1/1	3/3/3	2/2/2	1/1/1	2/2/2	3/3/3	0/0/0	0/0/0	0/0/0	NA/NA/NA	0/0/0
Wang, 2021 [157]	3/3/3	2/2/2	0/0/0	0/0/0	3/2/2	1/1/1	0/0/0	2/2/2	1/1/1	3/3/3	2/2/2	2/2/2	2/2/2	2/2/2	3/3/3	0/0/0	0/0/0	NA/NA/NA	0/0/0
Wang, 2020 [158]	3/3/3	2/2/2	0/2/2	0/0/0	2/2/2	2/3/3	0/0/0	2/2/2	2/2/2	3/3/3	2/2/2	2/2/2	2/2/2	2/2/2	0/0/0	0/0/0	0/0/0	0/0/0	0/0/0
Wang, 2024 [159]	3/3/3	2/2/2	2/2/2	0/0/0	2/2/2	1/1/1	0/0/0	2/2/2	1/1/1	3/3/3	2/2/2	2/2/2	2/2/2	2/2/2	2/2/2	0/0/0	0/0/0	NA/NA/NA	0/0/0
Wang, 2023 [160]	3/3/3	2/2/2	2/2/2	0/0/0	2/2/2	1/1/1	0/0/0	2/2/2	0/0/0	3/3/3	3/3/3	2/2/2	2/2/2	2/2/2	0/0/0	0/0/0	0/0/0	NA/NA/NA	0/0/0
Ward, 2022 [161]	3/3/3	3/3/3	3/3/3	2/3/2	2/2/2	2/2/2	0/0/0	2/3/3	3/3/3	3/3/3	0/0/0	2/2/2	2/2/2	2/3/2	0/0/0	3/3/3	3/3/3	3/3/3	0/0/0
Wei, 2024 [162]	3/3/3	2/2/2	0/0/0	0/0/0	2/2/2	1/1/1	0/0/0	2/2/2	1/1/1	3/3/3	2/2/2	2/2/2	2/2/2	2/2/2	0/0/0	2/2/2	0/0/0	NA/NA/NA	3/3/3
Wei, 2023 [163]	3/3/3	3/3/3	2/2/2	0/0/0	2/2/2	1/1/1	0/0/0	2/2/2	1/1/1	3/3/3	3/3/3	3/3/3	3/3/3	2/2/2	0/0/0	0/0/0	0/0/0	NA/NA/NA	0/0/0
Wu, 2024 [164]	3/3/3	3/3/3	2/2/2	2/2/2	2/2/2	1/1/1	0/0/0	2/2/2	1/1/1	3/3/3	3/3/3	2/2/2	2/2/2	2/2/2	0/0/0	2/2/2	0/0/0	0/0/0	3/3/3
Xi, 2022 [165]	3/3/3	2/2/2	0/0/0	0/0/0	2/2/2	1/1/1	0/0/0	2/2/2	1/1/1	3/3/3	0/0/0	2/2/2	2/3/2	2/2/2	0/0/0	0/0/0	2/2/2	NA/NA/NA	0/0/0
Xu, 2024 [166]	3/3/3	3/3/3	0/0/0	0/0/0	3/3/3	1/1/1	0/0/0	3/3/3	1/1/1	3/3/3	3/3/3	2/2/2	2/2/2	2/2/2	0/0/0	0/0/0	0/0/0	NA/NA/NA	3/3/3
Xue, 2022 [167]	3/3/3	2/2/2	2/2/2	0/0/0	2/2/2	1/1/1	0/0/0	3/3/3	1/1/1	3/3/3	3/3/3	3/3/3	2/2/2	2/2/2	0/0/0	0/0/0	0/0/0	NA/NA/NA	0/0/0
Yamazaki, 2020 [168]	3/3/3	2/2/2	0/0/0	0/0/0	2/2/2	3/3/3	0/0/0	2/2/2	2/2/2	3/3/3	2/2/2	2/3/2	2/2/2	2/2/2	0/0/0	0/0/0	3/3/3	3/3/3	0/0/0
Yamlahi, 2023 [169]	3/3/3	3/3/3	0/0/0	0/0/0	2/2/2	1/1/1	0/0/0	2/2/2	1/1/1	3/3/3	3/3/3	1/1/1	2/2/2	0/0/0	0/0/0	0/0/0	0/0/0	NA/NA/NA	0/0/0
Yang, 2024 [170]	3/3/3	3/3/3	0/0/0	0/0/0	2/2/2	1/1/1	1/1/1	0/0/0	1/1/1	2/2/2	0/0/0	2/2/2	2/2/2	0/0/0	0/0/0	2/2/2	2/2/2	NA/NA/NA	0/0/0
Yang, 2023 [171]	3/3/3	3/3/3	2/2/2	2/2/2	2/2/2	2/2/2	0/2/0	2/2/2	2/2/2	3/3/3	3/3/3	2/2/2	2/2/2	2/2/2	0/0/0	0/0/0	0/0/0	2/2/2	0/0/0
Yang, 2021 [172]	3/3/3	3/3/3	2/2/2	0/0/0	2/2/2	1/1/1	0/0/0	2/2/2	1/1/1	3/3/3	3/3/3	2/2/2	2/2/2	0/0/0	3/3/3	0/0/0	2/2/2	NA/NA/NA	3/3/3
Yin, 2024 [173]	3/3/3	3/3/3	2/2/2	2/2/2	2/2/2	1/1/1	0/0/0	2/2/2	1/1/1	3/3/3	2/2/2	2/2/2	2/2/2	2/2/2	0/0/0	2/2/2	2/2/2	NA/NA/NA	0/0/0
Yuan, 2024 [174]	3/3/3	3/3/3	2/2/2	2/2/2	2/2/2	1/1/1	2/2/2	1/1/1	1/1/1	3/3/3	2/2/2	2/2/2	2/2/2	2/2/2	0/0/0	0/0/0	0/0/0	NA/NA/NA	3/3/3
Zhang, 2024 [175]	3/3/3	3/3/3	2/2/2	2/2/2	2/2/2	2/2/2	0/0/0	2/2/2	2/2/2	3/3/3	3/3/3	2/2/2	2/2/2	2/2/2	0/0/0	2/2/2	2/2/2	3/3/3	0/0/0
Zhang, 2023 [176]	3/3/3	3/3/3	2/2/2	2/2/2	2/2/2	2/2/2	0/0/0	2/2/2	2/2/2	3/3/3	2/2/2	2/2/2	3/3/3	2/2/2	2/2/2	3/3/3	3/3/3	3/3/3	3/3/3
Zhang, 2023 [177]	3/3/3	2/2/2	0/0/0	2/3/3	2/2/2	1/1/1	0/0/0	2/2/2	1/1/1	3/3/3	2/2/2	2/2/2	2/2/2	2/2/2	2/2/2	2/2/2	2/2/2	NA/NA/NA	0/0/0
Zhang, 2020 [178]	3/3/3	2/2/2	2/2/2	2/3/2	2/2/2	1/3/1	0/0/0	2/2/2	2/2/2	3/3/3	2/2/2	2/2/2	2/2/2	0/0/0	0/0/0	2/2/2	0/0/0	NA/NA/NA	0/0/0
Zhang, 2023 [179]	3/3/3	2/2/2	0/0/0	0/0/0	2/2/2	2/3/3	0/0/0	2/2/2	2/2/2	3/3/3	2/2/2	2/2/2	2/2/2	2/2/2	0/0/0	0/0/0	0/0/0	0/0/0	0/0/0
Zhang, 2020 [180]	3/3/3	3/3/3	3/3/3	3/3/3	2/2/2	1/1/1	0/0/0	2/2/2	1/0/1	3/3/3	2/2/2	2/2/2	2/2/2	2/2/2	0/0/0	3/3/3	0/0/0	NA/NA/NA	0/0/0
Zhao, 2021 [181]	3/3/3	3/3/3	2/2/2	2/2/2	3/3/3	2/2/2	0/0/0	2/2/2	1/1/1	3/3/3	2/2/2	2/2/2	2/2/2	0/0/0	0/0/0	2/2/2	2/2/2	0/2/0	0/2/0
Zhao, 2023 [182]	3/3/3	3/3/3	0/3/0	0/2/0	2/2/2	1/1/1	0/0/0	2/2/2	2/2/2	3/3/3	3/3/3	2/2/2	2/2/2	2/2/2	0/0/0	0/0/0	0/0/0	NA/NA/NA	0/0/0
Zhao, 2021 [183]	3/3/3	2/2/2	0/0/0	0/0/0	2/2/2	1/1/1	0/0/0	2/2/2	1/1/1	3/3/3	3/3/3	2/2/2	2/2/2	2/2/2	0/0/0	0/0/0	0/0/0	NA/NA/NA	0/0/0
Zhao, 2019 [184]	3/3/3	3/3/3	2/2/2	2/2/2	2/2/2	2/1/2	0/0/0	2/2/2	0/0/0	3/3/3	3/3/3	2/2/2	3/3/3	2/2/2	2/2/2	0/0/0	0/0/0	0/0/0	0/0/0
Zheng, 2023 [185]	3/3/3	3/3/3	2/2/2	2/3/3	2/2/2	2/2/2	2/2/2	3/3/3	3/3/3	3/3/3	2/2/2	2/2/2	2/2/2	2/2/2	0/0/0	3/3/3	3/3/3	3/3/3	2/3/2
Zhou, 2024 [186]	3/3/3	2/2/2	0/2/2	0/2/2	2/2/2	1/1/1	0/0/0	0/0/0	2/2/2	3/3/3	2/2/2	2/2/2	2/2/2	0/0/0	0/0/0	0/0/0	0/0/0	NA/NA/NA	0/0/0
Zhou, 2022 [187]	3/3/3	3/3/3	2/2/2	2/2/2	2/2/2	1/1/1	0/0/0	2/2/2	2/2/2	3/3/3	2/2/2	2/2/2	2/2/2	2/2/2	0/0/0	0/0/0	0/0/0	NA/NA/NA	0/0/0
Zygomalas, 2024 [188]	3/3/3	2/2/2	2/2/2	2/2/2	0/0/0	2/2/2	0/0/0	0/0/0	2/2/2	3/3/3	0/0/0	2/2/2	2/2/2	0/0/0	2/2/2	2/2/2	2/2/2	2/2/2	0/0/0

Supplementary Table 8: Reporting Quality Across JCR-Based Journal Categories

All journals were categorized into three groups based on Clarivate Journal Citation Reports (JCR) categories: medical journals (purely medical content), interdisciplinary journals (medical and technical), and technical-only journals (primarily engineering, biomedical engineering, robotics, or computer science). Each reporting quality category was compared between these groups using the Kruskal-Wallis test, followed by Dunn's post hoc tests with Bonferroni correction for pairwise comparisons. Table 8 shows the medians for each reporting quality criterion across the JCR-based journal groups, using the following coding: Reported = 3, Partially Reported = 2, Reference to another publication = 1, Not Reported = 0, and displays the p-values from the Kruskal–Wallis tests and Dunn's post hoc comparisons.

Reporting Quality Criterium	JCR Group	Median	P-value (Kruskal-Wallis test)	Group 1	Group 2	P-value (after Bonferroni correction) (Dunn test)
Study Design	Medical Journals	3	0.499	Medical Journals	Multidisciplinary Journals	1
	Multidisciplinary Journals	3		Medical Journals	Technical Journals	0.765325324994855
	Technical Journals	3		Multidisciplinary Journals	Technical Journals	1
Prediction Problem	Medical Journals	3	0.00015	Medical Journals	Multidisciplinary Journals	0.073132530180647
	Multidisciplinary Journals	3		Medical Journals	Technical Journals	9.9850717723128E-05
	Technical Journals	2		Multidisciplinary Journals	Technical Journals	0.0537047330531948
Clinical Problem	Medical Journals	3	7.92E-09	Medical Journals	Multidisciplinary Journals	2.73016394291022E-07
	Multidisciplinary Journals	2		Medical Journals	Technical Journals	2.88425703895975E-08
	Technical Journals	2		Multidisciplinary Journals	Technical Journals	1
Clinical Goal	Medical Journals	2	2.55E-07	Medical Journals	Multidisciplinary Journals	2.9449504203937E-05
	Multidisciplinary Journals	2		Medical Journals	Technical Journals	2.21253639239217E-07
	Technical Journals	0		Multidisciplinary Journals	Technical Journals	0.60143721403075
Existing AI	Medical Journals	2	0.108	Medical Journals	Multidisciplinary Journals	1
	Multidisciplinary Journals	2		Medical Journals	Technical Journals	0.824635275494482
	Technical Journals	2		Multidisciplinary Journals	Technical Journals	0.107407641222633
Data Source	Medical Journals	2	1.31E-11	Medical Journals	Multidisciplinary Journals	9.96126730348103E-08
	Multidisciplinary Journals	1		Medical Journals	Technical Journals	9.86787604187975E-12
	Technical Journals	1		Multidisciplinary Journals	Technical Journals	0.175164604457521
Data Selection	Medical Journals	2	1.67E-08	Medical Journals	Multidisciplinary Journals	6.09188781125351E-05
	Multidisciplinary Journals	0		Medical Journals	Technical Journals	7.2810454428434E-09
	Technical Journals	0		Multidisciplinary Journals	Technical Journals	0.0873072048233087
Data Preprocessing	Medical Journals	2	0.611	Medical Journals	Multidisciplinary Journals	1
	Multidisciplinary Journals	2		Medical Journals	Technical Journals	1
	Technical Journals	2		Multidisciplinary Journals	Technical Journals	1
Labeling	Medical Journals	3	1.44E-08	Medical Journals	Multidisciplinary Journals	6.67666677096481E-08
	Multidisciplinary Journals	1		Medical Journals	Technical Journals	2.9947559703384E-07
	Technical Journals	1		Multidisciplinary Journals	Technical Journals	1
Model Type	Medical Journals	3	0.0553	Medical Journals	Multidisciplinary Journals	0.0556118184830427
	Multidisciplinary Journals	3		Medical Journals	Technical Journals	0.165249650645181
	Technical Journals	3		Multidisciplinary Journals	Technical Journals	1
Model Development	Medical Journals	2	0.00225	Medical Journals	Multidisciplinary Journals	0.00514704999470532
	Multidisciplinary Journals	2		Medical Journals	Technical Journals	1
	Technical Journals	2		Multidisciplinary Journals	Technical Journals	0.0242649184928813
Model Validation	Medical Journals	2	0.4	Medical Journals	Multidisciplinary Journals	1
	Multidisciplinary Journals	2		Medical Journals	Technical Journals	0.528331531720964
	Technical Journals	2		Multidisciplinary Journals	Technical Journals	1
Model Performance	Medical Journals	2	0.00305	Medical Journals	Multidisciplinary Journals	0.0652087331103511
	Multidisciplinary Journals	2		Medical Journals	Technical Journals	1
	Technical Journals	2		Multidisciplinary Journals	Technical Journals	0.00391209438840239
Statistical Methods	Medical Journals	2	0.0867	Medical Journals	Multidisciplinary Journals	1
	Multidisciplinary Journals	2		Medical Journals	Technical Journals	0.916285120635758
	Technical Journals	2		Multidisciplinary Journals	Technical Journals	0.0812204904855827
Performance Errors	Medical Journals	0	0.0546	Medical Journals	Multidisciplinary Journals	0.218077119662363
	Multidisciplinary Journals	0		Medical Journals	Technical Journals	1
	Technical Journals	0		Multidisciplinary Journals	Technical Journals	0.0860241498328219
Clinical Implications	Medical Journals	3	3.71E-11	Medical Journals	Multidisciplinary Journals	1.61284089238289E-08
	Multidisciplinary Journals	0		Medical Journals	Technical Journals	8.81760085061061E-11
	Technical Journals	0		Multidisciplinary Journals	Technical Journals	0.7656533258143
Limitations	Medical Journals	3	7.93E-13	Medical Journals	Multidisciplinary Journals	6.74958540629431E-11
	Multidisciplinary Journals	0		Medical Journals	Technical Journals	1.69258447462909E-11
	Technical Journals	0		Multidisciplinary Journals	Technical Journals	1
Ethical Statement	Medical Journals	3	1.72E-05	Medical Journals	Multidisciplinary Journals	0.0648486908940682
	Multidisciplinary Journals	3		Medical Journals	Technical Journals	1.33805329647371E-05
	Technical Journals	0		Multidisciplinary Journals	Technical Journals	0.0132301601652264
Code Publication	Medical Journals	0	0.0138	Medical Journals	Multidisciplinary Journals	0.983601033186889
	Multidisciplinary Journals	0		Medical Journals	Technical Journals	0.449472967005708
	Technical Journals	0		Multidisciplinary Journals	Technical Journals	0.0105135703624522

Supplementary Figure 2: Reporting Quality Across JCR-Based Journal Categories

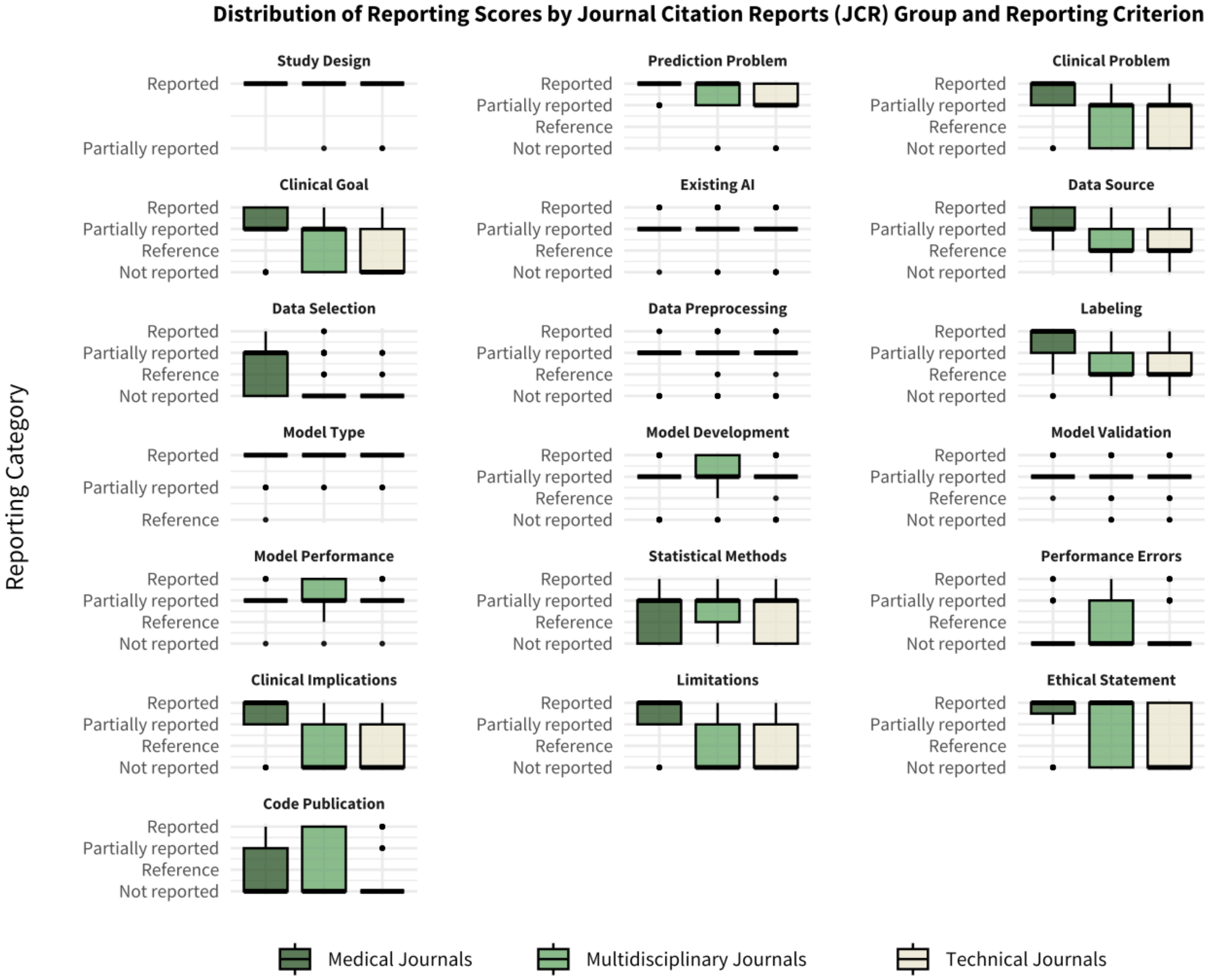

Boxplots show the distribution of reporting quality scores by category and Clarivate Journal Citation Reports (JCR) category group, based on the journal type: medical journals (purely medical content), interdisciplinary journals (medical and technical), and technical-only journals (primarily engineering, biomedical engineering, robotics, or computer science). The central line represents the median, the box shows the interquartile range (IQR: 25th–75th percentile), and the whiskers extend to the most extreme data points within 1.5×IQR from the box. Individual points represent outliers beyond this range.

References of all included studies

1. Acharya, D., Guda, R. K. S. & Raovenkatajammalamadaka, K. Enhanced EfficientNet Network for Classifying Laparoscopy Videos using Transfer Learning Technique. in *2022 International Joint Conference on Neural Networks (IJCNN)* 1–9 (IEEE, Padua, Italy, 2022). doi:10.1109/IJCNN55064.2022.9891989.

2. Al Hajj, H., Lamard, M., Conze, P.-H., Cochener, B. & Quellec, G. Monitoring tool usage in surgery videos using boosted convolutional and recurrent neural networks. *Medical Image Analysis* **47**, 203–218 (2018).

3. Alapatt, D. *et al.* Temporally Constrained Neural Networks (TCNN): A framework for semi-supervised video semantic segmentation. Preprint at https://doi.org/10.48550/ARXIV.2112.13815 (2021).

4. Ali, M., Ochoa-Ruiz, G. & Ali, S. A semi-supervised Teacher-Student framework for surgical tool detection and localization. (2022) doi:10.48550/ARXIV.2208.09926.

5. Alkhamaiseh, K. N., Grantner, J. L., Shebrain, S. & Abdel-Qader, I. Towards reliable hepatocytic anatomy segmentation in laparoscopic cholecystectomy using U-Net with Auto-Encoder. *Surg Endosc* **37**, 7358–7369 (2023).

6. Abdulbaki Alshirbaji, T., Jalal, N. A., Docherty, P. D., Neumuth, T. & Möller, K. A deep learning spatial-temporal framework for detecting surgical tools in laparoscopic videos. *Biomedical Signal Processing and Control* **68**, 102801 (2021).

7. Alshirbaji, T. A., Jalal, N. A., Docherty, P. D., Neumuth, T. & Moeller, K. Assessing Generalisation Capabilities of CNN Models for Surgical Tool Classification. *Current Directions in Biomedical Engineering* **7**, 476–479 (2021).

8. Abdulbaki Alshirbaji, T., Jalal, N. A. & Möller, K. Surgical Tool Classification in Laparoscopic Videos Using Convolutional Neural Network. *Current Directions in Biomedical Engineering* **4**, 407–410 (2018).

9. Angeles Ceron, J. C., Chang, L., Ruiz, G. O. & Ali, S. Assessing YOLACT++ for real time and robust instance segmentation of medical instruments in endoscopic procedures. in *2021 43rd Annual International Conference of the IEEE Engineering in Medicine & Biology Society (EMBC)* 1824–1827 (IEEE, Mexico, 2021). doi:10.1109/EMBC46164.2021.9629914.

10. Cerón, J. C. Á., Ruiz, G. O., Chang, L. & Ali, S. Real-time instance segmentation of surgical instruments using attention and multi-scale feature fusion. *Medical Image Analysis* **81**, 102569 (2022).

11. Aoyama, Y. *et al.* Artificial intelligence for surgical safety during laparoscopic gastrectomy for gastric cancer: Indication of anatomical landmarks related to postoperative pancreatic fistula using deep learning. *Surg Endosc* **38**, 5601–5612 (2024).

12. Arabian, H., Dalla, F. A., Jalal, N. A., Alshirbaji, T. A. & Moeller, K. Attention Networks for Improving Surgical Tool Classification in Laparoscopic Videos. *Current Directions in Biomedical Engineering* **8**, 676–679 (2022).

13. Arabian, H., Abdulbaki Alshirbaji, T., Jalal, N. A., Krueger-Ziolek, S. & Moeller, K. P-CSEM: An Attention Module for Improved Laparoscopic Surgical Tool Detection. *Sensors* **23**, 7257 (2023).

14. Aspart, F. *et al.* ClipAssistNet: bringing real-time safety feedback to operating rooms. *Int J CARS* **17**, 5–13 (2022).

15. Attia, M., Hossny, M., Nahavandi, S. & Asadi, H. Surgical tool segmentation using a hybrid deep CNN-RNN auto encoder-decoder. in *2017 IEEE International Conference on Systems, Man, and Cybernetics (SMC)* 3373–3378 (IEEE, Banff, AB, 2017). doi:10.1109/SMC.2017.8123151.

16. Ayobi, N. *et al.* Pixel-Wise Recognition for Holistic Surgical Scene Understanding. Preprint at https://doi.org/10.48550/ARXIV.2401.11174 (2024).

17. Bai, L., Islam, M. & Ren, H. Revisiting Distillation for Continual Learning on Visual Question Localized-Answering in Robotic Surgery. Preprint at https://doi.org/10.48550/ARXIV.2307.12045 (2023).

18. Bakker, A. F. H. A. *et al.* Estimating Surgical Urethral Length on Intraoperative Robot-Assisted Prostatectomy Images Using Artificial Intelligence Anatomy Recognition. *Journal of Endourology* **38**, 690–696 (2024).

19. Bamba, Y. *et al.* Object and anatomical feature recognition in surgical video images based on a convolutional neural network. *Int J CARS* **16**, 2045–2054 (2021).

20. Bamba, Y. *et al.* Automated recognition of objects and types of forceps in surgical images using deep learning. *Sci Rep* **11**, 22571 (2021).

21. Ban, Y. *et al.* Concept Graph Neural Networks for Surgical Video Understanding. Preprint at https://doi.org/10.48550/ARXIV.2202.13402 (2022).

22. Batić, D., Holm, F., Özsoy, E., Czempiel, T. & Navab, N. EndoViT: pretraining vision transformers on a large collection of endoscopic images. *Int J CARS* **19**, 1085–1091 (2024).

23. Batić, D., Holm, F., Özsoy, E., Czempiel, T. & Navab, N. Whether and When does Endoscopy Domain

Pretraining Make Sense? Preprint at https://doi.org/10.48550/ARXIV.2303.17636 (2023).

24. Boonkong, A., Hormdee, D., Sonsilphong, S. & Khampitak, K. Surgical Instrument Detection for Laparoscopic Surgery using Deep Learning. in *2022 19th International Conference on Electrical Engineering/Electronics, Computer, Telecommunications and Information Technology (ECTI-CON)* 1–4 (IEEE, Prachuap Khiri Khan, Thailand, 2022). doi:10.1109/ECTI-CON54298.2022.9795561.

25. Brandenburg, J. M. *et al.* Active learning for extracting surgomic features in robot-assisted minimally invasive esophagectomy: a prospective annotation study. *Surg Endosc* **37**, 8577–8593 (2023).

26. Casella, A. *et al.* NephCNN: A deep-learning framework for vessel segmentation in nephrectomy laparoscopic videos. in *2020 25th International Conference on Pattern Recognition (ICPR)* 6144–6149 (IEEE, Milan, Italy, 2021). doi:10.1109/ICPR48806.2021.9412810.

27. Chen, J., Li, M., Han, H., Zhao, Z. & Chen, X. SurgNet: Self-Supervised Pretraining With Semantic Consistency for Vessel and Instrument Segmentation in Surgical Images. *IEEE Trans. Med. Imaging* **43**, 1513–1525 (2024).

28. Chen, C.-J., Huang, W. S.-W. & Song, K.-T. Image tracking of laparoscopic instrument using spiking neural networks. in *2013 13th International Conference on Control, Automation and Systems (ICCAS 2013)* 951–955 (IEEE, Gwangju, Korea (South), 2013). doi:10.1109/ICCAS.2013.6704052.

29. Chen, Z., Zhao, Z. & Cheng, X. Surgical instruments tracking based on deep learning with lines detection and spatio-temporal context. in *2017 Chinese Automation Congress (CAC)* 2711–2714 (IEEE, Jinan, 2017). doi:10.1109/CAC.2017.8243236.

30. Choi, B., Jo, K., Choi, S. & Choi, J. Surgical-tools detection based on Convolutional Neural Network in laparoscopic robot-assisted surgery. in *2017 39th Annual International Conference of the IEEE Engineering in Medicine and Biology Society (EMBC)* 1756–1759 (IEEE, Seogwipo, 2017). doi:10.1109/EMBC.2017.8037183.

31. Ciaparrone, G. *et al.* A comparative analysis of multi-backbone Mask R-CNN for surgical tools detection. in *2020 International Joint Conference on Neural Networks (IJCNN)* 1–8 (IEEE, Glasgow, United Kingdom, 2020). doi:10.1109/IJCNN48605.2020.9206854.

32. Colleoni, E., Sanchez Matilla, R., Luengo, I. & Stoyanov, D. Guided image generation for improved surgical image segmentation. *Medical Image Analysis* **97**, 103263 (2024).

33. Colleoni, E., Psychogyios, D., Van Amsterdam, B., Vasconcelos, F. & Stoyanov, D. SSIS-Seg: Simulation-Supervised Image Synthesis for Surgical Instrument Segmentation. *IEEE Trans. Med. Imaging* **41**, 3074–3086 (2022).

34. Daneshgar Rahbar, M. & Mousavi Mojab, S. Z. Enhanced U-Net with GridMask (EUGNet): A Novel Approach for Robotic Surgical Tool Segmentation. *J. Imaging* **9**, 282 (2023).

35. Davila, A., Colan, J. & Hasegawa, Y. Gradient-Based Fine-Tuning Strategy for Improved Transfer Learning on Surgical Images. in *2023 International Symposium on Micro-NanoMehatronics and Human Science (MHS)* 1–5 (IEEE, Nagoya, Japan, 2023). doi:10.1109/MHS59931.2023.10510130.

36. De Backer, P. *et al.* Improving Augmented Reality Through Deep Learning: Real-time Instrument Delineation in Robotic Renal Surgery. *European Urology* **84**, 86–91 (2023).

37. Den Boer, R. B. *et al.* Deep learning-based recognition of key anatomical structures during robot-assisted minimally invasive esophagectomy. *Surg Endosc* **37**, 5164–5175 (2023).

38. Derathé, A. *et al.* Predicting the quality of surgical exposure using spatial and procedural features from laparoscopic videos. *Int J CARS* **15**, 59–67 (2020).

39. Du, X. *et al.* Patch-based adaptive weighting with segmentation and scale (PAWSS) for visual tracking in surgical video. *Medical Image Analysis* **57**, 120–135 (2019).

40. Endo, Y. *et al.* Impact of AI system on recognition for anatomical landmarks related to reducing bile duct injury during laparoscopic cholecystectomy. *Surg Endosc* **37**, 5752–5759 (2023).

41. Fernández-Rodríguez, M. *et al.* Exploring Optical Flow Inclusion into nnU-Net Framework for Surgical Instrument Segmentation. (2024) doi:10.48550/ARXIV.2403.10216.

42. Fuentes-Hurtado, F. *et al.* EasyLabels: weak labels for scene segmentation in laparoscopic videos. *Int J CARS* **14**, 1247–1257 (2019).

43. Fujinaga, A. *et al.* Development of a cross-artificial intelligence system for identifying intraoperative anatomical landmarks and surgical phases during laparoscopic cholecystectomy. *Surg Endosc* **37**, 6118–6128 (2023).

44. Ghamsarian, N., Wolf, S., Zinkernagel, M., Schoeffmann, K. & Sznitman, R. DeepPyramid+: medical image segmentation using Pyramid View Fusion and Deformable Pyramid Reception. *Int J CARS* **19**, 851–859 (2024).

45. Gitau, S. N., Abd El–Malek, A. H., Sayed, M. S. & Abo–Zahhad, M. Automatic Surgical Items Tracking in Laparoscopic Surgery Utilizing Deep Learning for Retained Items Detection and Classification. in *2024 IEEE 30th International Conference on*

Telecommunications (ICT) 01–06 (IEEE, Amman, Jordan, 2024). doi:10.1109/ICT62760.2024.10606122.

46. Grammatikopoulou, M. *et al.* A spatio-temporal network for video semantic segmentation in surgical videos. *Int J CARS* **19**, 375–382 (2023).

47. Guédon, A. C. P. *et al.* Deep learning for surgical phase recognition using endoscopic videos. *Surg Endosc* **35**, 6150–6157 (2021).

48. Hasan, S. M. K., Simon, R. A. & Linte, C. A. Segmentation and removal of surgical instruments for background scene visualization from endoscopic/laparoscopic video. in *Medical Imaging 2021: Image-Guided Procedures, Robotic Interventions, and Modeling* (eds. Linte, C. A. & Siewerdsen, J. H.) 7 (SPIE, Online Only, United States, 2021). doi:10.1117/12.2580668.

49. Huang, B. *et al.* Simultaneous Depth Estimation and Surgical Tool Segmentation in Laparoscopic Images. *IEEE Trans. Med. Robot. Bionics* **4**, 335–338 (2022).

50. Jalal, N. A. *et al.* Analysing attention convolutional neural network for surgical tool localisation: a feasibility study. *Current Directions in Biomedical Engineering* **8**, 548–551 (2022).

51. Jalal, N. A. *et al.* Laparoscopic Video Analysis Using Temporal, Attention, and Multi-Feature Fusion Based-Approaches. *Sensors* **23**, 1958 (2023).

52. Jamal, M. A. & Mohareri, O. Rethinking RGB-D Fusion for Semantic Segmentation in Surgical Datasets. Preprint at https://doi.org/10.48550/ARXIV.2407.19714 (2024).

53. Jang, Y. E., Jang, I. & Kim, K.-J. Tracker Learning Surgical Images By Self-Supervised Learning: An Enhanced Unsupervised Deep Tracking Approach. in *2023 International Conference on Platform Technology and Service (PlatCon)* 114–117 (IEEE, Busan, Korea, Republic of, 2023). doi:10.1109/PlatCon60102.2023.10255175.

54. Jaspers, T. J. M. *et al.* Exploring the Effect of Dataset Diversity in Self-supervised Learning for Surgical Computer Vision. in *Data Engineering in Medical Imaging* (eds. Bhattarai, B. et al.) vol. 15265 43–53 (Springer Nature Switzerland, Cham, 2025).

55. Jearanai, S., Wangkulangkul, P., Sae-Lim, W. & Cheewatanakornkul, S. Development of a deep learning model for safe direct optical trocar insertion in minimally invasive surgery: an innovative method to prevent trocar injuries. *Surg Endosc* **37**, 7295–7304 (2023).

56. Jha, D. *et al.* Exploring Deep Learning Methods for Real-Time Surgical Instrument Segmentation in Laparoscopy. (2021) doi:10.48550/ARXIV.2107.02319.

57. Jin, Y. *et al.* Multi-task recurrent convolutional network with correlation loss for surgical video analysis. *Medical Image Analysis* **59**, 101572 (2020).

58. Jin, A. *et al.* Tool Detection and Operative Skill Assessment in Surgical Videos Using Region-Based Convolutional Neural Networks. Preprint at https://doi.org/10.48550/ARXIV.1802.08774 (2018).

59. Hasan, Md. K., Calvet, L., Rabbani, N. & Bartoli, A. Detection, segmentation, and 3D pose estimation of surgical tools using convolutional neural networks and algebraic geometry. *Medical Image Analysis* **70**, 101994 (2021).

60. Kanakatte, A., Ramaswamy, A., Gubbi, J., Ghose, A. & Purushothaman, B. Surgical tool segmentation and localization using spatio-temporal deep network. in *2020 42nd Annual International Conference of the IEEE Engineering in Medicine & Biology Society (EMBC)* 1658–1661 (IEEE, Montreal, QC, Canada, 2020). doi:10.1109/EMBC44109.2020.9176676.

61. Kawamura, M. *et al.* Development of an artificial intelligence system for real-time intraoperative assessment of the Critical View of Safety in laparoscopic cholecystectomy. *Surg Endosc* **37**, 8755–8763 (2023).

62. Khalid, M. U. *et al.* Use of artificial intelligence for decision-support to avoid high-risk behaviors during laparoscopic cholecystectomy. *Surg Endosc* **37**, 9467–9475 (2023).

63. Khalid, S. & Rudzicz, F. SurGNN: Explainable visual scene understanding and assessment of surgical skill using graph neural networks. Preprint at https://doi.org/10.48550/ARXIV.2308.13073 (2023).

64. Kim, T. D., Dao, N.-N., Tran, D.-V., Tran, A. L.-Q. & Pham, D.-A. Surgical Tool Detection and Pose Estimation using YOLOv8-pose Model: A Study on Clipper Tool. in *2024 9th International Conference on Integrated Circuits, Design, and Verification (ICDV)* 225–229 (IEEE, Hanoi, Vietnam, 2024). doi:10.1109/ICDV61346.2024.10617290.

65. Kinoshita, K. *et al.* An artificial intelligence-based nerve recognition model is useful as surgical support technology and as an educational tool in laparoscopic and robot-assisted rectal cancer surgery. *Surg Endosc* **38**, 5394–5404 (2024).

66. Kitaguchi, D. *et al.* Artificial intelligence for the recognition of key anatomical structures in laparoscopic colorectal surgery. *British Journal of Surgery* **110**, 1355–1358 (2023).

67. Kitaguchi, D. *et al.* Development and Validation of a Model for Laparoscopic Colorectal Surgical Instrument Recognition Using Convolutional Neural Network–Based Instance Segmentation and Videos of Laparoscopic Procedures. *JAMA Netw Open* **5**, e2226265 (2022).

68. Kitaguchi, D. *et al.* Limited generalizability of single deep neural network for surgical instrument

segmentation in different surgical environments. *Sci Rep* **12**, 12575 (2022).

69. Kletz, S., Schoeffmann, K., Benois-Pineau, J. & Husslein, H. Identifying Surgical Instruments in Laparoscopy Using Deep Learning Instance Segmentation. in *2019 International Conference on Content-Based Multimedia Indexing (CBMI)* 1–6 (IEEE, Dublin, Ireland, 2019). doi:10.1109/CBMI.2019.8877379.

70. Kolbinger, F. R. *et al.* Anatomy segmentation in laparoscopic surgery: comparison of machine learning and human expertise – an experimental study. *International Journal of Surgery* (2023) doi:10.1097/JS9.0000000000000595.

71. Kolbinger, F. R. *et al.* Artificial Intelligence for context-aware surgical guidance in complex robot-assisted oncological procedures: An exploratory feasibility study. *European Journal of Surgical Oncology* **50**, 106996 (2024).

72. Kolbinger, F. R., He, J., Ma, J. & Zhu, F. Strategies to Improve Real-World Applicability of Laparoscopic Anatomy Segmentation Models. Preprint at https://doi.org/10.48550/ARXIV.2403.17192 (2024).

73. Kondo, S. LapFormer: surgical tool detection in laparoscopic surgical video using transformer architecture. *Computer Methods in Biomechanics and Biomedical Engineering: Imaging & Visualization* **9**, 302–307 (2021).

74. Sr Konduri, P. & Siva Nageswara Rao, G. Full resolution convolutional neural network based organ and surgical instrument classification on laparoscopic image data. *Biomedical Signal Processing and Control* **87**, 105533 (2024).

75. Kong, X. *et al.* Accurate instance segmentation of surgical instruments in robotic surgery: model refinement and cross-dataset evaluation. *Int J CARS* **16**, 1607–1614 (2021).

76. Kumazu, Y. *et al.* Automated segmentation by deep learning of loose connective tissue fibers to define safe dissection planes in robot-assisted gastrectomy. *Sci Rep* **11**, 21198 (2021).

77. Kumazu, Y. *et al.* AI-based visualization of loose connective tissue as a dissectable layer in gastrointestinal surgery. *Sci Rep* **15**, 152 (2025).

78. Labrunie, M. *et al.* Automatic preoperative 3d model registration in laparoscopic liver resection. *Int J CARS* **17**, 1429–1436 (2022).

79. Lam, K. *et al.* Deep Learning for Instrument Detection and Assessment of Operative Skill in Surgical Videos. *IEEE Trans. Med. Robot. Bionics* **4**, 1068–1071 (2022).

80. Laplante, S. *et al.* Validation of an artificial intelligence platform for the guidance of safe laparoscopic cholecystectomy. *Surg Endosc* **37**, 2260–2268 (2023).

81. Lavanchy, J. L. *et al.* Automation of surgical skill assessment using a three-stage machine learning algorithm. *Sci Rep* **11**, 5197 (2021).

82. Le, H.-B. *et al.* Robust Surgical Tool Detection in Laparoscopic Surgery using YOLOv8 Model. in *2023 International Conference on System Science and Engineering (ICSSE)* 537–542 (IEEE, Ho Chi Minh, Vietnam, 2023). doi:10.1109/ICSSE58758.2023.10227217.

83. Lee, M., Sanchez-Matilla, R., Stoyanov, D. & Luengo, I. DIPO: Differentiable Parallel Operation Blocks for Surgical Neural Architecture Search. *IEEE J. Biomed. Health Inform.* **28**, 5540–5550 (2024).

84. Leifman, G. *et al.* Real-time artificial intelligence validation of critical view of safety in laparoscopic cholecystectomy. *Intelligence-Based Medicine* **10**, 100153 (2024).

85. Leifman, G., Aides, A., Golany, T., Freedman, D. & Rivlin, E. Pixel-accurate Segmentation of Surgical Tools based on Bounding Box Annotations. in *2022 26th International Conference on Pattern Recognition (ICPR)* 5096–5103 (IEEE, Montreal, QC, Canada, 2022). doi:10.1109/ICPR56361.2022.9956530.

86. Li, J. *et al.* Recognize Any Surgical Object: Unleashing the Power of Weakly-Supervised Data. Preprint at https://doi.org/10.48550/ARXIV.2501.15326 (2025).

87. Li, Y. *et al.* Automated Assessment of Critical View of Safety in Laparoscopic Cholecystectomy. in *2023 IEEE 11th International Conference on Healthcare Informatics (ICHI)* 330–337 (IEEE, Houston, TX, USA, 2023). doi:10.1109/ICHI57859.2023.00051.

88. Liao, G., Jogan, M., Koushik, S., Eaton, E. & Hashimoto, D. A. Disentangling spatio-temporal knowledge for weakly supervised object detection and segmentation in surgical video. Preprint at https://doi.org/10.48550/ARXIV.2407.15794 (2024).

89. Lin, W. *et al.* Instrument-tissue Interaction Detection Framework for Surgical Video Understanding. Preprint at https://doi.org/10.48550/ARXIV.2404.00322 (2024).

90. Lin, S. *et al.* Multi-Frame Feature Aggregation for Real-Time Instrument Segmentation in Endoscopic Video. *IEEE Robot. Autom. Lett.* **6**, 6773–6780 (2021).

91. Liu, R. *et al.* Artificial intelligence in laparoscopic cholecystectomy: does computer vision outperform human vision? *Art Int Surg* **2**, 80–92 (2022).

92. Liu, M. *et al.* LSKANet: Long Strip Kernel Attention Network for Robotic Surgical Scene Segmentation. *IEEE Trans. Med. Imaging* **43**, 1308–1322 (2024).

93. Liu, Y., Zhao, Z., Shi, P. & Li, F. Towards Surgical Tools Detection and Operative Skill Assessment

Based on Deep Learning. *IEEE Trans. Med. Robot. Bionics* **4**, 62–71 (2022).

94. Lou, A., Tawfik, K., Yao, X., Liu, Z. & Noble, J. Min-Max Similarity: A Contrastive Semi-Supervised Deep Learning Network for Surgical Tools Segmentation. Preprint at https://doi.org/10.48550/ARXIV.2203.15177 (2022).

95. Loukas, C. & Sgouros, N. P. Multi-instance multi-label learning for surgical image annotation. *Robotics Computer Surgery* **16**, e2058 (2020).

96. Loza, G., Valdastri, P. & Ali, S. Real-time surgical tool detection with multi-scale positional encoding and contrastive learning. *Healthcare Tech Letters* **11**, 48–58 (2024).

97. Maack, L., Behrendt, F., Bhattacharya, D., Latus, S. & Schlaefer, A. Efficient Anatomy Segmentation in Laparoscopic Surgery using Multi-Teacher Knowledge Distillation. in *Proceedings of The 7nd International Conference on Medical Imaging with Deep Learning* (eds. Burgos, N. et al.) vol. 250 937–948 (PMLR, 2024).

98. Madad Zadeh, S. *et al.* SurgAI: deep learning for computerized laparoscopic image understanding in gynaecology. *Surg Endosc* **34**, 5377–5383 (2020).

99. Madani, A. *et al.* Artificial Intelligence for Intraoperative Guidance: Using Semantic Segmentation to Identify Surgical Anatomy During Laparoscopic Cholecystectomy. *Annals of Surgery* **276**, 363–369 (2022).

100. Maqbool, S., Riaz, A., Sajid, H. & Hasan, O. m2caiSeg: Semantic Segmentation of Laparoscopic Images using Convolutional Neural Networks. Preprint at https://doi.org/10.48550/ARXIV.2008.10134 (2020).

101. Marullo, G., Tanzi, L., Ulrich, L., Porpiglia, F. & Vezzetti, E. A Multi-Task Convolutional Neural Network for Semantic Segmentation and Event Detection in Laparoscopic Surgery. *JPM* **13**, 413 (2023).

102. Mascagni, P. *et al.* Artificial Intelligence for Surgical Safety: Automatic Assessment of the Critical View of Safety in Laparoscopic Cholecystectomy Using Deep Learning. *Annals of Surgery* **275**, 955–961 (2022).

103. Matsumoto, S. *et al.* Laparoscopic distal gastrectomy skill evaluation from video: a new artificial intelligence-based instrument identification system. *Sci Rep* **14**, 12432 (2024).

104. Mehta, P. *et al.* Hierarchical segmentation of surgical scenes in laparoscopy. *Int J CARS* **19**, 1449–1457 (2024).

105. Mishra, K., Sathish, R. & Sheet, D. Learning Latent Temporal Connectionism of Deep Residual Visual Abstractions for Identifying Surgical Tools in Laparoscopy Procedures. in *2017 IEEE Conference on Computer Vision and Pattern Recognition Workshops (CVPRW)* 2233–2240 (IEEE, Honolulu, HI, USA, 2017). doi:10.1109/CVPRW.2017.277.

106. Murali, A., Mascagni, P., Mutter, D. & Padoy, N. CycleSAM: One-Shot Surgical Scene Segmentation using Cycle-Consistent Feature Matching to Prompt SAM. Preprint at https://doi.org/10.48550/ARXIV.2407.06795 (2024).

107. Murali, A. *et al.* Encoding Surgical Videos as Latent Spatiotemporal Graphs for Object and Anatomy-Driven Reasoning. (2023) doi:10.48550/ARXIV.2312.06829.

108. Murali, A. *et al.* Latent Graph Representations for Critical View of Safety Assessment. Preprint at https://doi.org/10.48550/ARXIV.2212.04155 (2022).

109. Nyi Myo, N., Boonkong, A., Khampitak, K. & Hormdee, D. Real-Time Surgical Instrument Segmentation Analysis Using YOLOv8 With ByteTrack for Laparoscopic Surgery. *IEEE Access* **12**, 83091–83103 (2024).

110. Nakanuma, H. *et al.* An intraoperative artificial intelligence system identifying anatomical landmarks for laparoscopic cholecystectomy: a prospective clinical feasibility trial (J-SUMMIT-C-01). *Surg Endosc* **37**, 1933–1942 (2023).

111. Namazi, B., Sankaranarayanan, G. & Devarajan, V. A contextual detector of surgical tools in laparoscopic videos using deep learning. *Surg Endosc* **36**, 679–688 (2022).

112. Nema, S. & Vachhani, L. Unpaired deep adversarial learning for multi-class segmentation of instruments in robot-assisted surgical videos. *Robotics Computer Surgery* **19**, e2514 (2023).

113. Nwoye, C. I. & Padoy, N. SurgiTrack: Fine-Grained Multi-Class Multi-Tool Tracking in Surgical Videos. (2024) doi:10.48550/ARXIV.2405.20333.

114. Nwoye, C. I., Mutter, D., Marescaux, J. & Padoy, N. Weakly supervised convolutional LSTM approach for tool tracking in laparoscopic videos. *Int J CARS* **14**, 1059–1067 (2019).

115. Oh, N. *et al.* Real-time segmentation of biliary structure in pure laparoscopic donor hepatectomy. *Sci Rep* **14**, 22508 (2024).

116. Owen, D., Grammatikopoulou, M., Luengo, I. & Stoyanov, D. Automated identification of critical structures in laparoscopic cholecystectomy. *Int J CARS* **17**, 2173–2181 (2022).

117. Ozbulak, U. *et al.* Less is More? Revisiting the Importance of Frame Rate in Real-Time Zero-Shot Surgical Video Segmentation. Preprint at https://doi.org/10.48550/ARXIV.2502.20934 (2025).

118. Pan, X., Bi, M., Wang, H., Ma, C. & He, X. DBH-YOLO: a surgical instrument detection method based on feature separation in laparoscopic surgery. *Int J CARS* **19**, 2215–2225 (2024).

119. Penza, V. *et al.* Long Term Safety Area Tracking (LT-SAT) with online failure detection and recovery for robotic minimally invasive surgery. *Medical Image Analysis* **45**, 13–23 (2018).

120. Pradeep, C. S. & Sinha, N. Multi-Tasking DSSD Architecture for Laparoscopic Cholecystectomy Surgical Assistance Systems. in *2022 IEEE 19th International Symposium on Biomedical Imaging (ISBI)* 1–4 (IEEE, Kolkata, India, 2022). doi:10.1109/ISBI52829.2022.9761562.

121. Prokopetc, K., Collins, T. & Bartoli, A. Automatic Detection of the Uterus and Fallopian Tube Junctions in Laparoscopic Images. in *Information Processing in Medical Imaging* (eds. Ourselin, S., Alexander, D. C., Westin, C.-F. & Cardoso, M. J.) vol. 9123 552–563 (Springer International Publishing, Cham, 2015).

122. Protserov, S. *et al.* Development, deployment and scaling of operating room-ready artificial intelligence for real-time surgical decision support. *npj Digit. Med.* **7**, 231 (2024).

123. Rahbar, M. D., Reisner, L., Ying, H. & Pandya, A. An entropy-based approach to detect and localize intraoperative bleeding during minimally invasive surgery. *Robotics Computer Surgery* **16**, 1–9 (2020).

124. Daneshgar Rahbar, M., Pappas, G. & Jaber, N. Toward Intraoperative Visual Intelligence: Real-Time Surgical Instrument Segmentation for Enhanced Surgical Monitoring. *Healthcare* **12**, 1112 (2024).

125. Raja, M. A., Loughran, R. & McCaffery, F. Cholecystectomy Surgical Instrument Detection using Variants of YOLOv8. in *2024 35th Irish Signals and Systems Conference (ISSC)* 01–06 (IEEE, Belfast, United Kingdom, 2024). doi:10.1109/ISSC61953.2024.10603096.

126. Ryu, K. *et al.* Deep learning-based vessel automatic recognition for laparoscopic right hemicolectomy. *Surg Endosc* **38**, 171–178 (2024).

127. Sahu, M., Mukhopadhyay, A., Szengel, A. & Zachow, S. Addressing multi-label imbalance problem of surgical tool detection using CNN. *Int J CARS* **12**, 1013–1020 (2017).

128. Samuel, D. J. & Cuzzolin, F. Unsupervised Anomaly Detection for a Smart Autonomous Robotic Assistant Surgeon (SARAS) Using a Deep Residual Autoencoder. *IEEE Robot. Autom. Lett.* **6**, 7256–7261 (2021).

129. Sanchez-Matilla, R., Robu, M., Grammatikopoulou, M., Luengo, I. & Stoyanov, D. Data-centric multi-task surgical phase estimation with sparse scene segmentation. *Int J CARS* **17**, 953–960 (2022).

130. Satyanaik, S. *et al.* Optimizing latent graph representations of surgical scenes for unseen domain generalization. *Int J CARS* **19**, 1243–1250 (2024).

131. Satyanaik, S. *et al.* Optimizing Latent Graph Representations of Surgical Scenes for Zero-Shot Domain Transfer. Preprint at https://doi.org/10.48550/ARXIV.2403.06953 (2024).

132. Seenivasan, L., Islam, M., Kannan, G. & Ren, H. SurgicalGPT: End-to-End Language-Vision GPT for Visual Question Answering in Surgery. Preprint at https://doi.org/10.48550/ARXIV.2304.09974 (2023).

133. Sengun, B. *et al.* Artificial Intelligence in Minimally Invasive Adrenalectomy: Using Deep Learning to Identify the Left Adrenal Vein. *Surgical Laparoscopy, Endoscopy & Percutaneous Techniques* **33**, 327–331 (2023).

134. Sengun, B. *et al.* Utilization of artificial intelligence in minimally invasive right adrenalectomy: recognition of anatomical landmarks with deep learning. *Acta Chirurgica Belgica* **124**, 492–498 (2024).

135. Shen, W. *et al.* Branch Aggregation Attention Network for Robotic Surgical Instrument Segmentation. *IEEE Trans. Med. Imaging* **42**, 3408–3419 (2023).

136. Sheng, M., Fan, J., Liu, D., Kikinis, R. & Cai, W. Revisiting Surgical Instrument Segmentation Without Human Intervention: A Graph Partitioning View. Preprint at https://doi.org/10.48550/ARXIV.2408.14789 (2024).

137. Shi, P., Zhao, Z., Hu, S. & Chang, F. Real-Time Surgical Tool Detection in Minimally Invasive Surgery Based on Attention-Guided Convolutional Neural Network. *IEEE Access* **8**, 228853–228862 (2020).

138. Shimgekar, S. R., Pathi, P. R. & V, S. Voice based Segmentation of Laparoscopic Surgical Tools and its Image Enhancement. in *2021 Innovations in Power and Advanced Computing Technologies (i-PACT)* 1–7 (IEEE, Kuala Lumpur, Malaysia, 2021). doi:10.1109/i-PACT52855.2021.9696600.

139. Silva, B. *et al.* Analysis of Current Deep Learning Networks for Semantic Segmentation of Anatomical Structures in Laparoscopic Surgery. in *2022 44th Annual International Conference of the IEEE Engineering in Medicine & Biology Society (EMBC)* 3502–3505 (IEEE, Glasgow, Scotland, United Kingdom, 2022). doi:10.1109/EMBC48229.2022.9871583.

140. Smithmaitrie, P. *et al.* Development of deep learning framework for anatomical landmark detection and guided dissection line during laparoscopic cholecystectomy. *Heliyon* **10**, e25210 (2024).

141. Sonsilphong, S., Sonsilphong, A., Hormdee, D. & Khampitak, K. A Development of Object Detection System based on Deep Learning Approach to Support the Laparoscope Manipulating Robot (LMR). in *2022 International Electrical Engineering Congress (iEECON)* 1–4 (IEEE, Khon Kaen, Thailand, 2022). doi:10.1109/iEECON53204.2022.9741676.

142. Streckert, T., Fromme, D., Kaupenjohann, M. & Thiem, J. Using Synthetic Data to Increase the Generalization of a CNN for Surgical Instrument Segmentation. in *2023 IEEE 12th International Conference on Intelligent Data Acquisition and Advanced Computing Systems: Technology and Applications (IDAACS)* 336–340 (IEEE, Dortmund, Germany, 2023). doi:10.1109/IDAACS58523.2023.10348781.

143. Strong, J. S. *et al.* Evaluating surgical expertise with AI -based automated instrument recognition for robotic distal gastrectomy. *Annals of Gastroent Surgery* **8**, 611–619 (2024).

144. Sun, Y., Pan, B. & Fu, Y. Lightweight Deep Neural Network for Articulated Joint Detection of Surgical Instrument in Minimally Invasive Surgical Robot. *J Digit Imaging* **35**, 923–937 (2022).

145. Takeuchi, M. *et al.* Towards automatic verification of the critical view of the myopectineal orifice with artificial intelligence. *Surg Endosc* **37**, 4525–4534 (2023).

146. Tao, R., Zou, X. & Zheng, G. LAST: LAtent Space-Constrained Transformers for Automatic Surgical Phase Recognition and Tool Presence Detection. *IEEE Trans. Med. Imaging* **42**, 3256–3268 (2023).

147. Tokuyasu, T. *et al.* Development of an artificial intelligence system using deep learning to indicate anatomical landmarks during laparoscopic cholecystectomy. *Surg Endosc* **35**, 1651–1658 (2021).

148. Urrea, C., Garcia-Garcia, Y. & Kern, J. Improving Surgical Scene Semantic Segmentation through a Deep Learning Architecture with Attention to Class Imbalance. *Biomedicines* **12**, 1309 (2024).

149. Valderrama, N. *et al.* Towards Holistic Surgical Scene Understanding. (2022) doi:10.48550/ARXIV.2212.04582.

150. Vardazaryan, A., Mutter, D., Marescaux, J. & Padoy, N. Weakly-Supervised Learning for Tool Localization in Laparoscopic Videos. Preprint at https://doi.org/10.48550/ARXIV.1806.05573 (2018).

151. Wagner, M. *et al.* Comparative validation of machine learning algorithms for surgical workflow and skill analysis with the HeiChole benchmark. *Medical Image Analysis* **86**, 102770 (2023).

152. Wang, Y., He, G. & Ahmed, M. F. A Real-Time Laparoscopic Surgical Instrument Detection System Based on YOLOv5. in *2023 16th International Congress on Image and Signal Processing, BioMedical Engineering and Informatics (CISP-BMEI)* 1–6 (IEEE, Taizhou, China, 2023). doi:10.1109/CISP-BMEI60920.2023.10373249.

153. Wang, S., Raju, A. & Huang, J. Deep learning based multi-label classification for surgical tool presence detection in laparoscopic videos. in *2017 IEEE 14th International Symposium on Biomedical Imaging (ISBI 2017)* 620–623 (IEEE, Melbourne, Australia, 2017). doi:10.1109/ISBI.2017.7950597.

154. Wang, H., Xu, H., Wang, J., Zhou, J. & Deng, K. Efficient Surgical Tool Recognition via HMM-Stabilized Deep Learning. Preprint at https://doi.org/10.48550/ARXIV.2404.04992 (2024).

155. Wang, Y. *et al.* Evaluating robotic-assisted partial nephrectomy surgeons with fully convolutional segmentation and multi-task attention networks. *J Robotic Surg* **17**, 2323–2330 (2023).

156. Wang, Q., Zhao, S., Xu, Z. & Zhou, S. K. LACOSTE: Exploiting stereo and temporal contexts for surgical instrument segmentation. *Medical Image Analysis* **99**, 103387 (2025).

157. Wang, Y., Sun, Q., Sun, G., Gu, L. & Liu, Z. Object Detection of Surgical Instruments Based on YOLOv4. in *2021 6th IEEE International Conference on Advanced Robotics and Mechatronics (ICARM)* 578–581 (IEEE, Chongqing, China, 2021). doi:10.1109/ICARM52023.2021.9536075.

158. Wang, G. & Wang, S. Surgical Tools Detection Based on Training Sample Adaptation in Laparoscopic Videos. *IEEE Access* **8**, 181723–181732 (2020).

159. Wang, Q., Liu, Y., Zhao, S., Liu, R. & Zhou, S. K. WeakSurg: Weakly supervised surgical instrument segmentation using temporal equivariance and semantic continuity. Preprint at https://doi.org/10.48550/ARXIV.2403.09551 (2024).

160. Wang, W., Luo, Y., Wang, J., Wang, X. & Song, H. ToolNet-X: Surgical Instrument Detection Combined With High-Order Spatial Interaction. *IEEE Trans. Med. Robot. Bionics* **5**, 857–866 (2023).

161. Ward, T. M., Hashimoto, D. A., Ban, Y., Rosman, G. & Meireles, O. R. Artificial intelligence prediction of cholecystectomy operative course from automated identification of gallbladder inflammation. *Surg Endosc* **36**, 6832–6840 (2022).

162. Wei, M., Shi, M. & Vercauteren, T. Enhancing surgical instrument segmentation: integrating vision transformer insights with adapter. *Int J CARS* **19**, 1313–1320 (2024).

163. Wei, M. *et al.* SegMatch: A semi-supervised learning method for surgical instrument segmentation. (2023) doi:10.48550/ARXIV.2308.05232.

164. Wu, R. *et al.* MCF-SMSIS: Multi-tasking with complementary functions for stereo matching and surgical instrument segmentation. *Computers in Biology and Medicine* **179**, 108923 (2024).

165. Xi, N., Meng, J. & Yuan, J. Forest Graph Convolutional Network for Surgical Action Triplet Recognition in Endoscopic Videos. *IEEE Trans. Circuits Syst. Video Technol.* **32**, 8550–8561 (2022).

166. Xu, J. *et al.* Personalizing Federated Instrument Segmentation with Visual Trait Priors in Robotic Surgery. Preprint at https://doi.org/10.48550/ARXIV.2408.03208 (2024).

167. Xue, Y., Li, Y., Liu, S., Wang, P. & Qian, X. Oriented Localization of Surgical Tools by Location Encoding. *IEEE Trans. Biomed. Eng.* **69**, 1469–1480 (2022).

168. Yamazaki, Y. *et al.* Automated Surgical Instrument Detection from Laparoscopic Gastrectomy Video Images Using an Open Source Convolutional Neural Network Platform. *Journal of the American College of Surgeons* **230**, 725-732e1 (2020).

169. Yamlahi, A. *et al.* Self-distillation for surgical action recognition. Preprint at https://doi.org/10.48550/ARXIV.2303.12915 (2023).

170. Yang, Z. *et al.* Detection of Anatomical Landmarks During Laparoscopic Cholecystectomy Surgery Based on Improved Yolov7 Algorithm. in *2024 IEEE 13th Data Driven Control and Learning Systems Conference (DDCLS)* 251–256 (IEEE, Kaifeng, China, 2024). doi:10.1109/DDCLS61622.2024.10606792.

171. Yang, H. *et al.* Uncertainty Estimation for Safety-critical Scene Segmentation via Fine-grained Reward Maximization. Preprint at https://doi.org/10.48550/ARXIV.2311.02719 (2023).

172. Yang, Y.-J., Vadivelu, A. N., Pilgrim, C. H. C., Kulic, D. & Abdi, E. A Novel Perception Framework for Automatic Laparoscope Zoom Factor Control Using Tool Geometry. in *2021 43rd Annual International Conference of the IEEE Engineering in Medicine & Biology Society (EMBC)* 4700–4704 (IEEE, Mexico, 2021). doi:10.1109/EMBC46164.2021.9629987.

173. Yin, L. *et al.* Hypergraph-Transformer (HGT) for Interactive Event Prediction in Laparoscopic and Robotic Surgery. Preprint at https://doi.org/10.48550/ARXIV.2402.01974 (2024).

174. Yuan, K. *et al.* Advancing surgical VQA with scene graph knowledge. *Int J CARS* **19**, 1409–1417 (2024).

175. Zhang, J. *et al.* Graph-Based Spatial Reasoning for Tracking Landmarks in Dynamic Laparoscopic Environments. *IEEE Robot. Autom. Lett.* **9**, 8459–8466 (2024).

176. Zhang, Y., Kim, M. & Jin, S. Real-time Detection and Tracking of Surgical Instrument Based on YOLOv5 and DeepSORT[*]. in *2023 32nd IEEE International Conference on Robot and Human Interactive Communication (RO-MAN)* 1758–1763 (IEEE, Busan, Korea, Republic of, 2023). doi:10.1109/RO-MAN57019.2023.10309495.

177. Zhang, B. *et al.* Surgical instrument recognition for instrument usage documentation and surgical video library indexing. *Computer Methods in Biomechanics and Biomedical Engineering: Imaging & Visualization* **11**, 1064–1072 (2023).

178. Zhang, B., Wang, S., Dong, L. & Chen, P. Surgical Tools Detection Based on Modulated Anchoring Network in Laparoscopic Videos. *IEEE Access* **8**, 23748–23758 (2020).

179. Zhang, J. *et al.* Laparoscopic Image-Based Critical Action Recognition and Anticipation With Explainable Features. *IEEE J. Biomed. Health Inform.* **27**, 5393–5404 (2023).

180. Zhang, J. & Gao, X. Object extraction via deep learning-based marker-free tracking framework of surgical instruments for laparoscope-holder robots. *Int J CARS* **15**, 1335–1345 (2020).

181. Zhao, Z. *et al.* Anchor-guided online meta adaptation for fast one-Shot instrument segmentation from robotic surgical videos. *Medical Image Analysis* **74**, 102240 (2021).

182. Zhao, X. *et al.* CLAD-Net: cross-layer aggregation attention network for real-time endoscopic instrument detection. *Health Inf Sci Syst* **11**, 58 (2023).

183. Zhao, Z. *et al.* One to Many: Adaptive Instrument Segmentation via Meta Learning and Dynamic Online Adaptation in Robotic Surgical Video. Preprint at https://doi.org/10.48550/ARXIV.2103.12988 (2021).

184. Zhao, Z., Chen, Z., Voros, S. & Cheng, X. Real-time tracking of surgical instruments based on spatio-temporal context and deep learning. *Computer Assisted Surgery* **24**, 20–29 (2019).

185. Zheng, Q. *et al.* Development and validation of a deep-learning based assistance system for enhancing laparoscopic control level. *Robotics Computer Surgery* **19**, e2449 (2023).

186. Zhou, S. *et al.* Surgical Region of Interest Detection from Laparoscopic Images Using Boundary Fuzziness. in *2024 IEEE International Symposium on Biomedical Imaging (ISBI)* 1–5 (IEEE, Athens, Greece, 2024). doi:10.1109/ISBI56570.2024.10635366.

187. Zhou, Y. & Liu, Z. Detection of Surgical Instruments Based on YOLOv5. in *2022 IEEE International Conference on Manipulation, Manufacturing and Measurement on the Nanoscale (3M-NANO)* 470–473 (IEEE, Tianjin, China, 2022). doi:10.1109/3M-NANO56083.2022.9941507.

188. Zygomalas, A., Kalles, D., Katsiakis, N., Anastasopoulos, A. & Skroubis, G. Artificial Intelligence Assisted Recognition of Anatomical Landmarks and Laparoscopic Instruments in Transabdominal Preperitoneal Inguinal Hernia Repair. *Surg Innov* **31**, 178–184 (2024).
